# Quality of life domains revised by people with multiple sclerosis and healthcare professionals for adaptive measure development

**DOI:** 10.1371/journal.pone.0349034

**Published:** 2026-06-11

**Authors:** Ambra Mara Giovannetti, Andrea Giordano, Edoardo Donarelli, Giulia Di Domenico, Giampaolo Brichetto, Eleonora Cocco, Francesco Patti, Rachele Paolucci, Alessandra Solari, Silvia Testa, Rosalba Rosato, on behalf of the INITIALISE project

**Affiliations:** 1 Department of Psychology, University of Turin, Turin, Italy; 2 Neurology, Public Health and Disability Unit, Fondazione IRCCS Istituto Neurologico Carlo Besta, Milan, Italy; 3 Unit of Neuroepidemiology, Fondazione IRRCS Istituto Neurologico Carlo Besta, Milan, Italy; 4 Scientific Research Area, Italian Multiple Sclerosis Foundation (FISM), Genoa, Italy; 5 AISM Rehabilitation Service, Italian Multiple Sclerosis Society, Genoa, Italy; 6 Department of Medical Science and Public Health, University of Cagliari, Cagliari, Italy; 7 Multiple Sclerosis Center, ASL Cagliari, Cagliari, Italy; 8 Department of Medical and Surgical Sciences and Advanced Technologies “GF Ingrassia”, University of Catania, Catania, Italy; 9 UOS Multiple Sclerosis, Neurology Clinic, “G. Rodolico-San Marco” University Hospital, Catania, Italy; 10 Italian Multiple Sclerosis Society (AISM), Genoa, Italy; 11 Department of Human and Social Sciences, University of Aosta Valley, Aosta, Italy; University of Cagliari, ITALY

## Abstract

**Background:**

Multiple sclerosis (MS) significantly affects individuals’ health-related quality of life (HRQoL). Ideally, HRQoL assessment tools for MS should be patient-centered, ensuring that each item meaningfully contributes to measurement precision. The present study has three main objectives: (1) a review of the literature to identify key HRQoL domains by examining existing instruments; (2) confirmation and integration of HRQoL domains based on stakeholders’ input; (3) systematic comparison of the categories and subcategories identified by the stakeholders with the domains identified in the literature review.

**Methods:**

Findings from the literature review were discussed in four focus group meetings (FGMs) with key stakeholders, including people with MS (PwMS) and healthcare professionals (HPs). Participants were purposively sampled to ensure diversity in geographic location, gender, education, and (for PwMS) disease severity. FGMs were audio-recorded, transcribed verbatim, and analyzed using qualitative content analysis by two independent researchers. The categories and subcategories generated from the qualitative analysis were then systematically compared with the items of the MS-specific HRQoL instruments identified in the literature review, by two independent researchers.

**Results:**

Out of 5190 references, 308 records related to 9 instruments were included in the literature review. The main HRQoL domains were then extracted and discussed with the steering committee, and informed the FGM grids. The results validated the HRQoL domains identified in literature and provided deeper insights into their complexity.

**Conclusion:**

This study confirmed and added granularity to the existing HRQoL domains relevant to PwMS, identifying key areas for inclusion in future research. The findings contribute to the development of a more comprehensive, patient-centered HRQoL assessment tool, which could inform targeted interventions to enhance quality of life in PwMS.

## Introduction

Multiple sclerosis (MS) is a chronic demyelinating disease of the central nervous system, the most frequent cause of neurological disability in young adults, characterized by an unpredictable pathological process and progressive degeneration [1–3]. Clinical onset is generally in the most productive age of life, usually between 20 and 40 years [4]. The disease has a pervasive effect on physical, cognitive and psychological functioning and well-being [5–7]. Fatigue, pain, depression, and cognitive dysfunction are some of the multiple symptoms impaired by MS that can affect various areas of life for people with MS (PwMS), such as the ability to work and/or perform activities of daily living, but are difficult for healthcare providers to quantify because these symptoms are subjective, fluctuating, and lack objective clinical markers, making them hard to measure consistently or compare across patients [8].

In recent years, there has been a growing awareness among clinical authorities and regulators to use outcome measures that are meaningful to patients themselves, potentially expressed through the patient voice, when evaluating new treatments. In light of this medical trend, health-related quality of life (HRQoL) measurement instruments have gradually gained more attention [9]. Leidy et al. defined HRQoL as “the subjective perception of the impact of disease and treatment across the physical, psychological, social functioning and well-being” [10]. Studying HRQoL means exploring how patients experience and live with the manifestations of their disease in their daily lives, adding a new perspective to the clinician-based evaluation of the presence and severity of symptoms [11, 12]. This can be particularly important for people living with chronic diseases for which there is no cure and whose treatment is often lengthy and polytherapeutic [10]. The aim of HRQoL instruments is to provide a more comprehensive and personalized way to measure the impact of the disease, improving the detection of aspects that are more likely to go unnoticed. This helps clinicians to recognize patients’ priorities and facilitates communication between clinician and patient, thus promoting shared decision-making [9]. However, these tools are rarely used in frontline MS services and integrated into clinical practice [13]. This is due to potential logistical and financial issues associated with their administration, processing, interpretation, storage, and retrieval [14, 15]. Moreover, cultural and relational factors, such as hierarchical structures in medicine and asymmetrical patient–clinician relationships, may also hinder the routine use of HRQoL tools [16]. Other challenges are the fixed-length, paper-pencil format of most HRQoL instruments, as opposed to the need to collect data in a timely and understandable manner to reduce respondent burden and missing data, increasing measurement accuracy and the opportunity to share findings during the medical encounter.

Computer-Adaptive Tests (CAT) [17], delivered via a digital questionnaire that dynamically selects only the most relevant items based on patient responses, have been applied to HRQoL assessment [18], as their tailored format is thought to reduce the impact of cognitive fatigue while increasing acceptability and relevance for PwMS [19, 20]. The growing interest in CAT instruments in HRQoL research has led to the development of a range of CAT instruments by the Patient-Reported Outcomes Measurement Information System (PROMIS) supported by the National Institute of Health [21], which currently offers 3–10 items for domains such as fatigue, physical functioning, pain, depression, anxiety, and social functioning (www.nihpromis.org). For the assessment of multidimensional constructs with correlated dimensions, the Multidimensional Computer Adaptive Testing (MCAT) approach is more appropriate, but, as far as we know, there is few evidence of HRQoL instruments using such a methodology to fixed-length HRQOL questionnaires, including the MS domain [22–25].

To address this gap, we took a comprehensive approach based on a literature review and informed by the patient voice to identify relevant HRQoL dimensions that ensure both content validity and clinical utility of the assessment [21, 26, 27]. To identify the most relevant HRQoL domains in MS, (1) we first conducted a systematic literature review to identify HRQoL domains, which were then (2) discussed in focus group meetings (FGMs) with PwMS and healthcare professionals (HPs), to ensure the inclusion of most meaningful domains for them and possibly integrate them with additional relevant domains. Finally, (3) we systematically compared the categories and subcategories generated from the FGM analysis with the domains identified in the literature review.

A systematic review of the literature provides a useful basis for identifying commonly assessed HRQoL domains in MS.

There is evidence of the importance of including patients’ perspectives and insights in patient-reported outcomes (PROs), as this can help to identify potential issues related to domain coverage and/or inadequate operationalization [28–40]. This patient-centered approach ensures the inclusion of domains that may be underrepresented in existing instruments but are highly relevant to people living with the disease and increases the content validity of the questionnaire [41, 42]. However, in the MS context, the perspective of PwMS has often been overlooked in the development of existing PROs, such as for example the Multiple Sclerosis Quality of Life-54 items (MSQoL-54) [43], as well as the Functional Index for Living with Multiple Sclerosis (FILMS) [44], and the RAYS [45] instruments. As a result, important information about what truly matters to them is missing [46, 47].

A well-defined domain structure of HRQoL in MS may be useful for several future research directions, including the development of psychometrically sound item banks that allow for accurate, efficient, and individualized assessment, e.g., using the MCAT approach [48, 49], as well as studies aimed at capturing and ranking the priorities that patients assign to these domains.

Existing MS-specific HRQoL instruments—such as the MSQoL-54, Multiple Sclerosis Impact Scale (MSIS-29) [50], Multiple Sclerosis International Quality of Life questionnaire (MusiQoL) [51], and Functional Assessment of Multiple Sclerosis (FAMS) [52]—typically assess domains including physical functioning, fatigue, pain, cognition, emotional well-being, and social relationships. However, these instruments differ in scope, structure, and conceptual coverage, and may not fully align with the domains that PwMS describe as most meaningful, such as self-perception, social participation, work-related issues, and contextual or relational influences on daily life [46]. These underrepresented areas suggest that the current conceptualization of HRQoL in MS may not fully reflect patients’ lived experiences.

By integrating evidence from the literature with qualitative insights from PwMS and HPs, the present study aims to refine and expand the conceptual framework of HRQoL in MS, thereby enhancing its content validity and ensuring stronger patient relevance for future adaptive measurement development.

## Methods and study design

The project is based on the participatory governance model proposed by the MULTI-ACT Guidelines which involves the establishment of a working group called the Engagement Coordination Team (ECT) [53]. This is a multidisciplinary team representing key stakeholders for the study (researchers, clinicians, experts in the MULTI-ACT model, and importantly, patients – in our case, PwMS) who collaborate in the research to capture the disease experience and translate it into scientific knowledge, with the aim of improving, recalibrating, and potentially modifying the research project being undertaken.

The project is co-coordinated by the University of Turin (UNITO) and the Fondazione IRCCS Istituto Neurologico Carlo Besta (FINCB), Milan, and run in three Italian MS centres: FINCB; the Italian MS rehabilitation centre (AISM) and the Scientific Research Area (FISM), Genoa; and the ASL Cagliari/University of Cagliari (UNICA), Cagliari. The study was carried out in accordance with the Declaration of Helsinki recommendations. The protocol received ethical clearance from the ethics committees of the coordinating centres and the three enrolling centres: UNITO (21/11/2023, internal ref: 0636827); FINCB (30/10/2023, internal ref: CET 29/23); AISM (19/12/2024, internal ref: 791/2024); UNICA (11/03/2025, internal ref: 19/25). All participants gave written informed consent.

### Systematic review methods

We adhered to the PRISMA guidelines for the systematic review and reporting of outcome measurement instruments [54]. To identify key HRQoL domains, we included studies that utilized a multidimensional, MS-specific HRQoL instrument in adults with MS.

We developed a search strategy for PubMed (inception to September 2, 2023; no language restrictions) and adapted it for EMBASE, PsycINFO, CINAHL, and the online database for existing PROs (www.proqolid.org). Since we aimed to collect all MS-specific instruments that have been used to measure HRQoL in adults with MS, we used the relevant search strategy structure developed by the COSMIN initiative [55]. To identify additional studies, we also searched the studies included in published systematic reviews and the reference lists of the selected articles. Search terms included ‘health-related quality of life’, ‘quality of life’, ‘health profiles’, ‘well-being’, and ‘multiple sclerosis’. The search strategy is detailed in S1 File.

Two reviewers (AG and ED) conducted a pilot test with 10 articles to refine the eligibility criteria. Subsequently, the same reviewers independently screened the titles and abstracts for eligibility. Full-text articles of selected studies were then independently assessed by the reviewers. We considered all studies that provided information relevant to the conceptualization and use of HRQoL instruments in PwMS. Specifically, we included: observational and interventional studies employing HRQoL instruments as primary or secondary outcomes; and development and methodological papers describing instrument structure or domain composition. Systematic reviews, protocols, clinical guidelines, conference proceedings, and editorials were excluded.

#### Conceptual work and HRQoL domains mapping.

Data extraction was performed by two reviewers (AG and ED) and verified by a third reviewer (RR) using a bespoke electronic form.

We extracted key HRQoL domains from the MS-specific HRQoL instruments included in the review and mapped them using the HRQoL domains proposed by Leidy et al. [10]. All (sub)domains included in at least one of the tools cited in the selected literature were considered.

For each (sub)domain, we assessed item-related content to ensure alignment with its conceptual framework and to refine its characterization within the theoretical model. The identified (sub)domains were then illustrated in a figure, with revisions made to names and redundant (sub)domains consolidated.

The provisional HRQoL figure was reviewed during a steering committee (SC) meeting and revised based on feedback from the SC members. Subsequently, the figure was discussed with focus group facilitators to develop focus group meeting (FGM) guides and assist participants in understanding the structure of the domains.

### Qualitative study methods

The relevance of the key HRQoL domains identified in the literature was assessed, and additional relevant domains were explored through FGMs with PwMS and HPs. Two FGMs were conducted for each group of stakeholders. FGMs were selected because they facilitate the exploration of multiple perspectives simultaneously, while encouraging interaction, brainstorming, and the elaboration of ideas.

AMG and GDD devised the FGM guides (S2 File) with input from the SC.

#### Meeting with the ECT for finalizing the FGM guides.

Ten days before the meeting, ECT members received the two FGM guides in order to read and comment on them individually. Both consist of five sections which investigated the impact of MS on HRQOL. Field notes were taken and integrated accordingly to the issues identified to modify the guides.

#### FGM enrolment procedure and conduct.

Investigators at each participating centre identified potentially eligible PwMS from their database and invited them to participate in the study. Eligible MS HPs were identified by the SC from the participating centres and subsequently invited to join. FGM participants were selected through purposive sampling to ensure a broad range of perspectives and opinions. Participants were drawn from two geographic regions of Italy (North and South) and varied in terms of age, gender, education, and, for PwMS, neurological impairment (based on the Expanded Disability Status Scale, EDSS [56]), and years since MS diagnosis.

PwMS were included if they were at least 18 years of age, fluent in Italian, and provided written informed consent. Exclusion criteria for PwMS included severe cognitive impairment, as assessed by the referring neurologist, and an inability to communicate effectively.

HPs were eligible if they had experience in the care of pwMS (i.e., working in clinical or rehabilitative specialized settings and actively involved in the assistance, care, and support of PwMS), were fluent in Italian, and provided written informed consent.

Each FGM included 6–10 participants and two facilitators (AMG and GDD) and was conducted online via Zoom to enhance accessibility and feasibility for PwMS. Sessions followed a semi-structured guide covering pre-specified topics, were audio-recorded, fully transcribed, and subsequently reviewed by participants for validation.

#### Analysis.

Content analysis was used to code the FGM transcripts, employing a combination of deductive and inductive approaches. The deductive process, informed by findings from the literature review (existing HRQoL domains), applied category labels consistent with the literature when appropriate. Simultaneously, the inductive approach enabled the identification of novel themes that emerged directly from the data. This dual approach began with a deductive framework to structure the initial analysis while incorporating inductive coding to capture insights not anticipated in the literature, ensuring a more comprehensive and nuanced understanding of the data.

A line-by-line coding method ensured thorough and detailed analysis. A three-step coding scheme was implemented: Two researchers (AMG and GDD) analyzed the transcripts independently during steps 1 and 2, and collaboratively in step 3. In the first step, initial codes were extracted directly from participants’ written responses. The second step involved data aggregation, with each researcher independently generating labels for codes and categories. In the third step, the researchers discussed and refined the independently generated codes and categories [57]. The Consolidated Criteria for Reporting Qualitative Research (COREQ) [58] guided the presentation of findings, leading to the creation of a detailed report. The adherence to the COREQ checklist is documented in the S3 File. The results from this phase were reviewed by the SC and the ECT, which determined the final set of HRQoL domains and updated the HRQoL list accordingly.

### Cross-mapping of HRQoL instruments and qualitative findings

The categories and subcategories generated from the qualitative analysis were then systematically compared with the items of the MS-specific HRQoL instruments identified in the literature review. This comparison process was carried out by two reviewers (AG, ED), who independently matched (sub)domains, categories and subcategories to the instrument items using a bespoke electronic form; all matches were verified by a third reviewer (AMG). This final comparison step was undertaken to highlight which elements of the final set of HRQoL domains, categories, and subcategories were already captured—and which were not—within the items of the HRQoL instruments included in the review, thereby identifying potential gaps in the measurement of HRQoL in MS.

## Results

### Systematic review

We identified 5190 references and, with the initial screening, 4655 records were excluded. Five hundred thirty-five full-text references were assessed for eligibility and, of those, 227 were excluded because only abstract was available (n = 149), the study was not related to a multi-dimensional HRQoL instrument (n = 42), were reviews (n = 27), included wrong populations (n = 9). We included 308 references related to 9 instruments: Multiple Sclerosis Quality of Life-54 items (MSQoL-54) [43] (n = 134), Multiple Sclerosis Impact Scale (MSIS-29) [50] (n = 59), Functional Assessment of Multiple Sclerosis (FAMS) [52] (n = 49), Multiple Sclerosis International Quality of Life questionnaire (MusiQoL) [51] (n = 45), Hamburg Quality of Life questionnaire in Multiple Sclerosis (HAQUAMS) [59] (n = 17), Patient Reported outcome indices for Multiple Sclerosis (PRIMUS) [60] (n = 7), Short Quality of Life scale (SQOL) [61] (n = 4), RAYS [45] (n = 3), Functional Index for Living with Multiple Sclerosis (FILMS) [44] (n = 1). The PRISMA flow diagram is depicted in S4 File. The relevant domains were then extracted from these HRQOL instruments.

#### Conceptual work and HRQoL domains mapping.

After the SC meeting, a few symptoms (i.e., sensory impairments, balance and coordination, swallowing and spasticity) were added to the ‘physical domain’. The ‘psychological domain’ was slightly revised by splitting cognition and emotion subdomains, and deleting a few redundant subdomains (i.e., general contentment, mood).

After the meeting with FGM facilitators, subdomains overlapping the ‘psychological’ and the ‘social’ domains were slightly revised. Specifically, social well-being was considered redundant and, as such, was included in social support and social relationships, while self-perception in social settings was better specified.

**Fig 1:**
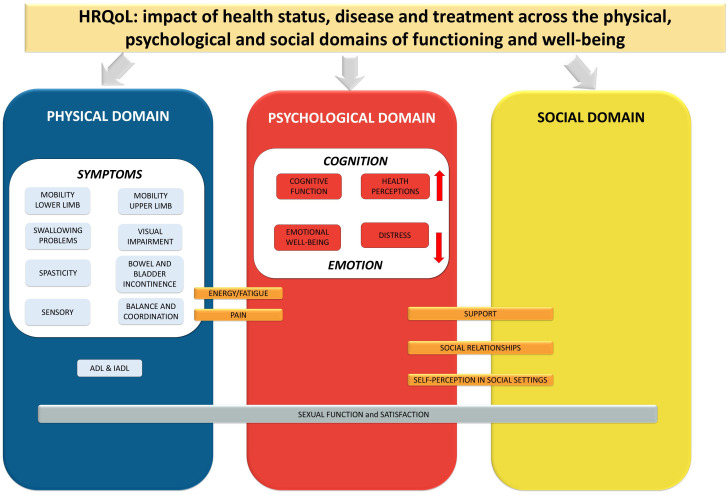
Key HRQoL (sub)domains extracted from the MS-specific HRQoL instruments included in the review.

All these revisions were integrated into the domain mapping (S5 File), resulting in the graphical representation shown in Fig 1.

The definitions of the ‘physical’, ‘psychological’, and ‘social’ domains were prepared and included in the FGM guides, each accompanied by a list of three representative items.

### Qualitative study

#### Meeting with the ECT for finalizing the FGM guides.

On February 2, 2025, a 2-hour online meeting was conducted with six PwMS (median age 35 years [range 31–64], 67% women, 84% single, all with college education and full-time employment, 67% with relapsing-remitting MS, median EDSS 2.0 [range 1.0–6.5], median years since MS diagnosis 11.5 [range 2–19]), and four researchers. The FGM guide was generally considered clear. Key modifications focused on rephrasing questions to better capture the impact of MS on HRQoL, particularly by refining how treatment and daily functioning were framed. Notable changes included adding ‘rehabilitation’ to the definition of MS treatment and clarifying the distinction between activities of daily living (ADL) and instrumental activities of daily living (IADL). Participants appreciated the inclusion of questions addressing both negative and positive influences on HRQoL. The HPs guide was adapted from the patient version. The revised guides are detailed in the S2 File.

#### FGMs participants and setting.

Between January 1, 2025 and March 31, 2025 we enrolled 18 PwMS and 20 HPs, across all study centers. Four online FGMs were conducted in April 2025, two with PwMS and two with HPs. The average duration of the FGMs was 144 minutes (SD 9.9) with PwMS, and 132 minutes (SD 14.1) with HPs. Among those enrolled, five PwMS and two HPs were unable to participate due to medical or logistical reasons.

PwMS (7 women, 7 men) had a median age of 52.5 years (range 36–64). EDSS scores ranged from 1.5 to 9.0 (median 4.0), and disease duration ranged from 5 months to 42 years (median 17). Half had a relapsing-remitting course and half a progressive form. Participants were recruited from Milano, Cagliari, and Genova, attending FGM 2 (n = 9) or FGM 3 (n = 5).

HPs (15 women, 3 men) had a median age of 50 years (range 30–65) and 3–35 years of experience in MS care (mean 19.6). They had followed a median of 100 PwMS in the past six months (range 12–600) and were employed across MS centres, rehabilitation centres, and AISM facilities, representing roles such as neurologists, nurses, physiotherapists, psychologists, and social workers. HPs participated in FGM 1 (n = 10) and FGM 4 (n = 8).

#### FGMs findings.

Qualitative data on HRQoL domains were collected during the four FGMs and organized into three themes: 1.1 Physical Domain; 1.2 Psychological Domain; 1.3 Social Domain.

This section presents each theme along with its main categories and illustrative quotes. The quotes include participants’ IDs in brackets: (ID: number, ‘P’ or ‘H’), where ‘P’ represents PwMS and ‘H’ denotes HPs. A complete list of categories, subcategories, and quotes is available in the audit trail (see S6 File).

#### HRQoL domains.

Table 1 highlights the key determinants of HRQoL across three domains, incorporating perspectives from both PwMS and HPs: Physical, Psychological, and Social. The FGM results confirmed all domains identified in the existing literature, but offered greater detail in terms of categories and subcategories within each domain.

**Table 1:** List of HRQoL Domains (only main categories).

Physical Domain	Psychological Domain	Social Domain
Symptoms (PwMS; HP)	Cognitive Function (PwMS; HP)	Support (PwMS; HP)
ADL (PwMS; HP)	Health Perceptions (PwMS; HP)	Social Connectedness (PwMS; HP)
IADL (PwMS; HP)	Emotional Well-Being (PwMS; HP)	Self-Perception in Social Settings (PwMS; HP)
Impact of the DMT on the body (HP)	MS is a Stressor (PwMS; HP)	Social Participation (PwMS; HP)
		Work (PwMS; HP)
		Finances (PwMS; HP)

ADL, activities of daily living; DMT, disease-modifying therapy; HP, health professional; HRQoL, health-related quality of life; IADL, instrumental activities of daily living; MS, multiple sclerosis; PwMS, people with multiple sclerosis.

*Physical Domain* – The Physical Domain includes symptoms (10 subcategories), ADL/IADL (two subcategories), and the impact of disease-modifying therapies (DMTs). For the full list of categories, subcategories, and quotes see S6 File: tables S3, S3.1 and S3.1.1.

In the Physical Domain, determinants of HRQoL for PwMS reveal significant challenges. Key symptoms include mobility impairments, with many reporting progressive difficulties*: ‘The illness makes itself felt, even though from the outside it’s not so noticeable’* (ID: 8P). Pain, particularly neuropathic pain, is frequently overlooked but severely impacts daily life: *‘Pain is often very disabling for PwMS’* (ID: 11H). Incontinence issues further complicate daily experiences, with one participant stating, *‘I stopped going out because I couldn’t control the situation’* (ID: 4P). Symptoms often limit ADL and IADL: *‘I can only manage to do one thing a day’* (ID: 6P). The impact of DMTs can also influence physical function, with one HP stated, *‘The type of drug absolutely changes daily life’* (ID: 14H).

*Psychological Domain –* Central factors affecting HRQoL in PwMS in the Psychological Domain include cognitive function, health perception (five subcategories), emotional well-being (seven subcategories), and stress related to MS. For the full list of categories, subcategories, and quotes see S6 File: tables S3, S3.2; S3.2.1). Participants reported feeling mentally slower and less sharp. One PwMS expressed this concern: *‘Mentally, I feel much slower, even in terms of attention. That, for me, is what the disease has affected the most.’* (ID: 10P). HPs also noted that cognitive impairment can significantly impact a person’s life: *‘Sometimes, even when physical limitations are relatively minor, the person might struggle with concentration, and that can have a major impact’* (ID: 8H). Another participant reflected on initial misconceptions related to illness representation and its impact on QoL: *‘Not knowing much about the disease, I immediately thought of a diagnosis leading to a wheelchair. And that was difficult to shake off as an impact, let’s say’* (ID: 4P).

Finally, a recently diagnosed PwMS pointed out how MS can act as stressor: *‘Certainly, [MS] is a source of stress because it is unpredictable—you don’t know how things will be today or a year from now. You can read all you want and hear all sorts of things, but no one can tell you how it will progress.’* (ID: 1P). Participants also highlighted that the ongoing management of the disease—including therapies, diagnostic tests, and regular medical appointments—can itself generate psychological strain: *‘One aspect that hasn’t been mentioned is the impact—not just physical, but also the effects of therapies and the planning of the entire diagnostic process on daily life… individuals suddenly feel they are in need of support from others and require assistance from healthcare professionals.*’ (ID: 6H). These concerns were further emphasized by HPs when describing the emotional impact of receiving the diagnosis: *‘The impact [of the diagnosis] is quite profound, as we know, because MS […] is one of those conditions that elicits fear and is frequently mentioned in news reports, alongside other incurable diseases. […] Thus, it is grouped with other major fears, such as cancer. Consequently, when a diagnosis is made, it signifies that ‘from this moment on, my life will be forever different, and I am, in fact, among the most unfortunate people in the world’* (ID: 17H).

*Social Domain –* The Social Domain includes Support (two subcategories), Social Connectedness (six subcategories), Self-Perception in Social Settings (three subcategories), Social Life (four subcategories), Work (four subcategories) and Finances (two subcategories). For the full list of categories, subcategories, and quotes see S6 File: tables S3, S3.3 and S3.3.1.

Support covers the assistance patients receive and the challenges they face in supporting others, especially in parenting and relationships. The psychological burden from MS can hinder empathy and limit the ability to seek help. One participant emphasized the importance of family, expressing concerns about not being able to engage in activities with their young child as they once could, saying, *‘I worry about how much I can physically give him… it’s different now’* (ID: 14P).

Social Connectedness reveals the impact of invisible symptoms like fatigue and incontinence on interactions. Medical care demands, fear of disclosure, and sexual dysfunction further isolate PwMS. One participant shared: *‘Fatigue limits opportunities for social interactions… I might manage to go out once a week at best’* (ID: 11P).

Self-Perception addresses stigma and feelings of vulnerability. One participant noted: *‘What bothers me more is how others perceive my situation… I’m less concerned about my experience than about judgment from others’* (ID: 14P).

Social Participation highlights restrictions in family activities, social life, and leisure. A nurse commented, *‘Treatments impact social life and energy levels… after a rehab session, they often need to rest, which cuts into their social time’ (ID: 16H).*

Work captures the challenges PwMS face in maintaining employment and navigating professional roles. Key issues include difficulties caused by MS symptoms (e.g., cognitive fatigue), changes in work activities, the burden of medical leave, and discrimination. One participant shared: *“Yes, definitely [MS cognitive symptoms affect QoL], both in my work and personal life. Sometimes I forget things that were said to me, and I struggle to follow conversations until the end. I lose track and realize I’ve missed part of the dialogue. I don’t know, I was a bit absent at times.” (ID: 7P).*

A paediatric nurse reflected on the professional consequences of treatment: *“My job is very important to me… When I started interferon therapy about 15 years ago, I had to give up my previous duties for lighter tasks. It wasn’t a choice; it was something I had to endure.” (ID: 12P).*

An HP added insight into structural challenges in the workplace: *“Patients often struggle to justify their medical needs, like physiotherapy or hospital visits, due to inadequate workplace accommodations. This need to constantly explain their condition disrupts their work-life balance and is a major challenge.” (ID: 15H).*

Financial concerns relate to both direct disease-management costs and the economic burden of work limitations. Participants described the financial strain of accessing ongoing rehabilitation, often requiring private services. One HP noted: *“Often, to receive continuous rehabilitation care, one must resort to private services, which can be quite costly. This financial burden is a significant concern.” (ID: 2H).* A PwMS reflected on the personal cost of self-employment with MS: *“I am self-employed, so when I take time off, I don’t receive payment. This concern has been present since the day I was hospitalized. With two children to support, I inquired about the length of my hospital stay because I knew these days would be counted as sick leave. Whenever I feel unwell, I make every effort to continue working because the financial impact is significant and a constant worry.” (ID: 1P).*

### Cross-mapping of HRQoL instruments and qualitative findings

Each table below presents the categories and subcategories pertaining to one of the Physical, Psychological, and Social domains. Beside each (sub)category, we listed the items from the 9 MS-specific HRQoL instruments that corresponded to the same construct, when present. In identifying relevant items, the subscale to which each item belonged was also considered, as a proxy for the intended contextual meaning and scope of the item. The comparison between the qualitative framework and the content of existing instruments (Tables 2–4) revealed heterogeneous coverage across domains.

**Table 2:** List of HRQoL Domains (all categories) in the Physical Domain, compared with the content of the items of the instruments included in the systematic review.

Physical Domain	Instrument (item number(s))
Symptoms (PwMS; HP)	FAMS (3, 5, 6, 8-14, 29-33, 48*, 53-59); FILMS (4^¶^, 6^¶^, 8, 12, 13, 17, 19^¶^, 20, 22, 24); HAQUAMS (3-7, 10-15, 16^¶^, 17^¶^, 18^¶^, 19^¶^, 20^¶^, 21, 22); MSIS-29 (1-11, 15^¶^, 20); MSQoL-54 (1*, 2* ^¶^, 3-11, 12^¶^, 21, 22^¶^, 23, 27, 29, 31, 32, 34-37, 46-50, 52); MusiQoL (1^¶^, 2^¶^, 3, 4, 5^¶^, 7, 8, 15, 16, 24*); PRIMUS Symptoms (1-6, 8-14, 17-19, 21, 22); RAYS Physical (1^¶^, 2-6, 7^¶^, 8-15); SQoL (1*, 2, 3*, 4, 9, 10)
• Mobility Impairment – Lower Limb (PwMS; HP)	MSIS-29 (3*, 5); FILMS (8*, 22*); FAMS (3, 5, 6); PRIMUS Activity Limitations (7-9, 10*, 11); HAQUAMS (3*, 11 –15); MusiQoL (1, 2*, 3, 4*); MSQoL-54 (3*, 4, 5*, 6 –11); RAYS Physical (1*^¶^, 3*, 4, 12*)
• Mobility Impairment – Upper Limb (PwMS)	HAQUAMS (16, 17^¶^, 18^¶^, 19^¶^); RAYS Physical (1*^¶^, 3*); PRIMUS Activity Limitations (10*); MSQoL-54 (3-4, 5*^¶^, 12*^¶^); MSIS-29 (1*, 2, 3*, 8*, 15^¶^)
• Visual Impairment (PwMS)	FILMS (17); HAQUAMS (3*, 10); MusiQoL (15); PRIMUS Symptoms (3, 6); RAYS Physical (10)
• Bowel and Bladder Incontinence (PwMS; HP)	FAMS (55*, 56*); MSIS-29 (20); FILMS (12); PRIMUS Symptoms (9, 10); HAQUAMS (3*, 21, 22)
• Swallowing Problems (HP)	HAQUAMS (20*^¶^); PRIMUS Symptoms (22)
• Balance and Coordination Impairment (PwMS; HP)	FILMS (8*); HAQUAMS (3*); MSIS-29 (4, 6); MusiQoL (4*); PRIMUS Symptoms (11); RAYS Physical (8, 9, 12)
• Fatigue/Fatigability (PwMS; HP)	FAMS (29 –33); FILMS (6^¶^, 11, 14, 19); HAQUAMS (3*, 6, 7); MSQoL-54 (23, 27, 29, 31, 32); MusiQoL (7, 8) PRIMUS Symptoms (S)
• Pain (PwMS; HP)	FAMS (9, 1213-14); FILMS (4^¶^, 13, 20, 24); HAQUAMS (3*, 4); MSQoL-54 (21, 22^¶^, 52); PRIMUS Symptoms (18, 21); RAYS Physical (6*); SQoL (4)
◦ Acute Pain (HP)	N.A.^
◦ Chronic Pain (PwMS; HP)	FILMS (4, 13, 20, 24); MSQoL-54 (21, 22, 52)
• Sexual Impairment (PwMS; HP)	MSQoL-54 (46 –49); PRIMUS Symptoms (12*^¶^)
• Asthenia/Strength Deficit (PwMS)	FAMS (11); MSIS-29 (2*); PRIMUS Symptoms (2)
ADL (PwMS; HP)	HAQUAMS (19^¶^, 20^¶^); MSQoL-54 (12^¶^); PRIMUS Activity Limitations (3-5, 12, 13); RAYS Physical (9*); SQoL (1*, 3*)
IADL (PwMS; HP)	FAMS (2, 6*); FILMS (4^¶^, 5*, 6^¶^, 11, 14, 19^¶^); HAQUAMS (12^¶^, 16^¶^, 17^¶^, 18^¶^); MSQoL-54 (4, 5, 13-16, 22^¶^); MSIS-29 (12*, 15^¶^, 16, 17); MusiQoL (1^¶^, 2^¶^, 5^¶^, 6); PRIMUS Activity Limitations (1, 2, 14, 15); RAYS Physical (1^¶^, 7^¶^, 9*); SQoL (1*, 3*)
• Difficulties in Mobility/Moving from One Place to Another (HP)	FAMS (6*); HAQUAMS (12*); MSIS-29 (17); MusiQoL (1*); RAYS Physical (1); PRIMUS Activity Limitations (15)
• Difficulties in Driving the Car (PwMS)	RAYS Physical (1)
Impact of the DMT on the Body (HP)	FAMS (45*^¶^); RAYS Additional Concerns (1*)

ADL, activity of daily living; DMT, disease-modifying therapy; FAMS, Functional Assessment of Multiple Sclerosis; FILMS, Functional Index for Living with Multiple Sclerosis; HAQUAMS, Hamburg Quality of Life questionnaire in Multiple Sclerosis; HP, health professional; IADL, instrumental activity of daily living; MSIS-29, Multiple Sclerosis Impact Scale; MSQoL-54, Multiple Sclerosis Quality of Life-54 items; MusiQoL, Multiple Sclerosis International Quality of Life questionnaire; N.A., not applicable; PwMS, people with multiple sclerosis; SQOL, Short Quality of Life scale.

* Indicates non-specific items, that is, items whose content is not specific enough to map precisely onto a (sub)category. For example, item “Does your health limit you in these activities? Lifting or carrying groceries” may not be considered specifically measuring the “Mobility Upper Limb” subcategory.

¶ Indicates items covering a facet of the construct that is in common between more than one (sub)category. These items are thus listed beside more than one (sub)category. For example, item “I have difficulties eating” may be considered specifically measuring the construct facet in common between the “Swallowing problems” and “Activity of Daily Living” categories.

^ Not specified as acute in the items listed in the more general “Pain” category.

**Table 3:** List of HRQoL Domains (all categories) in the Psychological Domain, compared with the content of the items of the instruments included in the systematic review.

Psychological Domain	Instrument (item number(s))
Cognitive Function (PwMS; HP)	FAMS (34 –37); FILMS (2, 7, 10, 15, 18); HAQUAMS (3*, 8, 9); MSIS-29 (27); MSQoL-54 (42 –45); MusiQoL (13, 14); PRIMUS Symptoms (7, 20); RAYS Psychological (6, 7, 9)
Health Perceptions (PwMS; HP)	FAMS (10, 50, 52^¶^); HAQUAMS (1, 2, 30^¶^, 31^¶^, 33^¶^); MSIS-29 (21*, 24^¶^); MSQoL-54 (2^¶^); PRIMUS QoL (11^¶^, 14^¶^, 15^¶^, 21^¶^, 22^¶^); RAYS Psychological (5^¶^); RAYS Additional Concerns (4); SQoL (5^¶^, 7^¶^)
• Illness as Frightening (PwMS; HP)	HAQUAMS (31^¶^); RAYS Psychological (5^¶^); SQoL (7^¶^)
• MS is Like a Sword of Damocles (PwMS; HP)	N.A.
• Aids Makes Me Feel Like my Health Has Worsened (HP)	N.A.
• Being Sick (PwMS; HP)	FAMS (10); MSIS-29 (21*)
◦ DMT Assumption (HP)	N.A.
◦ Asking for Help Due to MS Symptoms (PwMS)	N.A.
◦ Legal Disability Request (HP)	N.A.
• Being Fragile (PwMS)	SQoL (5^¶^)
Emotional Well-Being (PwMS; HP)	FAMS (15-17, 18, 19, 20, 21, 24, 26-28, 45*^¶^, 48, 51, 47^¶^, 52^¶^); FILMS (1, 9, 16^¶^, 21, 23); HAQUAMS (3*, 23, 30^¶^, 31^¶^, 32, 33^¶^, 34-36, 37*); MSIS-29 (24^¶^, 25 –29) MSQoL-54 (24-26, 28, 30); MusiQoL (9-12, 21^¶^, 22^¶^, 24*, 31*); PRIMUS QoL (4, 5, 7^¶^, 10, 11^¶^, 12, 14^¶^, 15^¶^, 16, 18^¶^, 21^¶^, 22^¶^); RAYS Psychological (1-4, 5^¶^, 11, 13, 15*); RAYS Additional Concerns (1*^¶^); SQoL (5^¶^, 6, 7^¶^, 8)
• The Emotional Cost of Exams/Therapy/Rehabilitation (HP)	N.A.
◦ DMT Impact (PwMS; HP)	FAMS (45*^¶^); MusiQoL (31*); RAYS Additional Concerns (1*^¶^)
• DMT Biological Impact (HP)	N.A.
◦ Time Dedicated to Exams/Therapy/Rehabilitation (HP)	N.A.
◦ Meeting People with a More Severe MS During Exams/Therapy/Rehabilitation (HP)	N.A.
• The emotional cost of MS symptoms and Functional Limitations (PwMS; HP)	FAMS (18, 19, 20, 21); FILMS (1); HAQUAMS (30^¶^, 31^¶^, 32, 33^¶^); MSIS-29 (24^¶^); MusiQoL (9 –12); PRIMUS QoL (10); RAYS Psychological (2-4, 5^¶^, 11, 13)
◦ The Emotional Cost of Cognitive Impairment (PwMS; HP)	N.A.^
◦ Anxiety Due to Bowel and Bladder Incontinence (PwMS; HP)	N.A.
◦ Fatigue/Fatigability: Impact on Social Relationships (HP)	N.A.
◦ Depressive Mood Due to Pain (PwMS)	N.A.
◦ The Emotional Cost of Sexual Impairment (PwMS; HP)	FAMS (48); HAQUAMS (23); MusiQoL (24); PRIMUS QoL (9*, 19); PRIMUS Symptoms (12*^¶^)
◦ Autonomy Loss (PwMS; HP)	RAYS Psychological (1)
• The Emotional Cost of Using Assistive Devices/Aids (PwMS; HP)	N.A.
• The Emotional Cost of MS Impact on Working Activities (PwMS)	N.A.
• Family Relationships (PwMS; HP)	FAMS (47^¶^); FILMS (9); MusiQoL (21^¶^, 22^¶^)
◦ Impact of MS on Getting Married (HP)	N.A.
◦ Impact of MS and Treatments on the Pregnancy Project (HP)	N.A.
◦ Fear of Genetically Transmitting the Illness to my Children (HP)	N.A.
◦ Fear of Not Being an Adequate Parent (PwMS; HP)	N.A.
• Authenticity/Inauthenticity (PwMS; HP)	N.A.
• Increased Sensitivity (PwMS)	HAQUAMS (3*)
MS is a Stressor (PwMS; HP)	FAMS (52^¶^); FILMS (16^¶^); HAQUAMS (31^¶^); MSIS-29 (24^¶^); MSQoL-54 (38 –41); MusiQoL (25, 26); PRIMUS QoL (11^¶^, 22^¶^); RAYS Psychological (5^¶^); SQoL (5^¶^, 7^¶^)

DMT, disease-modifying therapy; FAMS, Functional Assessment of Multiple Sclerosis; FILMS, Functional Index for Living with Multiple Sclerosis; HAQUAMS, Hamburg Quality of Life questionnaire in Multiple Sclerosis; HP, health professional; MSIS-29, Multiple Sclerosis Impact Scale; MSQoL-54, Multiple Sclerosis Quality of Life-54 items; MusiQoL, Multiple Sclerosis International Quality of Life questionnaire; N.A., not applicable; PwMS, people with multiple sclerosis; SQoL, Short Quality of Life scale.

* Indicates non-specific items, that is, items whose content is not specific enough to map precisely onto a (sub)category. For example, item “Have you lacked interest in sex?” may not be considered specifically measuring the “Emotional Cost of Sexual Impairment” subcategory.

¶ Indicates items covering a facet of the construct that is in common between more than one (sub)category. These items are thus listed beside more than one (sub)category. For example, item “I am losing hope about the fight against my illness” may be considered specifically measuring the construct facet in common between the “Health Perceptions” and “Emotional Well-Being” categories.

^ Not specified as referring to cognitive impairment in the items listed in the more general “The emotional cost of MS symptoms and Functional Limitations” category.

**Table 4:** List of HRQoL Domains (all categories) in the Social Domain, compared with the content of the items of the instruments included in the systematic review.

Social Domain	Instrument (item number(s))
Support (PwMS; HP)	FAMS (39, 40, 49); HAQUAMS (25, 26); MusiQoL (18, 19, 21^¶^, 22^¶^, 30); PRIMUS QoL (7^¶^); RAYS Social-familial (2*, 7*, 14); RAYS Additional Concerns (2)
• Limited Ability in Providing Support to Others (PwMS; HP)	RAYS Social-familial (2*, 7*); PRIMUS QoL (7^¶^)
◦ Due to MS Impairment (PwMS; HP)	N.A.
• Parenting (PwMS; HP)	RAYS Social-familial (7*)
• Other Relatives (HP)	N.A.
• Friends (HP)	N.A.
◦ Due to MS Psychological Burden (PwMS)	N.A.
• Limited Ability in Seeking Support (PwMS; HP)	N.A.
Social Connectedness (PwMS; HP)	FAMS (38, 42, 44, 47^¶^); HAQUAMS (24, 27^¶^, 28, 29); PRIMUS QoL (13^¶^); RAYS Additional Concerns (5^¶^); RAYS Social-familial (11)
• The Social Consequences of Invisible Symptoms (i.e., Fatigue/fatigability) (PwMS; HP)	N.A.
◦ The Social Consequences of Invisible Symptoms Being Misunderstood (PwMS; HP)	N.A.
• The Social Consequences of Bowel and Bladder Incontinence (PwMS; HP)	N.A.
• The Social Consequences of Time Dedicated to Exams/Therapy/Rehabilitation (HP)	N.A.
• The Social Consequences of Hiding the Diagnosis (HP)	N.A.
• The Impact of Illness Representation on Romantic Relationships (PwMS; HP)	N.A.
• The Impact of Sexuality Dysfunction on Intimate Relationships (HP)	PRIMUS QoL (9*^¶^)
Self-Perception in Social Settings (PwMS; HP)	MusiQoL (27, 28); PRIMUS QoL (6)
• Symptom-Related Stigma (HP)	N.A.
• Feeling Vulnerable (PwMS)	N.A.
• Perceiving Others’ Pity (PwMS)	N.A.
Social Participation (PwMS; HP)	FAMS (24*); FILMS (3*); HAQUAMS (27^¶^); MSQoL-54 (20, 33, 51); MusiQoL (5, 17, 20); PRIMUS QoL (1, 13^¶^, 17); RAYS Additional Concerns (5^¶^); RAYS Social-familial (3, 4, 6, 7*, 9, 13)
• Social Life (PwMS; HP)	MSQoL-54 (20*, 33*, 51*); PRIMUS QoL (1*, 13^¶^*, 17); RAYS Social-familial (4, 6, 9)
• Family Activities (PwMS; HP)	HAQUAMS (27^¶^); MSQoL-54 (20*, 33*, 51*); MusiQoL (20) PRIMUS QoL (1*, 13^¶^*); RAYS Additional Concerns (5^¶^); RAYS Social-familial (7*, 9)
• Hobbies and Leisure Time Activities (PwMS; HP)	FAMS (24*); FILMS (3*); MusiQoL (5); RAYS Social-familial (3, 13)
◦ Fear of the Disease Limits Travelling (PwMS)	N.A.
◦ Limitation in Playing Sports (PwMS)	N.A.
Work (PwMS; HP)	FAMS (22); FILMS (5, 11); RAYS Social-familial (1)
• Work Difficulties Due to MS Symptoms (PwMS; HP)	N.A.^
◦ Work Difficulties Due to Cognitive Impairments (PwMS; HP)	N.A.^
◦ Work Difficulties Due to Visual Impairments (PwMS)	N.A.^
◦ Work Difficulties Due to Invisible Symptoms (HP)	N.A.^
• Impact of Changes in Work Activities Due to MS (PwMS; HP)	N.A.
• Burden of Medical Leave Requirements (PwMS; HP)	N.A.
• Workplace Discrimination and Prejudice (PwMS)	N.A.
Finances (PwMS; HP)	N.A.
• Financial Impact of Disease Management (HP; PwMS)	N.A.
◦ Formal Caregiver Costs (PwMS)	N.A.
◦ Private Health Assistance Costs (PwMS; HP)	N.A.
◦ Driving License Renewal Costs (PwMS)	N.A.
◦ Formula Milk Costs (HP)	N.A.
• Financial Impact of Work-Related Issues (HP; PwMS)	N.A.
◦ Financial Risk of Freelancing (PwMS)	N.A.
◦ Risk of Job Loss or Demotion (HP)	N.A.

DMT, disease-modifying therapy; FAMS, Functional Assessment of Multiple Sclerosis; FILMS, Functional Index for Living with Multiple Sclerosis; HAQUAMS, Hamburg Quality of Life questionnaire in Multiple Sclerosis; HP, health professional; MSIS-29, Multiple Sclerosis Impact Scale; MSQoL-54, Multiple Sclerosis Quality of Life-54 items; MusiQoL, Multiple Sclerosis International Quality of Life questionnaire; N.A., not applicable; PwMS, people with multiple sclerosis; SQOL, Short Quality of Life scale.

* Indicates non-specific items, that is, items whose content is not specific enough to map precisely onto a (sub)category. For example, item “I took part in managing family and parental duties” may not be considered specifically measuring the “Social Participation” subcategory.

¶ Indicates items covering a facet of the construct that is in common between more than one (sub)category. These items are thus listed beside more than one (sub)category. For example, item “I spoke with my family/friends about my illness.” may be considered specifically measuring the construct facet in common between the “Support” and “Social Participation” categories.

^ Not specified in the items listed in the more general “Work” category.

#### Physical domain.

As detailed in Table 2, most instruments provided broad coverage of physical HRQoL, particularly regarding symptoms, mobility (lower and upper limb), fatigue, pain, and activity limitations. However, fine-grained distinctions emerging from the qualitative analysis—such as the separation between acute and chronic pain, or between lower- vs. upper-limb mobility impairments—were often not reflected in instrument items, which were frequently non-specific or mapped simultaneously to multiple subcategories. The category of Sexual Impairment was captured by only two instruments (MSQoL-54 and PRIMUS), and mainly at a general functional level, without addressing the broader experience described qualitatively. Overall, physical HRQoL constructs were represented, but item specificity remained limited.

#### Psychological domain.

Coverage in the Psychological Domain (Table 3) was substantial but uneven. Cognitive function, emotional well-being, and health perceptions were commonly represented across instruments. Nevertheless, several key psychological constructs derived from the qualitative study—including illness-as-threat, emotional responses to assistive devices, perceived fragility, and MS-related stressors—were either captured only indirectly or not addressed at all. Items overlapped across categories, especially for emotional well-being and health perceptions, reflecting limited conceptual differentiation. Some subcategories, such as the emotional impact of sexual impairment or disease-modifying therapies, were present but measured through generic or non-specific items. Overall, while core psychological constructs were included, granularity and contextualization were frequently lacking in the main MS-specific HRQOL questionnaires.

#### Social domain.

The most pronounced gaps emerged in the Social Domain (Table 4). Although several instruments partially covered support, social connectedness, social participation, and work-related issues, many subcategories identified qualitatively—such as limitations in providing or seeking support, the social consequences of invisible symptoms, stigma, romantic and intimate relationship challenges, and the financial impact of MS—were entirely absent from current instruments. When present, items typically reflected high-level constructs (e.g., general social participation), without addressing the specific mechanisms described by PwMS and HPs. Work- and finance-related categories were among the least represented. This domain therefore exhibited the greatest discrepancy between the qualitative findings and existing measurement tools.

## Discussion

This study investigated key determinants of HRQoL in PwMS by integrating a systematic review of MS-specific HRQoL instruments with qualitative data from online FGMs involving both PwMS and HPs. Combining these approaches provided a comprehensive view of MS as a multidimensional condition affecting physical, psychological, and social domains. Our findings confirm previously established HRQoL domains while identifying subdomains that reflect underrecognized aspects of the disease, highlighting the need for multidimensional, patient-centered assessment tools.

### Physical domain

Physical functioning–including mobility limitations, fatigue, pain, and sensory impairments—emerged as a central concern. The review emphasized capturing nuanced symptoms, such as balance disturbances, spasticity, and swallowing difficulties [62, 63], while FGMs highlighted mobility and pain as particularly impactful. Cognitive impairments further exacerbate functional limitations, restricting independence and daily task performance [64].

Current MS-specific HRQoL instruments capture mobility, fatigue, and pain but often lack specificity, failing to distinguish acute versus chronic pain or lower- versus upper-limb mobility. Sexual functioning, though important to PwMS and HPs, is inconsistently assessed [65]. Addressing these gaps could improve interpretability, sensitivity to change, and alignment with patient experiences, supporting targeted interventions and comprehensive monitoring of physical burden.

### Psychological domain

Cognitive dysfunction–including deficits in memory, attention, and executive function–was consistently reported as a major concern [66, 67]. Cognitive decline in MS can severely hinder daily functioning. Although it is increasingly recognized and routinely assessed in clinical practice, its broader consequences on work ability, autonomy, and social participation are often under addressed [67]. Emotional well-being, including depression, anxiety, stress, and the frustration related to disease unpredictability, was highlighted in both literature and FGMs [68, 69]. Participants emphasized relational strain linked to cognitive changes and uncertainty about disease progression.

While most instruments assess depression and anxiety, they rarely capture illness-related uncertainty, the emotional meaning of treatment, perceived fragility, or psychosocial effects of assistive device use. Similarly, nuanced emotional responses to cognitive decline remain underrepresented [69, 70].

### Social domain

Social isolation due to invisible symptoms, such as fatigue and cognitive impairment was frequently reported. Relationships with family and friends may become strained due to misunderstanding or lack of empathy [71], while social support can buffer negative effects [68, 2]. Work and financial challenges were also prominent: cognitive impairment, fatigue, and mobility issues limit employment, and insufficient accommodations or discrimination exacerbate difficulties [72, 73]. Costs of medications and healthcare further reduce quality of life [74].

Existing HRQoL instruments underrepresent critical social subdomains identified by PwMS, including stigma, consequences of invisible symptoms, challenges in seeking support, financial strain, and effects on intimate relationships [75, 76].

### Integrating implications across domains

Integrating HRQoL implications across domains shows that while current instruments capture key physical and psychological symptoms, they often fail to reflect more nuanced patient experiences, particularly in social and contextual areas. Underrepresented constructs—such as distinctions between acute and chronic physical symptoms, cognitive–emotional interactions, stigma, financial strain, and work-related challenges—are central to the lived experience of PwMS. In addition, our findings suggest that treatment burden—including the cognitive, emotional, and organizational demands associated with therapies, medical appointments, and disease monitoring—may act as a cross-cutting factor influencing HRQoL simultaneously across physical, psychological, and social domains. Incorporating these domains into a comprehensive item bank suitable for multidimensional CAT (MCAT) represents a promising direction for achieving more personalized, efficient, and accurate HRQoL assessment.

Multidimensional and adaptive assessment methods, including MCAT, offer clear advantages for monitoring complex constructs such as HRQoL in MS [22–25]. By drawing on the domains and subdomains identified in this study, these tools could enable continuous, patient-centered HRQoL tracking and facilitate shared decision-making in routine care.

A practical route for implementing this framework in clinical settings involves digital, modular ePRO (electronic patient-reported outcome) systems. These platforms can deliver brief, tailored assessments—potentially informed by MCAT principles—either remotely before appointments or during clinic visits. By selecting only the most informative items per domain, ePRO systems reduce patient burden while providing clinicians with automated scoring and concise, interpretable summaries [77, 78]. Although developing and calibrating an item bank requires an initial investment, such costs can be integrated within broader digital-health initiatives and are likely to be offset over time by gains in efficiency, reduced administration workload, fewer missing data, and streamlined documentation [79–81]. In addition to supporting future adaptive or item-bank–based developments, the conceptual framework generated here can also guide the systematic revision of existing MS-specific HRQoL questionnaires. Many instruments differ in domain structure, conceptual granularity, and coverage of patient-prioritized areas, leading to inconsistencies in measurement. Using the refined domains and subdomains identified in this study as a reference, existing instruments could be remodelled to enhance conceptual alignment, reduce redundancy, and incorporate underrepresented yet highly relevant aspects such as work participation, financial concerns, self-perception, and contextual influences. This represents a complementary and immediately feasible pathway for improving HRQoL measurement, particularly in settings where digital adaptive technologies are not yet available.

### Limitations

Several limitations should be noted. Both systematic review data and FGM insights rely on published literature and self-reported experiences, which may introduce reporting bias. FGM participants may have been more motivated or articulate, potentially overrepresenting certain viewpoints. Despite efforts to recruit a diverse sample, experiences from different cultural, socioeconomic, or geographic backgrounds may be underrepresented. Thus, transferability of our findings beyond the Italian healthcare system requires further investigation. Moreover, caregivers were not included, limiting insight into indirect or externally observed aspects of HRQoL. Including caregivers in future research would allow for a more comprehensive and multidimensional understanding of these aspects, enriching the interpretation of HRQoL through complementary perspectives and strengthening the overall validity of the findings. Nonetheless, the study provides a robust conceptual foundation for identifying HRQoL domains most relevant to PwMS and offers guidance for refining existing instruments or developing new patient-centered tools.

## Conclusion

By integrating systematic review evidence and qualitative FGM data, this study identified key HRQoL determinants in PwMS, and produced a refined framework encompassing Physical, Psychological, and Social domains with additional subdomains. Comparison with existing MS-specific instruments revealed uneven conceptual coverage, with psychosocial and role-related constructs often underrepresented.

These findings underscore the need for HRQoL tools that more comprehensively capture the lived experience of PwMS. The proposed domain structure can inform both the remodeling of existing questionnaires and the development of new item banks suitable for adaptive psychometric approaches such as MCAT, enabling efficient, patient-centered assessment. Future research should operationalize these domains into items, calibrate them using robust psychometric methods, and evaluate their applicability across diverse MS populations and clinical settings.

## Supporting information

S1 File Search strategy. (DOCX)

S2 File Focus Group Meeting guides. (DOCX)

S3 File Consolidated criteria for reporting qualitative studies (COREQ) Checklist. (DOC)

S4 File PRISMA flow diagram. (DOCX)

S5 File Mapping of original and final (sub)domain names from MS-specific questionnaires, as shown in Fig 1. (DOCX)

S6 File Audit trail. (DOCX)

## Abbreviations

ADL: activities of daily living
AISM: Italian Multiple Sclerosis Society
ASL: Azienda Sanitaria Locale
CAT: Computer-Adaptive Tests
COREQ: Consolidated Criteria for Reporting Qualitative Research
DMT: disease-modifying therapy
ECT: Engagement Coordination Team
EDSS: Expanded Disability Status Scale
FAMS: Functional Assessment of Multiple Sclerosis
FGMs: Focus group meetings
FILMS: Functional Index for Living with Multiple Sclerosis
FINCB: Fondazione IRCCS Istituto Neurologico Carlo Besta
FISM: the Scientific Research Area
HAQUAMS: Hamburg Quality of Life questionnaire in Multiple Sclerosis
HP: Health professional
HPs: Healthcare professionals
HRQoL: Health-related quality of life
IADL: Instrumental activities of daily living
MCAT: Multidimensional Computer Adaptive Testing
MS: Multiple sclerosis
MusiQoL: Multiple Sclerosis International Quality of Life questionnaire
PRIMUS: Patient Reported outcome indices for Multiple Sclerosis
PROs: Patient-reported outcomes
PwMS: People with MS
QoL: Quality of Life
SC: Steering committee
SQOL: Short Quality of Life scale
UNICA: ASL Cagliari/University of Cagliari
UNITO: University of Turin
