# Supplementary material for: Quality of life domains revised by people with multiple sclerosis and healthcare professionals for adaptive measure development
Source: PLoS One. 2026 Jun 11;21(6):e0349034. doi: 10.1371/journal.pone.0349034 (PMC13257964; doi:10.1371/journal.pone.0349034)
Supplement: S1 File — (DOCX) [file pone.0349034.s001.docx]

**S1 File: Search strategy**

**MEDLINE (Pubmed) (1946 to September, 2023)**

(((((("Quality of Life"[MeSH Terms] OR (("Life Quality"[Title/Abstract]) OR ("Health Related Quality Of Life"[tiab:~0]) OR ("Health- Related Quality Of Life"[tiab:~0]) OR ("HRQOL"[Title/Abstract] OR "HR-PRO"[Title/Abstract] OR "HRPRO"[Title/Abstract] OR "HRQL"[Title/Abstract] OR "HRQoL"[Title/Abstract] OR "QoL"[Title/Abstract] OR "quality of life"[Title/Abstract] OR "health profile*"[tiab:~0] OR "health status"[tiab:~0]))) AND ((((((((((((("Multiple Sclerosis"[Mesh:noexp] OR "Multiple Sclerosis, Chronic Progressive"[Mesh]) OR "Multiple Sclerosis, Relapsing-Remitting"[Mesh]) OR "Demyelinating Diseases"[Mesh:noexp]) OR "Optic Neuritis"[Mesh]) OR "Demyelinating Autoimmune Diseases, CNS"[Mesh:noexp]) OR "Encephalomyelitis, Acute Disseminated"[Mesh]) OR "Myelitis, Transverse"[Mesh]) OR ((((((((((((((((("multiple sclerosis"[Title/Abstract]) OR "chronic progressive multiple sclerosis"[Title/Abstract]) OR "progressive relapsing multiple sclerosis"[Title/Abstract]) OR "secondary progressive multiple sclerosis"[Title/Abstract]) OR "primary progressive multiple sclerosis"[Title/Abstract]) OR "relapsing remitting multiple sclerosis"[Title/Abstract]) OR "remitting-relapsing multiple sclerosis"[Title/Abstract]) OR "acute relapsing multiple sclerosis"[Title/Abstract]) OR "neuromyelitis optica"[Title/Abstract]) OR "optic neuritis"[Title/Abstract]) OR "devic disease"[Title/Abstract]) OR "demyelinating disease"[Title/Abstract]) OR adem[Title/Abstract]) OR "demyelinating disorder"[Title/Abstract]) OR "clinically isolated syndrome"[Title/Abstract]) OR "transverse myelitis"[Title/Abstract]) OR "acute disseminated encephalomyelitis"[Title/Abstract] OR ("encephalomyelitis"[Title/Abstract])))))))) AND ((((("Patient Reported Outcome Measures"[Mesh:NoExp]) OR "Patient Outcome Assessment"[Mesh])) OR (((patient[Title/Abstract] OR patients[Title/Abstract] OR patient's[Title/Abstract] OR self[Title/Abstract]) AND ((Outcome*[Title/Abstract] OR assessment*[Title/Abstract] OR rating*[Title/Abstract])) OR (report*[Title/Abstract] OR rate[Title/Abstract] OR rating*[Title/Abstract])))) NOT ("addresses’’[Publication Type] OR ‘‘biography’’[Publication Type] OR ‘‘case reports’’[Publication Type] OR ‘‘comment’’[Publication Type] OR ‘‘directory’’[Publication Type] OR ‘‘editorial’’[Publication Type] OR ‘‘festschrift’’[Publication Type] OR ‘‘interview’’[Publication Type] OR ‘‘lectures’’[Publication Type] OR ‘‘legal cases’’[Publication Type] OR ‘‘legislation’’[Publication Type] OR ‘‘letter’’[Publication Type] OR ‘‘news’’[Publication Type] OR ‘‘newspaper article’’[Publication Type] OR ‘‘patient education handout’’[Publication Type] OR ‘‘popular works’’[Publication Type] OR ‘‘congresses’’[Publication Type] OR ‘‘consensus development conference’’[Publication Type] OR ‘‘consensus developmentconference, nih’’[Publication Type] OR ‘‘practice guideline’’[Publication Type] NOT (‘‘animals’’[MeSH Terms]NOT ‘‘humans’’[MeSH Terms]))

**Embase (1974 to September, 2023)**

#1 'multiple sclerosis'/exp OR 'demyelinating disease'/exp OR 'optic neuritis'/exp OR 'acute disseminated encephalomyelitis'/exp OR 'multiple sclerosis' OR 'chronic progressive multiple sclerosis' OR 'progressive relapsing multiple sclerosis' OR 'secondary progressive multiple sclerosis' OR 'primary progressive multiple sclerosis' OR 'relapsing remitting multiple sclerosis' OR 'remitting-relapsing multiple sclerosis' OR 'acute relapsing multiple sclerosis' OR 'optic neurities' OR 'neuromyelitis optica' OR encephalomyelitis OR 'clinically isolated syndrome' OR 'transverse myelitis' OR 'devic disease' OR 'demyelinating disease' OR 'demyelinating disorder' OR 'acute disseminated encephalomyelitis' OR adem:ti,ab

#2 'quality of life'/exp/mj

#3 'life quality' OR 'health related quality of life' OR 'health- related quality of life' OR 'hr-pro' OR 'hrpro' OR 'hrql' OR 'hrqol' OR 'qol' OR 'quality of life' OR 'health profile*' OR 'health status':ti,ab

#4 #2 OR #3

#5 'patient-reported outcome'/exp OR 'outcome assessment'/exp

#6 ((patient OR patients OR patient* OR self) NEAR/4 (outcome* OR assessment* OR rating* OR report* OR rate OR rating*)):ti,ab

#7 #5 OR #6

#8 #1 AND #4 AND #7

#9 'addresses'/it OR 'biography'/it OR 'comment'/it OR 'directory'/it OR 'editorial'/it OR 'festschrift'/it OR 'interview'/it OR 'lectures'/it OR 'legislation'/it OR 'letter'/it OR 'news'/it OR 'congresses'/it

#10 'patient education handout' OR 'popular works' OR 'newspaper article' OR 'case reports' OR 'consensus development conference' OR 'consensus developmentconference, nih' OR 'practice guideline' OR 'legal cases':it

#11 'animal'/exp/mj

#12 'human'/exp/mj

#13 #11 OR #12

#14 'human'/exp/mj NOT 'animal'/exp/mj

#15 (#9 OR #10) NOT 'human'/exp/mj NOT 'animal'/exp/mj

#37 #8 NOT #15

**PsycInfo (1887 to September 2, 2023)**

S1 Autoimmune Diseases, CNS OR Optic Neuritis OR Encephalomyelitis, Acute Disseminated ) OR tiab("Multiple sclerosis" OR "Disseminated Sclerosis" OR "MS (Multiple Sclerosis)" OR "Multiple Sclerosis, Acute Fulminating" OR "Optic Neuritis" OR "Optic Neuritides" OR "devic disease" OR "Remitting-Relapsing Multiple Sclerosis" OR "Remitting Relapsing Multiple Sclerosis" OR "Relapsing-Remitting Multiple Sclerosis")

S2 mainsubject('quality of life') OR tiab('health related quality of life' OR 'health profile' OR 'health status' OR 'life quality' OR 'hr-pro' OR 'hrpro' OR 'hrql' OR 'hrqol' OR 'qol' OR 'quality of life')

S3 [mainsubject('patient-reported outcome') OR mainsubject('outcome assessment')](https://www.proquest.com/recentsearches.recentsearchtabview.recentsearchesgridview.scrolledrecentsearchlist.checkdbssearchlink:rerunsearch/5CEA6831F0FC4B0BPQ/None?site=psycinfo&t:ac=RecentSearches)

S4 tiab(patient OR patients OR patient* OR self) AND tiab(outcome* OR assessment* OR rating* OR report* OR rate OR rating*)

S5 title('addresses' OR 'biography' OR 'comment' OR 'directory' OR 'editorial' OR 'festschrift' OR 'interview' OR 'lectures' OR 'legislation' OR 'letter' OR 'news' OR 'congresses' OR 'patient education handout' OR 'popular works' OR 'newspaper article' OR 'case reports' OR 'consensus development conference' OR 'consensus developmentconference, nih' OR 'practice guideline' OR 'legal cases') NOT (mjsub(animal) NOT mjsub(human)))

S6 (S1 AND S2 OR (S3 AND S4) NOT S5

**Cumulative Index to Nursing and Allied Health Literature (CINAHL) (EBSCO host) (1981 – September 2, 2023);**

S1 ( MH Multiple Sclerosis OR MH Multiple Sclerosis, Relapsing-Remitting OR MH Demyelinating Autoimmune Diseases, CNS OR MH Optic Neuritis OR MH Encephalomyelitis, Acute Disseminated ) OR TIAB ( "Multiple sclerosis" OR "Disseminated Sclerosis" OR "MS (Multiple Sclerosis)" OR "Multiple Sclerosis, Acute Fulminating" OR "Optic Neuritis" OR "Optic Neuritides" OR "devic disease" OR "Remitting-Relapsing Multiple Sclerosis" OR "Remitting Relapsing Multiple Sclerosis" OR "Relapsing-Remitting Multiple Sclerosis")

S2 MH('quality of life') OR tiab('health related quality of life' OR 'health profile' OR 'health status' OR 'life quality' OR 'hr-pro' OR 'hrpro' OR 'hrql' OR 'hrqol' OR 'qol' OR 'quality of life')

S3 [mainsubject('patient-reported outcome') OR mainsubject('outcome assessment')](https://www.proquest.com/recentsearches.recentsearchtabview.recentsearchesgridview.scrolledrecentsearchlist.checkdbssearchlink:rerunsearch/5CEA6831F0FC4B0BPQ/None?site=psycinfo&t:ac=RecentSearches) OR tiab(patient OR patients OR patient* OR self) AND tiab(outcome* OR assessment* OR rating* OR report* OR rate OR rating*)

S4 title('addresses' OR 'biography' OR 'comment' OR 'directory' OR 'editorial' OR 'festschrift' OR 'interview' OR 'lectures' OR 'legislation' OR 'letter' OR 'news' OR 'congresses' OR 'patient education handout' OR 'popular works' OR 'newspaper article' OR 'case reports' OR 'consensus development conference' OR 'consensus developmentconference, nih' OR 'practice guideline' OR 'legal cases') NOT (mjsub(animal) NOT mjsub(human)))

S5 S1 AND S2 AND S3) NOT S4
