# Supplementary material for: Quality of life domains revised by people with multiple sclerosis and healthcare professionals for adaptive measure development
Source: PLoS One. 2026 Jun 11;21(6):e0349034. doi: 10.1371/journal.pone.0349034 (PMC13257964; doi:10.1371/journal.pone.0349034)
Supplement: S2 File — (DOCX) [file pone.0349034.s002.docx]

**S2 File**

**Focus Group Meeting Facilitator Guides**

**Focus Group Meeting Facilitator Guide for People with Multiple Sclerosis**

**Introduction**

Good morning everyone,

Thank you for agreeing to participate in the “INITIALISE” study, funded by FISM and coordinated by the University of Turin, with the involvement of the Neurological Institute Besta in Milan, the AISM rehabilitation center in Genoa, and the MS Center of the University of Cagliari.

As you may have been informed by your MS center representative and as you will have read in the information sheet, the study aims to improve how the quality of life related to health status is assessed in people with MS.

Health-related quality of life is defined as "the impact of health status, including the disease and its treatment, on the physical, psychological, and social domains of functioning and well-being" of that person.

To achieve this goal, we have planned two actions within the project:

1. A literature review was conducted to identify all items used to measure this construct in people with MS.
2. Based on these results, we decided to conduct focus groups with people with MS and professionals experienced in MS.

The objectives of today’s focus group are therefore twofold:

1. First, we want to identify the areas that contribute to your health-related quality of life.
2. Secondly, we want to verify that the areas identified in the literature are relevant to you, so that we only consider those that are truly important.

Your contribution is extremely valuable because it will allow us to highlight the aspects that are genuinely relevant (to health-related quality of life) for people with MS in the most truthful way possible. Together, we will build this knowledge. It will be a bit like putting together a puzzle, and you will provide us with each individual piece. Each piece of the puzzle will help us create a clearer and more defined picture of what truly matters for your quality of life.

As you may know, a focus group is a research technique that allows us to discuss a topic together and generate ideas. There are no right or wrong answers; your perspective is fundamental to us, and your opinion is what matters. It’s possible that some themes that emerge during our discussion may be personal and intimate. We understand that it might be difficult for some to share certain aspects of their experiences, and we are committed to creating an atmosphere of trust and collaboration. It is essential that you feel free to share everything that truly matters to you and your quality of life. We thank you in advance for the effort you will make.

My role (AMG) will be to guide the discussion and ensure that each of you has the opportunity to express your opinions. I ask for your permission in advance to interrupt or redirect attention to certain aspects when necessary. Dr. Di Domenico will assist me in moderating by taking written notes. It is very important for us not to miss any details of what is said. This is why we will record and transcribe the meeting. The audio recording will be deleted as soon as the transcription is complete.

**Introduction of the Facilitators (AMG and GDD)**

Do you have any questions or curiosities before we begin?

**STEP 1: Participant Introductions**

Great, we can start!

We would like to do a brief round of introductions for all participants, but to encourage some sharing right away, we want to start with a small prompt about the concept of quality of life. I ask you to listen to these words and begin to explore within yourselves what areas of life matter most to you.

We will present the following part in slides.

**[Adapted from SEIQOL-DW instructions]** For each of us, happiness and life satisfaction depend on those parts or areas of life that we consider important. When these areas are present and functioning well, we are generally happy, but when they are absent or not going well, we feel unhappy or concerned. In other words, these important areas determine the quality of our lives. What is considered important varies from person to person. What may be more important for one of you may not be for me or for your spouse/children/friends (mention one or more of these as appropriate) and vice versa.

We tend to realize the importance of certain aspects of our lives only when something intervenes and causes a change. Sometimes, it is easier to identify what is most important to us by trying to identify those areas of life that would cause (or cause) us significant concerns if they were to worsen or if we were to lose them entirely.

With all this in mind, would you like to introduce yourselves by stating your name and mentioning one or more areas of life that are important to you? For example, for me, the following are very important...

**STEP 2: What Determines Your Health-Related Quality of Life**

Now that each of us has started to feel what truly matters for our quality of life, we would like you to make an additional effort to shift from the concept of quality of life to that of health-related quality of life.

Present the following definition on the slide and show the diagram with the model of the three domains:

Health-related quality of life is defined as "the impact of health status, including the disease and its treatment, on the physical, psychological, and social domains of functioning and well-being" of that person.

**Note:** In the upcoming questions, we will use the term "disease" to refer generally to everything related to the experience of illness, including the therapy you take to prevent it or the care/rehabilitation you undergo to limit its impact.

**Impact of the Disease** Having MS is one of those life experiences that can have a significant impact on our quality of life, intervening and causing changes in those areas that truly matter to us.

- How does the disease (including the therapy you take to prevent it or the care/rehabilitation you undergo to limit its impact) impact what matters to you and therefore your quality of life?
- Thinking about what truly matters to you, in which relevant dimensions of your quality of life has the disease affected/does it affect or could it affect?
  - How does the disease impact your quality of life concerning your physical, psychological, or social functioning?
  - Can you provide an example for each of these domains (physical, psychological, and social)?

**Possible Additional Prompts by Directly Asking About the Three Macro Domains (Physical, Psychological, and Social)**

Present the slide with the three domains and their definitions (without labels).

- Does the disease limit your physical functioning and well-being? If so, how does this impact your quality of life? Can you provide an example?
- Does the disease interfere with your psychological well-being (for example, do you feel down, sad, or worried)? If so, does this impact your quality of life? Can you provide an example?
- Does the disease interfere with your social functioning (for example, social relationships, intimate relationships, friendships, work/study, other social activities, or going to public places like shopping, participating in community life, going to the post office, interacting with people outside your close circle)? If so, does this impact your quality of life? Can you provide an example?

**STEP 3: Check for Any Categories Not Elicited Independently by Participants**

Below we have prepared one or more questions for each label in the figure, to have everything ready at the time of facilitation. Naturally, questions related only to dimensions that did not emerge in the previous steps will be asked.

**Prompts for the Physical Domain**

**Symptoms of MS**

- Has the presence of symptoms, the emergence of new symptoms, or the increase in existing symptoms negatively impacted your quality of life? Can you provide an example?
- Would you say that the presence of symptoms, the emergence of new symptoms, or the increase in existing symptoms is an aspect through which the disease impacts/can impact your quality of life negatively? Can you provide an example?

**Brief Introduction to Distinguish ADLs and IADLs** Activities of daily living (ADLs) involve basic self-care activities. These include tasks like eating, dressing, bathing, moving (transferring from bed to chair, etc.), and using the bathroom. These activities are essential for a person's basic survival and well-being. Instrumental activities of daily living (IADLs), on the other hand, refer to more complex tasks that support independence and social participation. They include activities like managing finances, handling medications, shopping, preparing meals, cleaning the house, using the phone or other means of communication, and managing transportation. These activities are important for maintaining an independent and functional lifestyle.

**ADLs**

- Do difficulties with activities of daily living (e.g., bathing, using the bathroom, dressing, and eating) affect your quality of life? Can you provide an example?
- Has the presence or emergence of difficulties in activities of daily living (e.g., bathing, using the bathroom, dressing, and eating) or the increase in these difficulties negatively impacted your quality of life? Can you provide an example?
- Would you say that the emergence of difficulties in activities of daily living (e.g., bathing, using the bathroom, dressing, and eating) or the increase in these difficulties is an aspect through which the disease impacts/can impact your quality of life negatively? Can you provide an example?
- Are there important aspects of daily living that the disease (etc.) has impacted? If so, could you provide an example? Has this negatively impacted your quality of life?

**IADLs** **More Complex Activities Related to Social Activities**

- Do difficulties with instrumental/practical activities of daily living (e.g., managing finances, household chores, shopping, using the phone, and taking medications) affect your quality of life? Can you provide an example?
- Has the emergence of difficulties in instrumental/practical activities of daily living (e.g., managing finances, household chores, shopping, using the phone, and taking medications) or the increase in these difficulties negatively impacted your quality of life? Can you provide an example?
- Would you say that the emergence of difficulties in instrumental/practical activities of daily living (e.g., managing finances, household chores, shopping, using the phone, and taking medications) is an aspect through which the disease impacts/can impact your quality of life negatively? Can you provide an example?
- Are there important aspects of social functioning that the disease (etc.) has impacted? If so, could you provide an example? Has this negatively impacted your quality of life?

**Prompts for the Psychological Domain**

**Impact on Psychological Well-Being**

- Has the presence of symptoms, the emergence of new symptoms, or the increase in existing symptoms negatively impacted your psychological well-being? Can you provide an example?
- Do you think that psychological well-being is an aspect through which the disease impacts/can impact your quality of life negatively? Can you provide an example?
- Does living with the disease cause anxiety, sadness, or frustration? Can you provide an example?
- Are there important aspects of psychological well-being that the disease (etc.) has impacted? If so, could you provide an example? Has this negatively impacted your quality of life?

**Prompts for the Social Domain**

**Social Relationships**

- Have social relationships (e.g., family relationships, friendships) been impacted by the disease (etc.)? If so, could you provide an example? Has this negatively impacted your quality of life?
- Have difficulties with social relationships (e.g., family relationships, friendships) negatively impacted your quality of life? Can you provide an example?
- Does social isolation impact your quality of life? Can you provide an example?

**Work/Study**

- Has the disease (etc.) impacted your work/study life? If so, could you provide an example? Has this negatively impacted your quality of life?
- Do you feel that the disease has limited your ability to work or study? Can you provide an example?

**Final Steps**

**Conclusion of the Focus Group**

We will proceed to draw conclusions from our discussion. This step is important because it allows us to summarize what emerged, highlighting the aspects that have been the most significant for all of us. We will ask you for a moment of patience while we create a quick summary, which we will share with you to allow you to validate it before concluding.

- How did you feel about this discussion? Was there anything you particularly liked or didn't like?
- Would you like to add anything else?

**Thank You**

Thank you very much for your availability and for participating actively in this focus group. We are grateful for your contributions, which will help us achieve our research goals.

**Focus Group Facilitator Guide for Healthcare Professionals**

**Introduction**

Good morning everyone,

We would like to thank you for agreeing to take part in the "INITIALISE" study, funded by FISM and coordinated by the University of Turin, with the involvement of the Besta Neurological Institute in Milan, the AISM Rehabilitation Center in Genoa, and the MS Center at the University of Cagliari.

The study has the ambitious goal of improving the assessment of health-related quality of life (HRQoL) in individuals with multiple sclerosis (MS).

To achieve this objective, we have planned two key actions within the project:

1. A literature review has been conducted to identify all items used to date in measuring this construct in people with MS.
2. Based on these findings, we decided to conduct focus group meetings (FGMs) with people living with MS and MS experts.

Today's FGM has a twofold objective:

1. First, we aim to identify the aspects (referred to as domains) that contribute to the health-related quality of life in individuals with MS.
2. Second, we want to verify whether the domains identified in the literature are relevant to you as experts, in order to prioritize only those that are truly significant.

Therefore, today’s meeting enables us to reach an important goal: to understand the aspects you consider most relevant and to ensure that health-related quality of life is assessed in a way that resonates with both individuals living with MS and professionals working in this field.

We will build this knowledge together. Think of it as assembling a puzzle where each of you provides a piece. Each piece represents an insight into what truly matters for the quality of life of people with MS.

As you may know, a focus group is a research technique that allows for collective discussion and idea generation. There are no right or wrong answers; your perspective is crucial, and your opinions are what matter most.

My role (AMG) will be to guide the discussion and ensure that each of you has the opportunity to share your views. I may occasionally interrupt or refocus the discussion when necessary. Dr. Di Domenico will assist me in moderating, taking written notes.

It is essential that we do not miss any detail of what is shared today. For this reason, we will record and transcribe the session. The audio recording will be deleted once the transcription is complete.

**Facilitator introductions (AMG and GDD)**

Do you have any questions or concerns before we begin?

**STEP 1: Participant Introductions**

Great, we can begin!

Before starting the discussion, I would like to ask each of you to briefly introduce yourselves [name, profession, workplace].

**STEP 2: What Determines Health-Related Quality of Life in People with MS?**

Health-related quality of life (HRQoL) is defined as “the impact of health status, including illness and its treatment, on the physical, psychological, and social domains of functioning and well-being.”

Note: In the following questions, we will use the term "illness" to broadly refer to all aspects of the disease experience, including the therapies you take to prevent it and the treatments you undergo to mitigate its impact.

**Negative Impact of the Disease**

For each of us, happiness and life satisfaction depend on those aspects or areas of life that we consider important. When these areas of life are present and going well, we tend to be happy, but when they are absent or not going well, we may feel unhappy or concerned.
Living with MS is one of those life experiences that can have a significant impact on a person’s quality of life, affecting and altering the areas that truly matter to them.

- Keeping this in mind, how do you think the illness (including the therapies people take to prevent it, or the treatments/rehabilitation they undergo to mitigate its impact) affects what matters to them, and consequently, their quality of life?
  - How does the illness impact the quality of life of individuals in terms of their physical, psychological, or social functioning?
  - Could you provide an example for each of these domains (physical, psychological, and social)?

**Additional Prompts for Direct Questions on the Three Macro Domains**

**Presentation:** Please display the slide with the three domains and their definitions (without labels).

**Option 1:**

1. **Physical Domain:**
   - Does the illness limit the physical functioning and well-being of individuals affected by MS? If so, how does this impact their quality of life? Could you provide an example?
2. **Psychological Domain:**
   - Does the illness interfere with psychological well-being (for example, by worsening mood, leading to feelings of sadness and worry)? If so, how does this affect their quality of life? Could you provide an example?
3. **Social Domain:**
   - Does the illness impact social functioning (for instance, affecting social relationships, intimate connections, friendships, work/study, or other social activities that involve public places, such as grocery shopping, going to stores, participating in community life, visiting post offices, or interacting with people outside one’s close circle)? If so, how does this impact their quality of life? Could you provide an example?

**Option 2:**

1. **Impact on Physical Quality of Life:**
   - In what ways does MS deteriorate the quality of life for affected individuals through its impact on the physical domain (e.g., symptoms or limitations in daily activities such as managing finances, household chores, grocery shopping, using the phone, and taking medications)?
2. **Impact on Psychological Quality of Life:**
   - How does MS negatively affect the quality of life for individuals through its impact on the psychological domain (cognitive and/or emotional aspects)?
3. **Impact on Social Quality of Life:**
   - In what ways does MS worsen the quality of life for affected individuals through its impact on the social domain (for instance, affecting social relationships and intimate connections)?

**STEP 3: Check of All Categories Not Elicited by Participants**

Below, we have prepared one or more questions for each label in the figure to ensure comprehensive coverage during the discussion. These questions will be posed only for dimensions that did not emerge in previous steps.

**Prompts for the Physical Domain**

**Symptoms of MS**

- Has the presence of symptoms, the emergence of new symptoms, or the worsening of existing symptoms negatively impacted the quality of life for individuals with MS? Can you provide an example?
- Would you say that the presence, emergence, or worsening of symptoms is an aspect through which the disease negatively impacts the quality of life of individuals with MS? Can you provide an example?

**Activities of Daily Living (ADL)**

- Do difficulties in daily activities (e.g., bathing, using the bathroom, dressing, and eating) affect the quality of life for individuals with MS? Can you provide an example?
- Has the emergence or worsening of difficulties in daily activities (e.g., bathing, using the bathroom, dressing, and eating) had a negative impact on the quality of life for individuals with MS? Can you provide an example?
- Would you consider the emergence or worsening of difficulties in daily activities as an aspect through which the disease negatively impacts the quality of life of individuals with MS? Can you provide an example?

**Instrumental Activities of Daily Living (IADL)**

- Do difficulties in instrumental/practical daily activities (e.g., financial management, household chores, grocery shopping, using the phone, and taking medications) impact the quality of life for individuals with MS? Can you provide an example?
- Has the emergence or worsening of difficulties in instrumental/practical daily activities (e.g., financial management, household chores, grocery shopping, using the phone, and taking medications) had a negative impact on the quality of life for individuals with MS? Can you provide an example?
- Would you say that the emergence or worsening of difficulties in instrumental/practical daily activities negatively affects the quality of life of individuals with MS? Can you provide an example?

**Energy/Fatigue**

- Does the presence of fatigue/lack of energy (understood as a subjective sense of physical and/or mental exhaustion interfering with daily activities) impact the quality of life for individuals with MS? Can you provide an example?
- Has the emergence or worsening of fatigue/lack of energy had a negative impact on the quality of life for individuals with MS? Can you provide an example?
- Would you consider the presence or worsening of fatigue/lack of energy as an aspect through which the disease negatively impacts the quality of life of individuals with MS? Can you provide an example?

**Pain**

- Does the presence of pain (physical or psychological) impact the quality of life for individuals with MS? Can you provide an example?
- Has the emergence or worsening of pain (physical or psychological) had a negative impact on the quality of life for individuals with MS? Can you provide an example?
- Would you say that the presence or worsening of pain (physical or psychological) is an aspect through which the disease negatively impacts the quality of life of individuals with MS? Can you provide an example?

**Prompts for the Psychological Domain**

**Cognitive Functioning**

- Does the presence of cognitive deficits (which can involve multiple mental abilities, including learning, thinking, reasoning, memory, problem-solving, decision-making, and attention) impact the quality of life for individuals with MS? Can you provide an example?
- Has the emergence or worsening of cognitive deficits negatively impacted the quality of life for individuals with MS? Can you provide an example?
- Would you say that the presence or worsening of cognitive deficits is an aspect through which the disease negatively impacts the quality of life of individuals with MS? Can you provide an example?

**Perception of Health Status**

- Does the perception of one's health status—how individuals integrate objective information about their health with their feelings or evaluations of that information—affect the quality of life for individuals with MS? Can you provide an example?
- Has the perception of one's health status negatively impacted the quality of life for individuals with MS? Can you provide an example?
- Would you consider the way individuals with MS perceive their health status as an aspect through which the disease negatively impacts their quality of life? Can you provide an example?

**Emotional Well-Being**

- Does the disease negatively affect the emotional well-being (i.e., how good an individual feels in general and in their life) of individuals with MS? What aspects or experiences of the disease do you believe impact their emotional well-being negatively? Would you say this has an impact on their quality of life? Can you provide an example?

**Psychological Stress**

- In your opinion, is the experience of illness (as a stressor) a factor of stress (the emotional state of discomfort experienced by an individual in response to a specific stressor) for individuals with MS? What aspects of the illness trigger a stress response in individuals with MS? Would you say this has an impact on their quality of life? Can you provide an example?

**Prompts for the Psycho-Social Domain**

**Support**

- Do you think the experience of illness negatively impacts the ability of individuals with MS to seek and provide support to others? If so, do you believe this affects their quality of life? Can you provide an example?
- In general, do you think individuals with MS feel supported by their surroundings (family, friends, etc.) in coping with the illness? Do you believe that the response of their social environment to their new needs significantly impacts their quality of life? How? Can you provide an example?

**Self-Perception in Social Contexts**

- In your opinion, does having MS negatively impact how individuals perceive themselves in public places or in various social contexts (e.g., public transport, post offices, supermarkets)? If so, how does this affect their quality of life? Can you provide an example?
- Does the illness influence how individuals with MS feel in public spaces or social situations (e.g., using public transport, visiting post offices, going to the hospital, etc.)? If so, how does this affect their quality of life? Can you provide an example?

**Social Relationships**

- Do you think the disease negatively affects the quality of relationships individuals with MS have with others (partners, friends, family)? What aspects or experiences of the disease impact their social relationships negatively? If so, how does this affect their quality of life? Can you provide an example?
- Does the disease hinder the formation of new relationships?
- How has the disease impacted or hindered existing/new social relationships?

**Prompts for the Sexual Domain**

**Sexual Functioning and Satisfaction**

- Do you think the disease negatively affects sexual functioning or sexual satisfaction for individuals with MS? What aspects or experiences of the disease can negatively impact sexuality? Would you say this affects the quality of life for individuals with MS? Can you provide an example?

**STEP 4: Positive Impact (New Resources Emerged from the Experience of Illness)**

Sometimes individuals report that the experience of illness has not only been negative but has also allowed them to discover new aspects of themselves, make different choices, and overall improve their quality of life and well-being. In line with this, we ask:

- Are there aspects of the illness experience that you believe enhance the quality of life for those affected? If so, how?
- Through the experience of illness, are there things you have discovered to be important/useful for improving the quality of life related to health status in individuals with MS?
  - What aspects do you believe help individuals with MS promote a better quality of life in managing symptoms and physical limitations?
  - What aspects do you believe help individuals with MS promote a better quality of life considering the psychological impact of the illness?
  - What aspects do you believe help individuals with MS promote a better quality of life considering the neuropsychological impact (e.g., memory and/or attention problems) of the illness?
  - What aspects do you believe help individuals with MS promote a better quality of life considering the social functioning impact of the illness (e.g., social relationships, intimate relationships)?

**STEP 5: Summarizing What Has Been Discussed So Far**

Provide a summary of the contents that have emerged during the discussion.

Conclude with this question:

- Is there anything we haven’t asked that you feel is important to add regarding the impact of the illness (treatments or care) on the quality of life of individuals with MS?
