# Supplementary material for: Quality of life domains revised by people with multiple sclerosis and healthcare professionals for adaptive measure development
Source: PLoS One. 2026 Jun 11;21(6):e0349034. doi: 10.1371/journal.pone.0349034 (PMC13257964; doi:10.1371/journal.pone.0349034)
Supplement: S3 File — (DOC) [file pone.0349034.s003.doc]

**S3 File**

**Consolidated criteria for reporting qualitative studies (COREQ) checklist**

**Consolidated criteria for reporting qualitative studies (COREQ): 32-item checklist**

Developed from:

Tong A, Sainsbury P, Craig J. Consolidated criteria for reporting qualitative research (COREQ): a 32-item checklist for interviews and focus groups. *International Journal for Quality in Health Care*. 2007. Volume 19, Number 6: pp. 349 – 357

| **No. Item** | **Guide questions/description** | | **Reported on Page #** |
| --- | --- | --- | --- |
| **Domain 1: Research team and reﬂexivity** |  | |  |
| *Personal Characteristics* |  | |  |
| 1. Inter viewer/facilitator | Which author/s conducted the inter view or focus group? | | AMG (PhD, PsyD, expert in Qualitative Research and MS) and GDD (MSc). For more detains see paragraph “MATERIALS AND METHODS / Qualitative Study / FGM conduction” |
| 2. Credentials | What were the researcher’s credentials? E.g. PhD, MD | | AMG (PsyD; PhD), GDD (MSc) |
| 3. Occupation | What was their occupation at the time of the study? | | AMG: research fellow at UNITO, clinical psychologist at the MS Centre of the Research Institute that co-coordinate the study; GDD: research fellow at UNITO. |
| 4. Gender | Was the researcher male or female? | | Both females. |
| 5. Experience and training | What experience or training did the researcher have? | | AMG has previously participated in and coordinated qualitative studies running personal interview, focus group meeting ad nominal group technique meeting, and performing data analysis in several international projects.  GDD was trained in qualitative research for the purpose of the project |
| *Relationship with participants* |  | |  |
| 6. Relationship established | Was a relationship established prior to study commencement? | | Both facilitators were not acquainted to the interviewers prior to the interview. They both have already meet some HPs prior to the FGMs conduction. |
| 7. Participant knowledge of the interviewer | What did the participants know about the researcher? e.g. personal goals, reasons for doing the research | | Participants were informed of study aims and requirement and signed the written consent, in accordance with the Helsinki Declaration and EU Good Clinical Practice guidelines.”  See “Materials and methods / Qualitative Study / FGM conduction” where we reported that: *“The principal facilitator (AMG) began by explaining the purpose of the meeting and asking participants to introduce themselves. She then introduced each topic sequentially and guided the discussion.”* |
| 8. Interviewer characteristics | What characteristics were reported about the inter viewer/facilitator? e.g. Bias, assumptions, reasons and interests in the research topic | | Facilitators were member of the Qualitative Analysis Panel. AMG was specifically dedicated to focus group and she did not participate to any other study activities.  GDD participate in the literature review. |
| **Domain 2: study design** | |  |  |
| *Theoretical framework* | |  |  |
| 9. Methodological orientation and Theory | | What methodological orientation was stated to underpin the study? e.g. grounded theory, discourse analysis, ethnography, phenomenology, content analysis | See “Materials and methods / Qualitative Study / Analyses”  Content analysis. |
| *Participant selection* | |  |  |
| 10. Sampling | | How were participants selected? e.g. purposive, convenience, consecutive, snowball | See “Materials and methods / Qualitative Study / FGM Enrolment procedure” |
| 11. Method of approach | | How were participants approached? e.g. face-to-face, telephone, mail, email | See “Materials and methods / Qualitative Study / FGM Enrolment procedure” |
| 12. Sample size | | How many participants were in the study? | See “Results/ Qualitative results / FGMs participants and setting” |
| 13. Non-participation | | How many people refused to participate or dropped out? Reasons? | See “Results/ Qualitative results / FGMs participants and setting” |
| *Setting* | |  |  |
| 14. Setting of data collection | | Where was the data collected? e.g. home, clinic, workplace | See “Results/ Qualitative results / FGMs participants and setting”  The FGMs were run online via zoom |
| 15. Presence of non-participants | | Was anyone else present besides the participants and researchers? | No. |
| 16. Description of sample | | What are the important characteristics of the sample? e.g. demographic data, date | See “Results/ Qualitative results / FGMs participants and setting” and the S6 File – Audit trail |
| *Data collection* | |  |  |
| 17. Interview guide | | Were questions, prompts, guides provided by the authors? Was it pilot tested? | S2 File - Focus Group Facilitator Guides |
| 18. Repeat interviews | | Were repeat interviews carried out? If yes, how many? | No, we run FGMs |
| 19. Audio/visual recording | | Did the research use audio or visual recording to collect the data? | See “Materials and methods / Qualitative Study / FGM conduction”  *“The FGMs were audio-recorded and fully transcribed. Within two weeks of the FGMs, participants received a report summarizing the meeting for review (respondent validation).”* |
| 20. Field notes | | Were ﬁeld notes made during and/or after the inter view or focus group? | See “Materials and methods / Qualitative Study / FGM conduction”  “The FGMs were audio-recorded and fully transcribed. Within two weeks of the FGMs, participants received a report summarizing the meeting for review (respondent validation).” |
| 21. Duration | | What was the duration of the inter views or focus group? | See Results / FGMs participants and setting  *“Four online FGMs were conducted in April 2024, two with PwMS and two with HPs. The average duration of the FGMs were 144 minutes (SD 9.9) with PwMS, and 132 minutes (SD 14.1) with HPs.”* |
| 22. Data saturation | | Was data saturation discussed? | Yes. Data saturation was discussed during the analysis process. After conducting four focus group meetings, researchers observed recurring themes and minimal emergence of new concepts, suggesting that thematic saturation had been reached. The decision to stop data collection was based on the redundancy of the information and the consistency of themes across groups. |
| 23. Transcripts returned | | Were transcripts returned to participants for comment and/or correction? | See “Materials and methods / Qualitative Study / FGM conduction”  No, we did not returned transcript, but as reported in the manuscript: “The FGMs were audio-recorded and fully transcribed. Within two weeks of the FGMs, participants received a report summarizing the meeting for review (respondent validation).” |
| **Domain 3: analysis and ﬁndings** | |  |  |
| *Data analysis* | |  |  |
| 24. Number of data coders | | How many data coders coded the data? | Two coders: AMG and GDD. |
| 25. Description of the coding tree | | Did authors provide a description of the coding tree? | See “S6 File - Audit trail” |
| 26. Derivation of themes | | Were themes identiﬁed in advance or derived from the data? | See “Materials and methods / Qualitative Study / Analyses”  *“Content analysis was used to code the FGM transcripts, employing a combination of deductive and inductive approaches. The deductive process, informed by findings from Action 1 the literature review (existing HRQoL domains), applied category labels consistent with the literature when appropriate. Simultaneously, the inductive approach allowed for the identification of novel themes emerging enabled the identification of novel themes that emerged directly from the data. This dual approach began with a deductive framework to structure the initial analysis while incorporating inductive coding to capture insights not anticipated in the literature, ensuring a more comprehensive and nuanced understanding of the data.”* |
| 27. Software | | What software, if applicable, was used to manage the data? | We did not use any software. |
| 28. Participant checking | | Did participants provide feedback on the ﬁndings? | See “Materials and methods / Qualitative Study / FGM conduction”  *“The FGMs were audio-recorded and fully transcribed. Within two weeks of the FGMs, participants received a report summarizing the meeting for review (respondent validation).”* |
| *Reporting* | |  |  |
| 29. Quotations presented | | Were participant quotations presented to illustrate the themes/ﬁndings? Was each quotation identiﬁed? e.g. participant number | Yes, some of the most representative quotations are reported in the manuscript: FGMs results. All the others are reported in the “S2 File – Audit trail” |
| 30. Data and ﬁndings consistent | | Was there consistency between the data presented and the ﬁndings? | Yes. |
| 31. Clarity of major themes | | Were major themes clearly presented in the ﬁndings? | Yes. |
| 32. Clarity of minor themes | | Is there a description of diverse cases or discussion of minor themes? | “S6 File – Audit trail” includes a description of each category and subcategory. |
