# Supplementary material for: Quality of life domains revised by people with multiple sclerosis and healthcare professionals for adaptive measure development
Source: PLoS One. 2026 Jun 11;21(6):e0349034. doi: 10.1371/journal.pone.0349034 (PMC13257964; doi:10.1371/journal.pone.0349034)
Supplement: S4 File — (DOCX) [file pone.0349034.s004.docx]

**Identification of studies via databases and registers**

Records removed *before screening*:

Duplicate records removed via automation tools (n = 390)

Duplicate records removed manually (n = 95)

Records identified

(n = 5190):

MEDLINE (n = 3180)

Embase (n = 1822)

PsycInfo (n = 103)

CINAHL (n = 85)

**Identification**

Records excluded

(n = 4170)

Records screened

(n = 4705)

**Screening**

Records assessed for eligibility (n = 535)

Reports excluded:

Abstract only (n = 149)

Wrong population (n = 9)

Reviews (n= 27)

Not multi-dimensional HRQoL instruments (n = 42)

Studies included in review

(n = 308)

Related to 9 instruments*:

MSQoL-54 (n = 134)

MSIS-29 (n = 59)

FAMS (n = 49)

MusiQoL (n = 45)

HAQUAMS (n = 17)

PRIMUS (n = 7)

SQOL (n = 4)

RAYS (n = 3)

FILMS (n = 1)

**Included**

*Sum exceeds the total number of studies as some articles refer to more than one instrument.

*From:*  Page MJ, McKenzie JE, Bossuyt PM, Boutron I, Hoffmann TC, Mulrow CD, et al. The PRISMA 2020 statement: an updated guideline for reporting systematic reviews. BMJ 2021;372:n71. doi: 10.1136/bmj.n71

For more information, visit: <http://www.prisma-statement.org/>
