# Supplementary material for: Quality of life domains revised by people with multiple sclerosis and healthcare professionals for adaptive measure development
Source: PLoS One. 2026 Jun 11;21(6):e0349034. doi: 10.1371/journal.pone.0349034 (PMC13257964; doi:10.1371/journal.pone.0349034)
Supplement: S5 File — (DOCX) [file pone.0349034.s005.docx]

**S5 File.** Mapping of original and final (sub)domain names from MS-specific questionnaires, as shown in Figure 1.

| **MS-specific questionnaire** | **Original domain name** | **Final domain name(s) included in the figure** |
| --- | --- | --- |
| FAMS | Mobility | Mobility lower limb, Mobility upper limb |
|  | Symptoms | Symptoms |
|  | Emotional well-being | Emotional well-being |
|  | General contentment | Health perceptions, Emotional well-being, Distress |
|  | Thinking/fatigue | Energy/Fatigue |
|  | Family/social well-being | Support, Social relationships |
| HAQUAMS | Fatigue/thinking | Energy/Fatigue |
|  | Mobility lower limb | Mobility lower limb |
|  | Mobility upper limb | Mobility upper limb |
|  | Social function | Support, Social relationships, self-perception in social settings |
|  | Mood | Emotional well-being |
| MSQoL-54 | Physical function | Symptoms, ADL & IADL |
|  | Role limitations due to physical problems | ADL & IADL |
|  | Role limitations due to emotional problems | Emotional well-being |
|  | Bodily pain | Pain |
|  | Emotional wellbeing | Emotional well-being |
|  | Energy/fatigue | Energy/Fatigue |
|  | Health perceptions | Health perceptions |
|  | Cognitive function | Cognitive function |
|  | Health distress | Distress |
|  | Sexual function | Sexual function and satisfaction |
|  | Social function | Support, Social relationships, self-perception in social settings |
|  | Overall quality of life | Psychological domain |
| MSIS-29 | Physical | Physical domain |
|  | Psychological | Psychological domain |
| MusiQoL | ADL | ADL & IADL |
|  | Psychological well-being | Cognitive function, Health perceptions, Emotional well-being, Distress |
|  | Symptoms | Symptoms |
|  | Friends relationships | Social relationships, Support |
|  | Family relationships | Social relationships, Support |
|  | Sentimental and sexual life | Sexual function and satisfaction |
|  | Coping | Emotional well-being |
|  | Rejection | Support, Social relationships, self-perception in social settings |
|  | Satisfaction with health care | Health perception |
| PRIMUS | Symptoms | Symptoms |
|  | Activity limitations | ADL & IADL |
|  | QoL | Psychological domain, Social domain |
| RAYS | Physical | Physical domain |
|  | Psychological | Psychological domain |
|  | Social-familial | Support, Social relationships |
| SQoL | Physical | Physical domain |
|  | Mental | Psychological domain |
|  | Energy | Energy/Fatigue |
| FILMS | Fatigue | Energy/Fatigue |
|  | Bodily pain | Pain |
|  | Physical functioning | Physical domain |
|  | Emotional distress | Distress |
|  | Cognitive functioning | Cognitive function |

ADL, activity of daily living; FAMS, Functional Assessment of Multiple Sclerosis; FILMS, Functional Index for Living with Multiple Sclerosis; HAQUAMS, Hamburg Quality of Life questionnaire in Multiple Sclerosis; IADL, instrumental activity of daily living; MSQoL-54, Multiple Sclerosis Quality of Life-54 items; MSIS-29, Multiple Sclerosis Impact Scale; MusiQOL, Multiple Sclerosis International Quality of Life questionnaire; PRIMUS, Patient Reported outcome indices for Multiple Sclerosis; SQOL, Short Quality of Life scale.
