# Supplementary material for: Quality of life domains revised by people with multiple sclerosis and healthcare professionals for adaptive measure development
Source: PLoS One. 2026 Jun 11;21(6):e0349034. doi: 10.1371/journal.pone.0349034 (PMC13257964; doi:10.1371/journal.pone.0349034)
Supplement: S6 File — (DOCX) [file pone.0349034.s006.docx]

**S6 File**

**Audit trail FGMs INITIALISE**

# Open-Ended Questions Development

The FGM guides were developed capitalizing on the results of Action 1 (Literature review). The Qualitative Analysis Panel was in charge of devising the guides and defining questions and prompts, to maximise the opportunity to obtain high quality information from the different stakeholders. AMG and GDD developed the first draft, the other members of the Qualitative Analysis Panel provided suggestions and comment to refine the grids. The refined grids were discussed with the Engagement Coordination team (a group of expert PwMS) in a dedicated meeting and the final grid versions produced.

The decision to run FGMs instead of personal interview is because FGMs explore multiple perspectives simultaneously and promote interaction, brainstorming and elaboration of ideas. The Open-ended Questions are reported in the S2 File – FGM guides.

# Data Analysis

Content analysis was used to code the FGM transcripts, employing a combination of deductive and inductive approaches. The deductive process, informed by findings from Action 1 (existing HRQoL domains), applied category labels consistent with the literature when appropriate. Simultaneously, the inductive approach allowed for the identification of novel themes emerging directly from the data. This dual approach began with a deductive framework to structure the initial analysis while incorporating inductive coding to capture insights not anticipated in the literature, ensuring a more comprehensive and nuanced understanding of the data.

A line-by-line coding method ensured thorough and detailed analysis. A three-step coding scheme was implemented: Two researchers (AMG and GDD) analyzed the transcripts independently during steps 1 and 2, and collaboratively in step 3. In the first step, initial codes were extracted directly from participants' written responses. The second step involved data aggregation, with each researcher independently generating labels for codes and categories. In the third step, the researchers discussed and refined the independently generated codes and categories [Braun & Clarke. Qual Res Psychol. 2006;3(2):77-101. doi:10.1191/1478088706qp063oa]. The Consolidated Criteria for Reporting Qualitative Research (COREQ) [Tong A, et al. Int J Qual Health Care. 2007;19(6):349-57. doi:10.1093/intqhc/mzm042] guided the presentation of findings, leading to the creation of a detailed report. The adherence to the COREQ checklist is documented in the S3 File. The results from this phase were reviewed by the SC and the ECT, which determined the final set of HRQoL domains and updated the HRQoL list accordingly.

# Participants and setting

Four online focus group meetings (FGMs) were conducted in April 2025, two with PwMS and two with HPs. The average duration of the FGMs was 144 minutes (SD 9.9) with PwMS, and 132 minutes (SD 14.1) with HPs. Among those enrolled, five PwMS and two HPs were unable to participate due to medical or logistical reasons.

The PwMS sample included 14 participants (7 women, 7 men) with a median age of 52.5 years (range 36–64). EDSS scores ranged from 1.5 to 9.0 (median 4.0), and disease duration ranged from 5 months to 42 years (median 17). Half had a relapsing-remitting course and half a progressive form. Participants were recruited from Milano, Cagliari, and Genova, attending FGM 2 (n = 9) or FGM 3 (n = 5).

The HP sample comprised 18 professionals (15 women, 3 men) with a median age of 50 years (range 30–65) and 3–35 years of experience in MS care (mean 19.6). They had followed a median of 100 PwMS in the past six months (range 12–600) and were employed across MS centres, rehabilitation centres, and AISM facilities, representing roles such as neurologists, nurses, physiotherapists, psychologists, and social workers. HPs participated in FGM 1 (n = 10) and FGM 4 (n = 8).

# Detailed tables and full list of quotes

Qualitative data on HRQoL Domains were collected during the four FGMs and organized into three main theme: 1.1 Physical Domain; 1.2 Psychological Domain; 1.3 Social Domain.

Each theme consists of categories and subcategories (when applicable). For each theme, we provide a comprehensive summary table that includes all categories and subcategories. Additionally, we present one or more tables with quotes corresponding to each category and subcategory. This approach facilitates a detailed exploration of the qualitative data, enabling a nuanced analysis of participant perspectives within the identified themes. Quotes include the participants' IDs in brackets: (ID: number, 'P' or 'H'); where P = PwMS and H = Health Professional.

**Table S1** List of HRQoL Domains (only main categories)

| **Physical Domain** | **Psychological Domain** | **Social Domain** |
| --- | --- | --- |
| Symptoms (PwMS; HP) | Cognitive Function (PwMS; HP) | Support (PwMS; HP) |
| ADL (PwMS; HP) | Health Perceptions (PwMS; HP) | Social Connectedness (PwMS; HP) |
| IADL (PwMS; HP) | Emotional Well-Being (PwMS; HP) | Self-Perception in Social Settings (PwMS; HP) |
| Impact of the DMT on the Body (HP) | MS is a Stressor (PwMS; HP) | Social Participation (PwMS; HP) |
|  |  | Work (PwMS; HP) |
|  |  | Finances (PwMS; HP) |

**Table S1.1** List of HRQoL Domains (all categories) in the Physical Domain

| **Physical Domain** |
| --- |
| Symptoms (PwMS; HP) |
| - Mobility Lower Limb (PwMS; HP) |
| - Mobility Upper Limb (PwMS) |
| - Visual Impairment (PwMS) |
| - Bowel and Bladder Incontinence (PwMS; HP) |
| - Swallowing Problems (HP) |
| - Balance and Coordination (PwMS; HP) |
| - Fatigue/Fatigability (PwMS; HP) |
| - Pain (PwMS; HP) |
| - Acute Pain (HP) |
| - Chronic Pain (PwMS; HP) |
| - Sexual Function and Satisfaction (PwMS; HP) |
| - Asthenia/Strenght Deficit (PwMS) |
| ADL (PwMS; HP) |
| IADL (PwMS; HP) |
| - Mobility/Moving from One Place to Another (HP) |
| - Driving the Car (PwMS) |
| Impact of the DMT on the Body (HP) |

**Table S1.1.1** List of HRQoL Domains (all categories) in the Physical Domain with quotes

| **Physical Domain** | **Quotes** |
| --- | --- |
| Symptoms (PwMS; HP) | - "In recent years, it's become really tough because I've had neurological degeneration, which has physically thrown everything out of balance." (ID: 4P) |
| - Mobility Lower Limb (PwMS; HP) | - “The motor aspect is frequently regarded as the most significant impact and remains so for a substantial proportion of individuals." (ID: 11H) - "HP: It also made me think about the impact on a patient when they have to start using an aid [...]   Facilitator: How do you think it impacts them?  HP: [...] from the perspective of 'I have to coordinate myself to learn how to use the aid.'" (ID: 13H)   - "I mean, when it comes to using aids, there's a motor awkwardness [...]" (ID: 16H) - "The illness unfortunately makes itself felt, even though from the outside it's not so noticeable, because I have a cane. I walk, let's say, with a cane." (ID: 8P) - "At first, it was my leg giving out." (ID: 8P) - "I have a leg that bothers me, especially at rest." (ID: 1P) - "I move, I go up stairs differently, and that's... it's impacted that. [...] I can't walk like I used to." (ID: 1P) - "Clearly, the biggest physical impact for me has been the progression of the disease, leading to a decrease in my ability to walk, eventually reaching complete, let's say, immobility." (ID: 9P) - "So, I went from using a cane to crutches, to a walker at home, and now a wheelchair." (ID: 9P) - "I now have trouble walking, I limp, I walk with a cane, and I get really tired when I have to... if I need to walk a lot, I use two walking sticks, the Nordic walking poles." (ID: 2P) - “And the mobility issues are well-known, and let's say that this is one of those aspects that causes great suffering." (ID: 11P) - "Since March 2022, I started using a cane. Now I’ve moved on to crutches. First one, then two, and now I also use a walker, and at home, even a wheelchair." (ID: 14P) - "Because they spent their whole life expressing their full potential, and then suddenly, they find themselves with a significant disability, like balance problems or trouble walking." (ID: 7H) |
| - Mobility Upper Limb (PwMS) | - "My hands don’t grip the steering wheel well anymore." (ID: 9P) - "As of today, my main issues are increased weakness in my left arm." (ID: 7P) |
| - Visual Impairment (PwMS) | - "Physically, the disease has affected me... It started with optic neuritis, and I couldn’t see out of one eye. My vision has improved a bit, but it’s not the same as before. I have what they call a 'veil' over half my eye, like a little dome. That part just doesn’t see." (ID: 1P) - "In the past few years, my eye problems have gotten worse." (ID: 5P) |
| - Bowel and Bladder Incontinence (PwMS; HP) | - "And then there's also the sphincter-related issues because, let me tell you, it’s practically impossible to have MS with a series of brain lesions, maybe spinal lesions, and not end up with a neurogenic bladder." (ID: 17H) - "But what bothers me the most is the urinary urgency and the retention. I have to do self-catheterization twice a day, and that's a bit annoying. I've also had two infections." (ID: 8P) - "Even now I have limits, like [another participant’s name] if I’m not mistaken. I use an external catheter because unfortunately, I can’t go without it. It definitely affects me mentally too. At first, it was so bad I stopped going out because I couldn’t control the situation, and it was really embarrassing. I just didn’t go out anymore." (ID: 4P) - "The incontinence issues, I think we all deal with it... For at least ten years I’ve been using pads. I buy them in bulk because, unfortunately, I have to use them day and night." (ID: 5P) - "There’s also the issue of sexual function, which goes hand in hand with all the pelvic floor problems... It definitely has a huge impact." (ID: 4H) |
| - Swallowing Problems (HP) | - "Like dysphagia. These are the kind of hidden symptoms that rarely get discussed but can definitely have a big impact on quality of life. I’m specifically mentioning dysphagia because, in my opinion, it’s another aspect that we haven’t really talked about, but it certainly has a significant impact as well." (ID: 11H) |
| - Balance and Coordination (PwMS; HP) | - "But compared to other symptoms, like ID: 17H was saying, fatigue or balance issues come to mind, I think it's less relevant." (ID: 13H) - "My main problems have been with balance." (ID: 5P) - "And where the limitations, in many phases, are fairly modest... in my opinion, fairly modest. It doesn't mean they're gone, but maybe 'I can't run anymore, but I have to be careful when I take the bus because I have some balance issues.'" (ID: 8H) - "Because they lived their whole life expressing their full potential, then suddenly they're faced with significant disability, maybe with balance problems or trouble walking." (ID: 7H) |
| - Fatigue/Fatigability (PwMS; HP) | - "There can also be an indirect cascade effect in all these areas related to fatigue. Moving around to get to a hospital, reaching a rehabilitation ward, doing motor or cognitive rehabilitation, these are all things that tire the person out, and sometimes it's not seen as positive." (ID: 7H) - "But compared to other symptoms, like the colleague was saying, fatigue or balance issues come to mind, I think it's less relevant." (ID: 13H) - "Often, they're overwhelmed with rehab appointments... This really affects their social life, as well as their overall fatigue. I heard just yesterday about someone who finished a 45-minute water therapy session and was completely wiped out. Exhausted but happy, because they know it’s an effective treatment for them. But then they said, 'I get home and can't wait to lie down on the couch because I need to recover from this effort and therapy.'" (ID: 16H) - "Honestly, when it comes to the impact on life, social activities, and work, fatigue—or exhaustion—is probably the most important factor." (ID: 17H) - "Another thing I struggle with is fatigue. I’ve been told it’s a common issue, so I guess it’s not just me. There are times when my energy is completely drained, like my phone’s battery warning me it's almost dead. I get stuck. No matter where I am, I can't think or do anything; I feel totally wiped out." (ID: 1P) - "So, there’s definitely been a decline in my physical quality of life. Not to mention the fatigue, which has already been brought up." (ID: 9P) - "Fatigue can be incredibly disabling. Sometimes it feels like such a burden to do simple things, like washing the floor, which should only take five minutes but feels overwhelming." (ID: 7P) - "I also deal with fatigue, and it’s increasingly limiting. I only manage to do one thing a day. If I have therapy, that’s the only thing I do. If I need to shop, that’s it for the day, and I have to..." (ID: 6P) - "It’s this enormous fatigue that comes back every day. It’s like moving a rock just a little bit each day, and the next day you’re right back at it, needing to move it again, with all the effort and commitment just to keep from getting worse." (ID: 11P) - "Fatigue is a big issue." (ID: 12P) - "This fatigue that hits every morning, and you still have to get things done despite it." (ID: 11P) - "In many cases, the limitations are quite mild and... I think they're fairly mild. That doesn’t mean there aren't any, but maybe I just get more tired than before." (ID: 8H) - "Even if you don’t have any major physical problems, the fatigue is a major symptom. We were talking about invisible symptoms earlier, and fatigue really impacts every level of the condition." (ID: 2H) |
| - Pain (PwMS; HP) | - "Physical pain is often underestimated, even though we know there's neuropathic pain, which can be central in origin and part of the MS symptom panel." (ID: 16H) - "Pain, I think, is an important question because it tends to be one of those symptoms that's not often seen or well understood." (ID: 11H) - "Pain is often very disabling for people with MS. There are many examples of people who struggle significantly with pain, particularly neuropathic pain, which is one of the hardest types to treat and the most severe. From a quality of life perspective, someone with pain definitely experiences a much lower quality of life compared to someone without pain. So, this is definitely an important factor." (ID: 11H) - "I agree with everyone. The trigeminal neuralgia, for example, has had a significant impact on many of my patients. It really makes a big difference." (ID: 7H) - "Yes, absolutely. I do physical therapy from Monday to Saturday. Currently, I'm doing it four times a week, which is crucial for me—especially for my back, the fluid retention from sitting too long, and the pain, mainly in my back. I spend most of my time sitting, and the physical efforts of moving from the wheelchair to the ground or to a chair are significant, often requiring me to take anti-inflammatories." (ID: 9P) - "The latest relapse was different from usual and showed up as pain while walking and under my foot. It opened my eyes to a new aspect of the disease: beyond fatigue and paresthesias, MS can also cause pain." (ID: 12P) - "Neuropathic pain isn't very common, but when it occurs, it has a huge impact. The patient can't think about anything else. I’ve had a few cases of severe neuropathic pain that really pervades a person's existence. It's very hard to manage, even with strong medications like Fentanyl, which is used for cancer pain, including terminal stages. So, when it does happen, even if not very often, it is extremely impactful." (ID: 4H) |
| - - Acute Pain (HP) | - "In my opinion, this is definitely a symptom with a significant impact, especially acute pain—like trigeminal pain, but also spasms and pain in the lower limbs. It’s not always present in advanced stages; it can also show up in intermediate stages of the disease." (ID: 11H) |
| - - Chronic Pain (HP) | - "Often, even during rehabilitation treatments, you might notice a grimace and ask, 'Are you in pain? Is it bothering you?' The response might be, 'It’s bothering me.' This seems like a form of chronic discomfort—a persistent feeling that something isn't quite right with the body. However, it’s not categorized as pain anymore. Instead, it’s described as a type of discomfort that still affects daily life and may be limiting, but it isn't considered pain in the traditional sense. This could be because it’s not sharp; it’s often a dull ache that can’t be pinpointed to a specific area of the body."(ID: 14H) |
| - Sexual Function and Satisfaction (PwMS; HP) | - “The drop in testosterone in our condition is symptomatic.” (ID: 2P) - PwMS 1: [Sexuality] is limited by mobility issues; that’s always the issue. Just like you can't take a tram, you can't do fancy things at certain moments. But undoubtedly… it’s limited by movement problems, that’s it.” (ID: 11P)   PwMS 2: And definitely energy too, finding the right moment [they laugh]. (ID: 12P)  PwMS 1: You need to find the right alignment.” (ID: 11P)   - “Unfortunately, for example, I’ve had problems with erections for a long time. At first, they were somewhat manageable, then barely manageable. Now I need injections, otherwise, nothing works, for example. And, well, it limits you a lot, even though everything works fine since I have had a child. So, okay, but it still limits you a lot, the fatigue… thinking about how to manage it, how to give pleasure, how… Even to your partner, right? It’s not easy.” (ID: 14P) - “But sometimes it happens that, especially in older men, it is generally assumed that sexual life is no longer relevant, as if it were an abandoned thought. I’m not talking about people in their nineties, but also those in their fifties or sixties. Younger people have a lot of shame because sexual issues… affect the couple, so it’s more about self-image. And about one’s role within the couple. If the partner helps you, whether it's with washing or catheterization, that whole sphere is put aside. It’s left there. So, the impact is definitely significant.” (ID: 2H) - “The issue of sexual function in tandem with all the pelvic floor disorders. But it seemed like such a huge and complex issue to tackle from multiple perspectives, depending on the stages of the disease, depending on age, so I thought 'maybe it’s not the right time.' But it actually has a huge impact.” (ID: 4H) |
| - Asthenia/Strength Deficit (PwMS) | - “The hands no longer grip the steering wheel properly.” (ID: 9P) |
| ADL (PwMS; HP) | - “I: The implication of all these interventions (please correct me if I’m wrong) is that: one way the disease affects quality of life is by creating difficulties in daily activities, both basic and more complex ones.  HP: Unfortunately, absolutely yes.” (ID: 11H) - “I move differently, I handle stairs differently, and this is… [MS] has impacted this.” (ID: 1P) - “I had to give up driving because, as mentioned, my hands no longer grip the steering wheel properly. So, with the progression of the disease, there has also been a gradual decrease in my autonomy. The thing that impacts me the most psychologically is the dependence on others. I’ve always been a person…” (ID: 9P) - “I live with a 24-hour caregiver, which is essential. So, I’ve had to come to terms with depending on someone else, even for basic things like washing myself.” (ID: 9P) - “For example, the last trip I took, which was years ago, involved dealing with a wheelchair to be loaded onto the plane, showering in the hotel, and I did it without problems with a friend who knew very well… who literally helped me wash.” (ID: 9P) - “I have difficulty with stairs, both going up and down, and I need support. If I see a staircase in front of me that I need to climb, I have trouble, and the same goes for going down.” (ID: 5P) - “I now have difficulty walking, I limp, I use a cane, and I get very tired when I have to walk a lot. I use two walking sticks, the Nordic walking poles. But I bought a cane that turns into a seat… into a small chair. When I go for a walk, I get tired after… a certain amount of time. If I’m with someone, they know; I stop, open the seat, sit for a couple of minutes, five minutes, ten minutes.” (ID: 2P) - “Not long trips, but traveling from one neighbourhood to another. The mobility problems are well known, and this is one of those aspects that cause significant suffering.” (ID: 11P) |
| IADL (PwMS; HP) | - “I: The implication of all these interventions (correct me if I’m wrong) is that one way the disease affects quality of life is by creating difficulties in daily activities, both basic and more complex ones. HP: Unfortunately, absolutely yes.” (ID: 11H) - “I had to give up driving because my hands no longer grip the steering wheel properly. So, as the disease progressed, there was a gradual loss of autonomy. What affects me the most psychologically is the dependence on others. I’ve always been a person…” (ID: 9P) - “But I hold on to the little bits of autonomy I still have, like working in front of a screen—I can still type a bit with one hand on the keyboard.” (ID: 9P) - “The fatigue, which can sometimes be particularly disabling, makes even something like cleaning the floor—tasks that only take five minutes and are so simple—feel heavy.” (ID: 7P) - “I experience the effects of fatigue too, and it’s becoming more and more limiting. I limit myself to one task a day: if I have therapy, that’s all I do; if I have to go shopping, that’s all I do, and I have to…” (ID: 6P) - “It’s undeniable that there are many things I can’t do. […] The limitations are there, though.” (ID: 13P) - “You feel insecure, maybe about facing certain situations, or sometimes, I don’t know, like miscalculating the groceries—things like that, you know? Those immediate tasks that require quick thinking. It can be frustrating at times because you think, ‘Why did I come to such a silly conclusion? Why did I make this mistake?’ It weighs on you a little.” (ID: 10P) |
| - Mobility/Moving from one Place to Another (HP) | - “In reality, you live in denial (of sphincter symptoms) until you reach the point where you have to go to the bathroom every half hour or every hour. The result is that either you know all the cafes along your routes in the city, or you don’t go out anymore. As was mentioned, you stop going out because you can’t engage in those activities. That, too, is a taboo.” (ID: 17H) - “And mobility issues are well-known, and let’s say this is one of those aspects that cause great suffering. Because every day, you have to fight against these architectural barriers.” (ID: 11P) - “Speaking from my own experience, I’m in a wheelchair, and for many things… public transport is just not an option, not even as a joke.” (ID: 13P) - “Time management changes for many people. Meaning, ‘if I can’t run to catch the bus, I need to adjust the time I take to get to work. Because if I have to go slowly and miss the bus, I need to leave home much earlier…’ and everything else. And don’t even get me started if I have to use an aid like a wheelchair and can’t use a car, for example, in a city like Milan. Simply put… ‘I’m never sure if the stairlift will work,’ and in any case, everything takes much longer.” (ID: 8H) - “So, it’s about a daily life that has enormous challenges: transportation, actually being able to visit your daughter or grandson, wherever they may be.” (ID: 8H) |
| - Driving the Car (PwMS) | - “The impact is twofold: one is that I no longer trust myself to drive at night, even though I still have my license. I know very well that I don't see well with this eye, so I don’t drive at night.” (ID: 1P) - “Unfortunately, I haven't driven for ten years. I just don’t feel able to drive anymore, and this is quite limiting because I have to ask others for help with everything. I have to get my husband to pick me up or drive me around. My eye problems have increased in recent years.” (ID: 5P) - “Sometimes I use public transport, but I have difficulties there too because I struggle to get on and off.” (ID: 5P) |
| Impact of the DMT on the Body (HP) | - “Often, the medications used for pain management can cause discomfort and lead to motor issues. I believe this has a significant impact.” (ID: 14H) - “I could never administer the interferon injections myself; my husband had to do it. The main issue in recent years was that it became overwhelming. I was using areas like the inner arms, buttocks, and abdomen. Even now, years after stopping, I still have indentations in my arms because I could no longer tolerate that treatment, which is why I switched to tablets.” (ID: 5P) - “The type of medication significantly affects daily life. Taking a tablet impacts diet, influencing meals and various aspects, such as elimination. Some medications have both physical and psychological effects. I believe that the conditions caused by treatment are a top priority. Patients care deeply about their therapy, as it is crucial for their future, so the treatment is essential and influences many aspects of life.” (ID: 6H) |

**Table S1.2** List of HRQoL Domains (all categories) in the Psychological Domain

| **Psychological Domain** |
| --- |
| Cognitive Function (PwMS; HP) |
| Health Perceptions (PwMS; HP) |
| - Illness as Frightening (PwMS; HP) |
| - MS is Like a Sword of Damocles (PwMS; HP) |
| - Aids Makes Me Feel Like my Health Has Worsened (HP) |
| - Being Sick (PwMS; HP) |
| - - DMT Assumption(HP) |
| - - Asking for Help Due to MS Symptoms (PwMS) |
| - - Legal Disability Request (HP) |
| - Being Fragile (PwMS) |
| Emotional Well-Being (PwMS; HP) |
| - The Emotional Cost of Exams/Therapy/Rehabilitation (HP) |
| - - DMT Impact (PwMS; HP) |
| - - - DMT Biological Impact (HP) |
| - - Time Dedicated to Exams/Therapy/Rehabilitation (HP) |
| - - Meeting People with a More Severe MS During Exams/Therapy/Rehabilitation (HP) |
| - The emotional cost of MS symptoms and Functional Limitations (PwMS; HP) |
| - - The Emotional Cost of Cognitive Impairment (PwMS; HP) |
| - - Anxiety Due to Bowel and Bladder Incontinence (PwMS; HP) |
| - - Fatigue/Fatigability: Impact on Social Relationships (HP) |
| - - Depressive Mood Due to Pain (PwMS) |
| - - Sexual Function and Satisfaction (PwMS; HP) |
| - - Autonomy Loss (PwMS; HP) |
| - The Emotional Cost of Using Assistive Devices/Aids (PwMS; HP) |
| - The Emotional Cost of MS Impact on Working Activities (PwMS) |
| - Family Relationships (PwMS; HP) |
| - - Impact of MS on Getting Married (HP) |
| - - Impact of MS and Treatments on the Pregnancy Project (HP) |
| - - Fear of Genetically Transmitting the Illness to my Children (HP) |
| - - Fear of Not Being an Adequate Parent (PwMS; HP) |
| - Authenticity/Inauthenticity (PwMS; HP) |
| - Increased Sensitivity (PwMS) |
| MS is a Stressor (PwMS; HP) |

**Table S1.2.1** List of HRQoL Domains (all categories) in the Psychological Domain with quotes

| **Psychological Domain** | Quotes |
| --- | --- |
| Cognitive Function (PwMS; HP) | - “The problem is that we know from extensive literature that cognitive involvement is almost inevitable.” (ID: 17H) - “Other than some concentration issues, the illness doesn’t really cause me major disturbances.” (ID: 7P) - “Sometimes, my memory deficits lead me to forget things I’d like to remember—like trips I’ve taken. I tend to block out almost everything from those trips because my mind can’t hold it all. I have to make room for new memories, work-related things, and so on, so I have no choice but to set the others aside.” (ID: 7P) - “I’ve noticed that I also struggle with concentration, and that’s likely affected my work as well.” (ID: 6P) - "Mentally, I feel much slower, even in terms of attention. That, for me, is what the disease has affected the most, since physically it has, let’s say, spared me. But mentally, in terms of sharpness, I can feel it. You know, you just feel slower in your thinking and reasoning, even if it doesn’t seem that way." (ID: 10P) - “The cognitive aspect is very important.” (ID: 8H) - “Sometimes, even when physical limitations are relatively minor, the person might struggle with concentration, and that can have a major impact” (ID: 8H). |
| Health Perceptions (PwMS; HP) |  |
| - Illness as Frightening (PwMS; HP) | - “The impact is quite profound, as we know, because the term ‘multiple sclerosis’ is recognized even outside the medical field. It is one of those conditions that elicits fear and is frequently mentioned in news reports, alongside other incurable diseases. […] Thus, it is grouped with other major fears, such as cancer. Consequently, when a diagnosis is made, it signifies that ‘from this moment on, my life will be permanently different, and I am, in effect, among the most unfortunate people in the world” (ID: 17H). - “The problem is that patients and those who have received a diagnosis have their own lives, while some people have a much more limited and almost exclusively dramatic perspective. This creates the first impact, I think—it's all about the perspectives. A patient might be doing well after experiencing an optic neuritis that has resolved, or something similar. In my opinion, this is where, during the diagnostic phase, much of the discussion about the psychological impact comes into play, namely, the feeling that ‘my potential is severely stifled.’” (ID: 17H) - "Not knowing much about the disease, I immediately thought of a diagnosis leading to a wheelchair. And that was difficult to shake off as an impact, let's say." (ID: 4P) - “When I was diagnosed, I also imagined myself in a wheelchair the very next day.” (ID: 7P) - “Unfortunately, people don’t really understand what this illness entails in certain situations. They don’t tell you; you discover it through experience. So… this can be frightening because, if I think about where I might be in twenty years, I probably envision myself in a bed.” (ID: 11P) |
| - MS is Like a Sword of Damocles (PwMS; HP) | - “The illness is always like a sword of Damocles hanging over your head. You can’t forget about it, but really, even a healthy person could face something unexpected one day. We might have a slightly higher chance of something happening, but we never know how far it will go.” (ID: 7P) - “In the early stages, the patient has many questions. They don’t really know where they’re headed, so there’s a phase of confusion where they’re unsure about how the illness will impact their family life and work life. When we use clinical assessment scales and questionnaires, particularly regarding mobility, the question is, ‘Does the condition impact your walking?’ A patient in the early stages, who may not have mobility issues, might say they’re not having problems now but express concern, saying, ‘I might develop an issue.’ So, it’s not just about having a problem at the start; it’s about worrying about what might happen later.” (ID: 13H) - “MS is a source of stress because it’s not defined. You don’t know what today or a year from now will look like. You can read everything and hear everything, but no one can tell you how it will evolve. It’s true that no one knows how their life will evolve, but they’re telling you that you have something that will change over time. Facilitator: The unpredictability of the illness. PwMS: Exactly, the unpredictability.” (ID: 1P) - “PwMS: Having multiple sclerosis feels like living under a sword of Damocles. It’s always there, pointed at you, and you never know when it will fall. The harsh reality of the illness is this. It also depends on the individual—how they face challenges and react. It varies a lot.” (ID: 10P) - “I also follow many patients who are physically doing great—some even run ultramarathons—but psychologically they may struggle much more than those who are already in wheelchairs. This relates to a shift in perspective; at some point, the person feels disoriented, loses their reference points, and doesn’t know what they can or cannot do anymore. There’s a sense of losing oneself, which inevitably affects their relationships with others. If the impact is on my self-relationship, it will affect my relationships with others as well.” (ID: 4H) - “A young adult, in their twenties, who is building their life and thinking about their future—education, family—faces this differently. There’s concern and difficulty because it challenges what were once their plans, dreams, and future.” (ID: 9H) |
| - Aids Makes Me Feel Like My Health has Worsened (HP) | - “[...] Regarding assistive devices, it’s also important to remember that one has worsened. I have many patients, especially women, who say to me, ‘Well, I’m not going to switch from a cane to a walker because that means I’ve gotten worse.’ It’s almost like they think, ‘As long as I don’t change, I have the objective evidence that I’m not getting worse,’ and this reflects a psychological perspective.” (ID: 16H) |
| - Being Sick (PwMS; HP) | - “From a psychological perspective […] I live with the understanding that my day can no longer be like it used to be, and that makes me feel unwell.” (ID: 1P) |
| - - DMT Assumption (HP) | - “It’s not just about the side effects; it’s the fact that ‘I’m getting treatment, which reminds me that I’m sick.’ It doesn’t matter if the therapy doesn’t bother me; I still have to take it, right? I know that twice a day I need to take that medication, or that every six months I have to go for an infusion. It’s really about the reminder that I am unwell.” (ID: 11H) - “[...] I see that some people are happy to undergo treatment; for them, therapy becomes a reassuring aspect for the future. For others, however, it’s the opposite—it’s a reminder that ‘I’m sick,’ which creates a distinctly negative psychological aspect.” (ID: 11H) - “I notice the effect of health perceptions on quality of life, particularly in relation to medication. For patients undergoing therapies that lead to significant immunosuppression, they have to take precautions that also impact their social lives. Yes, I really feel this impact. In fact, these individuals tend to experience more severe infections when they get sick. The awareness that those infections, in their severity, are linked to the fact that they are undergoing treatment for multiple sclerosis reinforces the idea of how much this condition ultimately affects their lives.” (ID: 4H) |
| - - Asking for Help Due to MS Symptoms (PwMS) | - “Even though they let me keep my driving license, I know very well that I can’t see properly out of this eye, so I don’t drive at night. In social situations, it means someone has to pick me up, and that makes me feel unwell.” (ID: 1P) - “I find it frustrating to have to ask for help. I see my illness as a sort of… forgive me for using the term, flaw. It’s true that it’s not my fault, as someone mentioned earlier, but I still feel annoyed having to ask for something that I wouldn’t have needed to if I weren’t ill. Because I end up needing to ask for many things due to the illness, it impacts me. So if I can, I try to arrange everything in a way that I don’t even have to let others know… to feel, so to speak, less ill when I say it.” (ID: 7P) |
| - - Legal Disability Request (HP) | - “I think that from a psychological standpoint, it’s very, very impactful to have to decide, for example, to apply for disability benefits. The term ‘disability’ is horrible. Who wants to identify with the label of being disabled? Considering that you’re considered disabled starting at a 33% impairment, you can be classified as disabled even with a condition that’s not visible or that has very little physical impact. From a psychological perspective, making this choice is truly disruptive.” (ID: 2H) |
| - Being Fragile (PwMS) | - “There’s an awareness of being obviously more fragile and not knowing what might happen to you in certain situations beyond MS, from a health perspective. So, there’s this feeling that, while I used to consider myself healthy, now I say, ‘Well, I’m more fragile than before because MS has an impact.’” (ID: 11P) - “Certainly, I see myself worse than I did before the diagnosis. There’s this constant awareness of fragility, not just because of the illness but also due to all the health issues that can arise with age or for any reason.” (ID: 11P) - “One thing that I think hasn’t been mentioned is the impact—not just the physical impact, but also the impact of therapies and the planning of the entire diagnostic process on life. For instance, consider someone newly diagnosed who might still have few symptoms. There may not be much change in their movement or self-perception, but there’s this whole new way of engaging with the healthcare system, which they might have preferred to avoid. The planning of all these appointments required to start therapy and set up treatment disrupts their normal habits. Initially, there are frequent appointments, therapy consequences, side effects, and diagnostic tests. I believe the overall impact is certainly demanding. This also has psychological implications because, suddenly, a person feels vulnerable and dependent on others—on healthcare providers and specialists. Even if, over time, they develop personal resources to cope and move forward, initially, they definitely need help and find themselves in a difficult situation.” (ID: 6H) |
| Emotional Well-Being (PwMS; HP) | - “The illness unfortunately makes itself felt, even if it’s not very visible from the outside because I use a cane; I walk with it, but internally it’s quite impactful, and that’s what bothers me the most. My brother is very supportive, but I go through phases—sometimes I feel a bit depressed, especially when I think about the life I used to have. The impact has been pretty strong.” (ID: 8P) - “The suffering is significant, but there’s no other way to handle it. Otherwise, you get swallowed up by this illness, which really isn’t fair.” (ID: 11P) - “Who cares? If I’m like this, why should it matter to you? I’m living my life well despite everything, even if that’s not entirely true. But that’s something I only know inside.” (ID: 14P) - “I think that, in psychological terms, there’s almost always some level of impact.” (ID: 8H) - “I have balance issues and problems with walking, but the diagnosis of multiple sclerosis is really something that is very difficult to accept psychologically.” (ID: 7H) |
| - The Emotional Cost of Exams/Therapy/Rehabilitation (PwMS; HP) |  |
| - - DMT Impact (HP) | - “Sometimes there are people who are very scared of the infectious risks that certain therapies can pose.” (ID: 8H) - “And there's the constant reminder of the drama of the illness, because ‘this medication I take three times a week makes me feel sick the next day, or I have to deal with feeling unwell every other day... I feel bad because of what I’m doing’ to try to stop a disease that is, by definition, hard to halt.” (ID: 17H) - “There’s a young person who has to take a pill, but this still creates anxiety about the illness.” (ID: 11H) |
| - - - DMT Biological Impact (HP) | - “As long as they were using these injectable medications with side effects and unfortunately low effectiveness, there was a significant issue related to mood, including episodes of depression, and so on.” (ID: 17H) |
| - - Time Dedicated to Exams/Therapy/Rehabilitation (HP) | - “While undergoing more therapy can be beneficial, it also takes time away from loved ones. If there's an element of anxiety, individuals might feel they're sacrificing precious time with family to focus on their own care. This creates a conflict of will. Therefore, there’s definitely an impact from therapy in terms of how it consumes personal time, which is sometimes perceived negatively, as if ‘giving’ time to the illness.” (ID: 14H) - “From my experience, I constantly encounter complaints from users, whether they are newly diagnosed or in more advanced stages. There’s a heavy burden they feel when justifying their need for physical therapy or explaining why they must visit the hospital once a month for treatment. This is particularly true when there are no appropriate accommodations. They find themselves in a limbo where one side insists, ‘You must undergo rehabilitation, you must follow treatment,’ while on the other, they struggle to carve out time for these necessities, needing to justify having an illness, making choices, and establishing new priorities. This aspect has a significant psychological impact and also affects social interactions, especially in the workplace. I can't count how many discussions I've had trying to explain that someone is having difficulty balancing their family and work life with managing their illness, which inevitably becomes part of their daily life. The constant need to justify their situation is a crucial factor.” (ID: 15H) - “One aspect that hasn't been mentioned is the impact—not just physical, but also the effects of therapies and the planning of the entire diagnostic process on daily life. For instance, consider someone newly diagnosed who may not have many symptoms yet. While they might not perceive significant changes in their physical movement, they are suddenly immersed in a new healthcare environment they would have preferred to avoid. The planning of regular appointments for therapy initiation requires them to reorganize their usual routines. This includes navigating initial appointments every few months, dealing with side effects of treatment, and undergoing diagnostic tests. The overall impact is undoubtedly challenging. This also brings about a psychological effect, as individuals suddenly feel they are in need of support from others and require assistance from healthcare professionals. While they may eventually develop personal resources to manage, they initially face significant difficulties.” (ID: 6H) |
| - - Meeting People with a More Severe MS During Exams/Therapy/Rehabilitation (HP) | - “In the early stages, there’s an incredible embarrassment in coming to a facility where you might encounter, in the hallways or waiting rooms, individuals with more significant disabilities. This creates a kind of acceptance and direct confrontation with those who have had a diagnosis for a longer time. I believe this significantly impacts the psychological and, so to speak, social aspects, especially in the initial phases.” (ID: 16H) |
| - The Emotional Cost of MS Symptoms and Functional Limitations (PwMS; HP) | - “And this [the fatigue that limits social life] can only have a backward impact on psychological well-being. Because a restricted or sacrificed social life doesn’t contribute to the person’s psychological well-being or happiness in many areas, in all the areas they considered important. Probably social life was important before, but it remains important afterward; it just becomes less practicable.” (ID: 2H) - “Facilitator: Does this [memory problems] have an emotional impact for you? PwMS: Yes, emotional, because sometimes it also makes me forget things I’d like to remember, like the trips I’ve taken. I’ve almost immediately removed almost everything I did on those trips from my mind because there isn’t enough space for everything, so I have to make room for new memories, for work-related things, etc. And the others I have to put aside. So yes, that disappoints me.” (ID: 7P) |
| - - The Emotional Cost of Cognitive Impairment (PwMS; HP) | - “An ultimate observation regarding cognitive disturbances: I find a lot of difficulty, not always but quite often, in accepting cognitive rehabilitation. Because, except for cases like the one I mentioned earlier about the girl, there is often an attitude of denial, of not accepting the disturbance, because it somehow causes discomfort. Accepting that one has cognitive disturbances becomes a negative stigma for some, so when a rehabilitative approach is proposed... we find it challenging to get them to accept it.” (ID: 11H) - “...as if, deep down, they don’t want to accept it, right? Something that hurts so much that you prefer to avoid it. Frankly, I see this more with cognitive disturbances. [...] I frequently encounter this negative reaction, this non-acceptance regarding cognitive disturbances. [...] Generally, there’s quite a negative attitude, probably due to the non-acceptance of the disturbance, feeling that it’s considered negative by family and others.” (ID: 11H) - “However, in most people, the idea persists that ‘since I’m not functioning well physically, at least I’m functioning well cognitively,’ because there can be some adaptation at the family, work, and social levels. So, admitting to a significant cognitive problem is difficult; often, therapies for cognitive issues don’t have great hopes of being truly effective, and cognitive rehabilitation isn’t always readily available... so it becomes a taboo for the patient themselves.” (ID: 17H) - “I’ll give a practical example: we currently have a young person with a recent disorder who came to us from another hospital and is undergoing a program here. We usually conduct a cognitive evaluation, which showed attention disturbances, and this really agitated her... The acceptance of this issue has created such difficulty for her; while she’s managing her walking difficulties relatively well, the fact that she has memory disturbances has completely unsettled her.” (ID: 11H) - “Facilitator: So not only the direct impact of the symptom, which may cause someone to perform worse, but also a sense of shame in discovering that you have [cognitive difficulties]... HP: Yes, it’s like saying... as if, deep down, they don’t want to accept it, right? Something that hurts so much that you prefer to avoid it.” (ID: 11H) - “You feel insecure, maybe when facing certain situations. Sometimes I find myself making mistakes with budgeting or similar things, you know? These immediate things that they ask you... it can be frustrating at times because you think, ‘Why did I make this silly mistake? Why did I get this wrong?’ It weighs on you a bit.” (ID: 10P) |
| - - Anxiety Due to Bowel and Bladder Incontinence (PwMS; HP) | - “However, the thing that bothers me the most is the urinary urgency. And it's because of the retention, since I do two self-catheterizations a day. That’s something that bothers me a bit, let’s say, because I’ve also had two infections. Besides the catheters, it’s also going out and thinking that I might suddenly need to go, because then I have urgency incontinence. When it doesn’t hit me, it’s fine, but when I feel the urgency and there isn’t a bathroom within a minute... I basically [pee] outside. So I also use adult diapers that I buy at the pharmacy, but that’s something that causes me anxiety because the first thing I think about when I have to go somewhere is whether there’s a bathroom nearby.” (ID: 8P) - “Even now I have limits like [another participant’s name], if I’m not mistaken. I use an external catheter because unfortunately I can’t do without it, and it definitely affects me psychologically. It limits you mentally […] For a while, I was afraid to leave the house; I wasn’t comfortable until I accepted this solution, and now I live with it more or less calmly, let’s say.” (ID: 4P) - “They see, however, that the issue of urinary incontinence and urinary urgency is much more important or necessary to address, as it creates difficulties from a social and work perspective. Having to perform self-catheterizations and experiencing the urgency that makes you go to the bathroom ten times a day, along with the constant fear of infections and incomplete emptying, is obviously a priority for them.” (ID: 5H) |
| - - Fatigue/Fatigability: Impact on Social Relationships (HP) | - And this [the fatigue that limits social life] can only have a backward impact on psychological well-being. Because a restricted or sacrificed social life does not contribute to the person's psychological well-being or their happiness in many areas, in all the areas they considered important. Probably social life was important before, but now it remains important, yet it is no longer feasible, perhaps.” (ID: 2H) |
| - - Depressive Mood Due to Pain (PwMS) | - "When I feel physically well, I wake up smiling. This means that my physical state greatly influences my psychological well-being. When I feel good and free of pain, I approach the day with a more open mindset. Conversely, if I am in pain, I tend to experience a sense of mild depression that is always linked to physical discomfort." (ID: 9P) - "Facilitator: If I understood correctly, you touched on all dimensions. There were functions that were limited, as the neurologist noted, due to the presence of pain. Additionally, your mood seemed to be lowered, likely due to significant worry and a loss of enthusiasm. There were also limitations regarding your activities…   PwMS: Yes, I couldn't walk, which restricted my activities. I was on sick leave from work, something that didn’t even happen when I was on interferon." (ID: 12P)   - "Pain can be overwhelming because, in my opinion, it takes a toll on you psychologically. It's always there, and it makes you disregard everything else." (ID: 11P) |
| - - Sexual Function and Satisfaction (PwMS; HP) | - "I am unfortunately excluding all sentimental and sexual aspects from my life for self-protection." (ID: 2P) - "However, it sometimes happens that, especially among older men, it is generally assumed that sexual life is no longer relevant, as if it were a thought to be abandoned. But I’m not talking about nonagenarians; I mean men in their fifties and sixties. Younger people often feel a lot of shame because the sexual aspect has repercussions on the couple, particularly concerning self-image and one’s role within the relationship. If the partner is assisting with personal care, like helping you wash or with catheterization, that entire sphere tends to be sidelined. The impact is certainly significant." (ID: 2H) - "There is definitely an impact, and the younger you are, the more complicated it becomes. Take, for example, the issue of urinary urgency. Thinking of oneself as a performer in a sexual context becomes much more challenging and impactful." (ID: 2H) |
| - - Autonomy Loss (PwMS; HP) | - "Facilitator: You mentioned that this has an emotional impact, is that correct? PwMS: Yes, absolutely. It has an emotional impact because you realize you can no longer do the things you used to, and you have to reassess certain aspects of your life. That’s undeniable." (ID: 11P) - "The inability to take a walk or swim, to get in and out of the water—one might think these are relatively minor issues. But they’re not. It weighs heavily on me not being able to take a nice walk. It’s a significant limitation, and that can trigger important psychological challenges." (ID: 11P) - "Facilitator: Beyond the objective limitations, does it impact how you feel emotionally? PwMS: Yes, yes… it does, because you always feel inadequate, right? Well, maybe not completely inadequate, but you definitely feel stuck in things you would otherwise do. You feel a bit useless, like you’re just there… and sometimes you wish you could be more helpful, but you can’t." (ID: 14P) - "I had to give up driving because my hands don’t grip the steering wheel properly anymore. As a result, I've progressively lost my autonomy, and what impacts me the most psychologically is the dependence on others. I’ve always been a very independent person..." (ID: 9P) - "Dependency is truly very limiting psychologically." (ID: 9P) - "There’s clearly embarrassment in having to ask others for help with intimate matters." (ID: 9P) - "At the end of the day, to go for a half-hour appointment at the hospital… I arrive home exhausted. So, for me, the real issue is having to depend on others, and that weighs heavily. I often think, 'I’ll limit this as much as possible; I have everything I need at home, and when I can, I go out occasionally.' But it weighs a lot on me, and this is my experience… the fact of having to depend on others." (ID: 13P) - "And then, for example, going to the hospital, what weighs on me tremendously is having to depend on others. That’s just unbelievable." (ID: 13P) |
| - The Emotional Cost of Using Assistive Devices/Aids (PwMS; HP) | - "HP: I've also thought about the impact on the patient when they have to start using an assistive device.   Facilitator: How do you think it impacts them?  HP: I believe it has a lot to do with judgment—specifically, the judgment of others..." (ID: 13H)   - "It means that when it comes to assistive devices, there's motor difficulty and the embarrassment of being judged by others..." (ID: 16H) - "At first, I used to walk—sure, not very well—but I didn't want to be seen. I thought, 'Why is this so hard?' But in the end, it is what it is. Once I accepted it, I found that people respect you more because they can see you’ve come to terms with your condition." (ID: 11P) |
| - The Emotional Cost of MS Impact on Working Activities (PwMS) | - "My job is very important to me. I work as a pediatric nurse, and during the first few years when I felt well, I was able to do the work I love—the work I was probably meant to do. However, when I started interferon therapy 15 years ago, which was the only option at the time, I had to give up my role. The medical occupational health service reassigned me to lighter duties, so from a professional standpoint, I was well protected. Yet, I cried for ten years every time I shared this story. It wasn’t a choice; it was something I had to endure." (ID: 12P) |
| - Family Relationships (PwMS; HP) |  |
| - - Impact of MS on Getting Married (HP) | - “Many of them got married very young, often right after a recent diagnosis, and I was initially surprised by that. From my perspective, I only saw the negative aspects, the fear of the future. But many seize this opportunity to push themselves and move forward. So, it’s also a way to turn a significant, often unthinkable event into something positive.” (ID:14H) |
| - - Impact of MS and Treatments on the Pregnancy Project (HP) | - "There is certainly an impact on quality of life regarding the potential plans for pregnancy, particularly as these plans are affected by the diagnosis. Stability in the disease is often required, necessitating new therapies and certain medical assessments, which can interrupt their plans and cause setbacks. Psychologically, they grapple with many questions: Will they be able to care for the baby? Will the pregnancy proceed normally? Will there be complications during delivery? Many women are terrified about the possibility of not being able to give birth like other women. Some are even more concerned about breastfeeding, knowing they may need to resume treatments or undergo immediate neurological evaluations, which could prevent them from breastfeeding. This worry can be more distressing than fears of relapse or rebound effects, as breastfeeding is perceived as an essential part of caregiving—a piece that is taken away from them." (ID: 5H) - "[The impact of MS on pregnancy plans] also affects family dynamics. These women often feel guilty toward their partners, who desire a pregnancy while they feel compelled to halt their plans due to the emerging challenges." (ID: 5H) |
| - - Fear of Genetically Transmitting the Illness to my Children (HP) | - "Facilitator: What do you think causes a short circuit in these fathers when a baby arrives?   HP: [...] Perhaps it's the fear of potentially passing something on to the child. Genetic factors are a concern, and the idea of heredity is a nagging worry for any parent, not just for those with MS, but also for other less severe conditions." (ID: 15H) |
| - - Fear of Not Being an Adequate Parent (PwMS; HP) | - "HP: Recently, we've seen quite a few patients—more men than women—who were diagnosed some time ago and are about to become fathers. As the due date approaches, everything seems to crumble, and they seek psychological support, even though they were previously convinced they didn’t need it. - Facilitator: What do you think causes a short circuit in these fathers when a baby arrives?   HP: I believe it brings about fears, such as 'How will I be able to support them? How will I raise them? What kind of example can I set for them?' (ID: 15H)   - Among the most important factors for quality of life is family. Especially in my case, having a small child, I feel a strong desire to do a million things with him that I could have done a few years ago but can no longer manage. Now I feel like it’s slipping out of my hands. I sometimes worry about how much I can physically give him. I try to do my best in other ways, but physically, I wish I could take him to the park or play soccer or basketball with him. I played basketball for 27 years, so I want to teach him a lot, but I’m not sure if I’ll be able to. I might be able to participate in some way, but will I be able to do it the way I like or in a way that he would enjoy? He might still appreciate my presence, but it could feel different for me—not as fulfilling. Of course, being with him is always wonderful, but it feels different." (ID: 14P) |
| - Authenticity/Inauthenticity (PwMS; HP) | - "I’ve shed a lot of the social facades I used to have. I wouldn’t say I had many, but now I’m much more transparent and direct with people. I no longer worry about how I present myself, whether it’s for convention or the situation. I just think, 'This is me; accept me as I am.' Of course, I still maintain politeness, but those social layers that I used to feel were necessary are gone." (ID: 11P) - "When there is an impact, it conditions spontaneity, leading, unfortunately, to inauthentic behaviors that can distort a person's individuality. The illness affects one's identity, and from my perspective, the psychological impact is always present. It transcends physical and social realms, as there is always a psychological consequence.   The individual feels disoriented, loses their reference points, and becomes unsure of what they can or cannot do. This phase is characterized by a profound sense of loss of self, which in turn affects relationships with others. If the impact is on my relationship with myself, it inevitably influences my interactions with others. (ID: 4H)" |
| - Increased Sensitivity (PwMS) | - “It increases sensitivity to aspects that one might have overlooked before, like how you wouldn’t notice a pothole in the road when you’re walking fine, right? So, with the illness, many receptors get activated, allowing you to perceive things—especially from a human perspective—that you didn’t notice before.” (ID: 9P) |
| MS is a Stressor (PwMS; HP) | - “Facilitator: Would you say that the illness is a stressor? That is, a source of stress?   HP: I would say yes, especially when I don’t know where I’m going.” (ID: 13H)   - “The psychological aspect has significantly influenced my experience. I am a very controlling person, and this illness is the least suited to someone like me. Every day, I wake up wondering if my leg will worsen. Since the diagnosis, I feel like I’m walking on eggshells.” (ID: 1P) - “Feeling unwell worries me that it might lead to depression, which in turn causes anxiety. I struggle to sleep well, and not feeling 100% has a clear psychological impact. That's why I immediately sought psychological support.” (ID: 1P) - “Facilitator: So, in this case, could we say that the illness is indeed a stressor?   PwMS: Absolutely, it’s a source of stress. It’s unpredictable—you never know what today or a year from now will be like. You can read everything and hear many opinions, but no one can tell you how it will evolve. It’s true that no one knows how their life will progress, but you’re being told you have something that will change over time.” (ID: 1P) |

**Table S1.3** List of HRQoL Domains (all categories) in the Social Domain

| **Social Domain** |
| --- |
| Support (PwMS; HP) |
| - Limited Ability in Providing Support to Others (PwMS; HP) |
| - - Due to MS Impairment (PwMS; HP) |
| - - - Parenting (PwMS; HP) |
| - - - Other Relatives (HP) |
| - - - Friends (HP) |
| - - Due to MS Psychological Burden (PwMS) |
| - Limited Ability in Seeking Support (PwMS; HP) |
| Social Connectedness (PwMS; HP) |
| - The Social Consequences of Invisible Symptoms (i.e. Fatigue/fatigability) (PwMS; HP) |
| - - The Social Consequences of Invisible Symptoms Being Misunderstood (PwMS; HP) |
| - The Social Consequences of Bowel and Bladder Incontinence (PwMS; HP) |
| - The Social Consequences of Time Dedicated to Exams/Therapy/Rehabilitation (HP) |
| - The Social Consequences of Hiding the Diagnosis (HP) |
| - The Impact of Illness Representation on Romantic Relationships (PwMS; HP) |
| - The Impact of Sexuality Dysfunction on Intimate Relationships (HP) |
| Self-Perception in Social Settings (PwMS; HP) |
| - Symptom-Related Stigma (HP) |
| - Feeling Vulnerable (PwMS) |
| - Perceiving Others' Pity (PwMS) |
| Social Participation (PwMS; HP) |
| - Social Life (PwMS; HP) |
| - Family Activities (PwMS; HP) |
| - Personal Time (HP) |
| - Hobbies and Leisure Time Activities (PwMS; HP) |
| - - Fear of the Disease Limits Travelling (PwMS) |
| - - Limitation in Playing Sports (PwMS) |
| Work (PwMS; HP) |
| - Work Difficulties Due to MS Symptoms (PwMS; HP) |
| - - Work Difficulties Due to Cognitive Impairments (PwMS; HP) |
| - - Work Difficulties Due to Visual Impairments (PwMS) |
| - - Work Difficulties Due to Invisible Symptoms (HP) |
| - Impact of Changes in Work Activities Due to MS (PwMS; HP) |
| - Burden of Medical Leave Requirements (PwMS; HP) |
| - Workplace Discrimination and Prejudice (PwMS) |
| Finances (PwMS; HP) |
| - Financial Impact of Disease Management (HP; PwMS) |
| - - Formal Caregiver Costs (PwMS) |
| - - Private Health Assistance Costs (PwMS; HP) |
| - - Driving License Renewal Costs (PwMS) |
| - - Formula Milk Costs (HP) |
| - Financial Impact of Work-Related Issues (HP; PwMS) |
| - - Financial Risk of Freelancing (PwMS) |
| - - Risk of Job Loss or Demotion (HP) |

**Table S1.3.1** List of HRQoL Domains (all categories) in the Social Domain with quotes

| **Social Domain** | - "The onset of the disease can, in some cases, change the way I see myself in the world and how I position myself within it. Consequently, this can also have a social rebound effect." (ID: H1) |
| --- | --- |
| Support (PwMS; HP) |  |
| - Limited Ability in Providing Support to Others (PwMS; HP) |  |
| - - Due to MS Impairment (PwMS; HP) | - “Facilitator: Do you feel that the illness limits your ability to provide or receive support from others?   PwMS: […] There’s usually a lot of understanding from others… more or less. They try not to make it a big deal, you know? But being in the situation yourself, you want to help out or be useful, and you just can’t. You can't do it in any way.” (ID: 14P) |
| - - - Parenting (PwMS; HP) | - Facilitator: "What do you think causes a sort of mental clash for these dads when a baby arrives?"   HP: "I think it brings up fears like, 'How will I manage to take care of them? How will I raise them? What kind of example can I set for them?'” (ID: 15H)   - Facilitator: "Does the disease impact the ability to give and receive support, and does this affect quality of life?"   HP: "Yes, it does, and I think we've touched on this. For example, when considering how to support others as a parent, like picking up kids from school, it might seem trivial, but that's a form of support. There's also the concern about not being able to manage this in the future, especially in terms of parenting." (ID: 10H)   - “One of the top things for quality of life is family. Especially in my case, since I have a young child, and there are so many things I wanted to do with him that I could have done a few years ago but can’t do now… I feel like I’m losing control over this situation. Sometimes I worry about how much I’ll be able to physically give him. I always try to do my best, but physically, I’d like to take him to the park, play soccer, or basketball. I played basketball for 27 years, so I wanted to teach him a lot of things, and I’m not sure if I’ll be able to do that or if I’ll ever be able to do it. Maybe I can, but will I be able to do it the way I’d like or how I think he might enjoy it? He might still like it because I’m there, but it might be different for me… it might not be as fulfilling because, of course, spending time with him is always wonderful, but it’s just different.” (ID: 14P) - “The way people manage time changes significantly due to the disease. It greatly affects not only the time spent on self-care but also the time required for everyday tasks. Activities that once were quick now take twice or even three times as long. For instance, I may no longer be able to take my children to school as I used to, because I have to allocate more time for my own care.” (ID: 2H) |
| - - - Other Relatives (HP) | - "The impact is significant. As we've discussed, people often struggle with supporting themselves, let alone others, both emotionally and physically. There's also concern about future capabilities, especially in caregiving roles for aging parents. The disease's physical and emotional toll can greatly affect one's sense of usefulness and ability to support others." (ID: 10H) - “Autonomy often becomes a concern when it comes to fulfilling grandparent roles. Many of my patients are grandparents, some even great-grandparents, and they want to fully engage in their grandparenting duties.” (ID: 8H) |
| - - - Friends (HP) | - Facilitator: "The aspect of requesting and providing support to others seems somewhat overlooked. Do you think the disease affects this, and does it impact quality of life?"   HP: "Yes, I believe it does, as we've discussed. For example, many people face the challenge of feeling unable to support others, such as friends, because they can't even support themselves, both emotionally and physically." (ID: 10H) |
| - - Due to MS Psychological Burden (PwMS) | - "Regarding providing help, I've noticed that as my suffering progresses—meaning my pain and discomfort—I've become less patient with others' complaints. Everyone has their own pain, but sometimes I'm in so much pain that I find it hard to empathize when someone complains about something minor. I don’t like this aspect of myself, but it’s a reality I've noticed; my empathy has diminished a bit." (ID: 9P) |
| - Limited Ability in Seeking Support (PwMS; HP) | - Facilitator: Do you think this also affects how likely someone is to ask for help? Might a person feel like they’re a burden and therefore limit their requests for assistance, negatively impacting their quality of life?   HP: Yes, it can. For instance, imagine we have a headache. If someone asks me for something when I have a headache, I might not mention my discomfort because I don’t want to be a burden. So, if we think that the person we’re interacting with has limitations or might have them in the future, we’re more likely to offer support rather than seek it. We tend to focus on giving help instead of receiving it. (ID: 10H)   - Facilitator: In your case, has knowing about your illness changed how you feel about asking for help or taking leave? Do you feel like you have to prove you’re not sick, and that holds you back?   PwMS: Yes, it’s a bit like that. It’s as if you always have to show you can do everything like everyone else. So, if you ask for a favor or take time off, you feel like you’re constantly proving that the illness isn’t a problem for you.  Facilitator: And does that sometimes lead you to ask for less help, just to prove you can handle things on your own?  PwMS: Definitely. (ID: 7P)   - Facilitator: Have you ever found that the illness negatively affects your ability to ask for support?   PwMS: That whole aspect has changed because I try not to ask for help. I only ask when I absolutely need to, but I push through as long as I can without asking.  Facilitator: Do you think this ultimately has a negative impact on your quality of life?  PwMS: Yes and no. When I manage on my own, I feel proud. But when I fail, it always feels like a defeat. And when it’s just one defeat, fine, but when it’s 100, it becomes overwhelming. (ID: 14P)   - PwMS: At times, I’ve felt like I was bothering others. I’d think, “Why should I complain or ask for help with everything?” Even though others are often willing to help, sometimes pride or the desire to do things on your own holds you back. But this can limit you in small things, where just asking would have been enough. As ID: 14P said, you want to prove you can do it alone, but in some cases, it holds you back, even when others are willing to help. I’m speaking generally here, not about specific cases. (ID: 13P) - HP: There’s a lot of physical exhaustion, but also the mental effort of holding it all together, of not being able to let go, and of not asking for help. Not because there’s no one to help, but because it’s difficult to ask.   Facilitator: So, do you think the way people ask for help changes with illness?  HP: I think so. It also depends on the person. If someone wasn’t used to asking for help before, then when illness comes... forget it! Before they ask, they’ll want to show they’re tough and can manage on their own. (ID: 1H) |
| Social Connectedness (PwMS; HP) | - "Interpersonal relationships have obviously deteriorated. Real friends stick around, but everyone else gradually fades away. So, in the end, it's a matter of having fewer but more meaningful connections."** (ID: 13P) - "Yes, there’s probably some change in how a person interacts with others, whether it's due to physical limitations or not." (ID 1H) - "Socially, the most noticeable effect is that the illness acts as a filter. It helps them discern the value of certain relationships over others, leading to more conscious choices. This gives them stronger motivations to eliminate toxic relationships and form deeper connections." (ID: 4H) |
| - The Social Consequences of Invisible Symptoms (i.e. Fatigue/Fatigability) (PwMS; HP) | - "They are often overwhelmed from a rehabilitation perspective… overwhelmed might not be the best term, but they are subjected to many different types of treatments, and this definitely impacts their social life, as well as fatigue. Just yesterday, I spoke with someone who had just finished a 45-minute water rehabilitation session. They were exhausted—completely worn out but happy, recognizing that it was an effective therapy for them. However, they also said, 'When I get home, I can't wait to lie on the couch because I need to recover from the effort of the therapy.' So, this definitely affects their social life, leaving less time for other activities." (ID: 16H) - "Honestly, when it comes to the impact on one’s life, social interactions, and work, fatigue—the exhaustion and quick weariness—plays a much more significant role." (ID: 17H) - "Fatigue greatly limits you because you realize you have fewer opportunities for face-to-face interactions. It’s all about managing and conserving your energy throughout the day. For example, if I plan to go out one evening with a friend or visit someone, I might manage to do it once a week at best, because it takes so much effort. As a result, my social interactions become more sparse." (ID: 11P) - "Even though some people don’t show obvious physical symptoms, there’s an invisible part that’s sometimes mistaken for laziness—both at work and, to some extent, in family life." (ID: 8H) - "Fatigue… we were talking earlier about invisible symptoms, and it’s one of the most impactful symptoms at every stage of the disease. If I’m a manager working eight hours a day—let’s say I’m 30 and working 12 hours, giving my best—by Friday night, I might want to go out for a beer with friends. But I might not be able to, because I’ve used all my energy being extremely productive at work, and I’ve done it. But come evening, I collapse on the couch and fall asleep by 9:30, while my 30-year-old colleague is out at Alcatraz having a beer. So, the social side of things definitely suffers a lot, sometimes even the most from these symptoms." (ID: 2H) |
| - - The Social Consequences of Invisible Symptoms Being Misunderstood (PwMS; HP) | - “I mean, my husband doesn’t fully understand what I’m going through, even though he’s always been there for me. It took time for him to realize, and I’ve wondered if it’s just that he’s slow to grasp it or if it’s really difficult to understand. It’s one of those illnesses that’s hard to share.” (ID: 11P) - “People don’t understand the fatigue I experience. I often compare it to having to climb a mountain every day. That’s the level of effort I put in. Some might say, ‘You’re exaggerating,’ but no, that’s the reality. Every morning, I have to push through, and while I’ve never been accused of being lazy—I’ve always kept busy—I know that they just don’t get it. It’s something they truly can’t comprehend.” (ID: 11P) - “Even when people have visible limitations, there’s often an invisible part that’s dismissed as unwillingness to engage, whether in work or family life.” (ID: 8H) - “The concept of time is crucial. When I walk poorly, I realize all the things I can no longer do. My time unit remains the same, but my capacity decreases; I used to accomplish five tasks in an hour, and now I can only manage two. This has a significant impact and often correlates with various invisible aspects that are the hardest to communicate, whether related to work, family, or friendships. Friends don’t understand why I’m too tired but still feel the need to participate in activities.” (ID: 8H) - “What always surprises me is how people with MS are, in a sense, overlooked—not fully understood, really, because of the nature of the disease. I’ve encountered neurologists and doctors who just don’t go beyond their own understanding. They may prescribe treatments, but they don’t grasp the symptoms I experience, the fatigue, or the psychological aspects that weigh heavily on me.” (ID: 11P) |
| - The Social Consequences Bowel and Bladder Incontinence (PwMS; HP) | - "In reality, you live in denial until you reach a point where you have to go to the bathroom every half hour or hour, and the result is either you know every café in the city during your travels, or you stop going out altogether. Like it’s been said, you stop going out because you can’t do those activities. It’s a taboo topic as well." (ID: 17H) - "But the thing that bothers me the most is, let’s say, the urinary urgency and retention. I do two self-catheterizations a day, and that’s something that annoys me a bit because I’ve also had two infections because of the catheters. Then there’s also the anxiety of being out on the street, knowing that the urgency could hit me, and I have urgency incontinence. It doesn’t happen all the time, but when it does and I can’t find a bathroom in one minute… well, I end up going in public. So, I also use diapers that I buy from the pharmacy, but that gives me anxiety. The first thing I think about when I go somewhere is whether there’s a bathroom nearby." (ID: 8P) - "I: So, has it ever limited you, like have you ever decided not to go out because you were afraid of not finding a bathroom? Does it influence your social decisions? PwMS: Yes, it influences them!" (ID: 8P) - "Even now, I have limitations. Like [name of another participant] said, I use an external catheter because, unfortunately, I can’t do without it, and it definitely affects me psychologically. It limits me mentally. Not anymore, but I went through a period where I stopped going out because I couldn’t control the situation, and I had some really embarrassing moments. I wasn’t going out anymore… I felt a lot of shame because I had experiences that really… For a while, I was afraid to leave the house. I wasn’t comfortable until I accepted this solution. Now, I live with it more or less peacefully." (ID: 4P) - "Many patients with sphincter disorders, urinary and faecal issues, really struggle. If they can’t find some form of control with the help of the rehabilitation team, for them, sexual function becomes a secondary concern. Those symptoms are hard to hide, they’re visible, and they have a social impact." (ID: 4H) - "They see the issue of urinary incontinence and urgency as far more important, something they need to resolve because it creates difficulties in their social and work life." (ID: 5H) |
| - The Social Consequences of the Time Dedicated to Exams/Therapy/Rehabilitation (HP) | - "So, doing more therapy on the one hand is beneficial, but on the other hand, it takes time away from loved ones. If there's an element of anxiety, they might feel like they’re losing time with loved ones by focusing on themselves. This creates a sort of conflict of intentions. Therefore, the impact of therapy is felt in how it takes away personal time, which is ‘given,’ in a sense, to the illness, and this can sometimes be perceived negatively." (ID: 14H) - "They’re often overwhelmed from a rehabilitation standpoint… 'overwhelmed' isn’t the right term, but they undergo a lot of different treatments, and this undoubtedly affects their social life, as well as their fatigue. Just yesterday, I heard from someone who had a water-based rehabilitation session. After 45 minutes in the pool, they came out exhausted. Exhausted, but happy because they recognized that the therapy is very effective for them, but then they said, 'I get home and can’t wait to lie on the couch because I need to recover from the effort I’ve just put in and from the therapy itself.' So, this definitely affects their social life, leaving less time for other activities." (ID: 16H) - "From my experience, I often deal with complaints from patients, whether it’s a new diagnosis or a more advanced one. These people carry the burden of having to justify themselves. They have to explain why they go to physiotherapy, why once a month they need to go to the hospital for treatment, and all this without proper support in place. From a bureaucratic standpoint, they’re stuck in a limbo where, on one hand, they’re told, 'You need to do rehabilitation, you need therapy, you need treatment,' but on the other hand, they must find the time to do it, while also having to justify it. Justify the fact that they have an illness, that they need care, that they need to make choices, that they need to create a new list of priorities. This has a psychological impact for sure, but also a social one. You can especially see it at the workplace. I can’t count how many times I’ve had to explain how someone struggles to balance their family and work life with their health care needs, which deeply affects their life. This constant need to justify is, in my opinion, a very significant issue." (ID: 15H) - "Therapy itself, and the fact that you have appointments and deadlines for exams, can also affect how you manage your family life." (ID: 8H) |
| - The Social Consequences of Hiding the Diagnosis (HP) | - Facilitator: "You mentioned at one point that people with MS live in a context where others might have their own opinions about the disease. Do you think the diagnosis itself can interfere with the relationships a person creates or maintains over time? Is evaluating the impact of the disease on relationships an important factor in someone’s quality of life?"   HP: "Yes, it’s very important. It’s somewhat implied in what I said earlier, in the sense that patients, if they can, often don’t share their diagnosis. This is because they have multiple sclerosis, not glomerulonephritis, which is a condition people might talk about more openly, even though it could be much worse. As a result, many tend to keep it hidden. But just because it’s hidden from the outside, in most social contexts, doesn’t mean it’s hidden within. Inside, it weighs heavily on them, and they might only share it with the people closest to them. So, it’s extremely impactful, even before any physical symptoms of the disease appear." (ID: 17H)   - HP: "For example, let’s say I’ve just been diagnosed, and today I’m meeting my friends for drinks. None of them know about it. In my head, I’m thinking, ‘I need to be careful not to let anything slip.’ This might make me seem more tense or uptight. My friends might notice and ask, ‘Why do you seem so worried? Is something wrong?’ And there’s always that one friend who keeps pushing, ‘Come on, tell me what’s going on!’ But I’d just say, ‘No, no, it’s fine, nothing’s wrong.’"   Facilitator: "So, can it become a barrier to maintaining relationships?"  HP: "Yes!" (ID: 1H)   - "We're talking about those who are newly diagnosed, but this also applies to patients with more advanced stages of the disease. For example, someone in a wheelchair might think, 'I won’t go out because I don’t want to be seen in the wheelchair. I avoid using it because I want to show that I can walk.' Right?" (ID: 1H) |
| - The Impact of Illness Representation on Romantic Relationships (PwMS; HP) | - “My boyfriend left me after seven years, and I’m not sure, but I think his fear of the future played a big role.” (ID: 7P) - “Then there’s the issue of the difficulties in finding someone who will accept you and your illness, and that’s... well, that’s another chapter.” (ID: 10P) - “Facilitator: Has your illness ever been an obstacle in romantic relationships?   PwMS: Yes, it has happened... I wouldn’t say I’m a great romantic. I’ve had three relationships; two didn’t go well, partly because of that. One, the longest, knew about my illness from the start. But it ended for other reasons. Still, it’s an obstacle; it brings stress because you’re unsure when to bring it up. It’s hard to introduce, you know? You worry about respect for the other person—when is the right time? Is it too early or too late? You don’t know how they’ll react, so it’s a bit complicated.” (ID: 10P)   - “Even in family life, the uncertainty about the future, what will happen to me, how I’ll cope... if I have a partner, will the relationship last? Will they be able to support me, or are we close enough? […] Sure, it’s all surmountable, but this doubt definitely affects the quality of the relationship and the quality of life.” (ID: 6H) |
| - The Impact of Sexuality Dysfunction on Intimate Relationships (HP) | - “[The difficulties with sexuality] have repercussions within the couple, especially regarding self-image and one’s role in the relationship. If your partner assists you—whether it’s helping you wash or with catheterization—that whole aspect gets pushed aside. So, the impact is definitely significant.” (ID: 2H) |
| Self-Perception in Social Settings (PwMS; HP) | - “Facilitator: So, do you think having MS may change how a person perceives themselves in social contexts?   HP: I believe it can, and this definitely impacts quality of life. For example, someone might end up going out less, simply put.” (ID: 1H) |
| - Symptom-Related Stigma (HP) | - “Facilitator: Do you think that the difficulty in accepting cognitive symptoms is related to stigma and how people perceive themselves in social interactions?   HP: Yes, in a way, it is.” (ID: 11H)   - “In many cases of urinary and fecal incontinence, patients experience these issues as... if they can’t find a way, with the help of the rehabilitation team, to gain some control over these symptoms, then their sexual functioning takes a backseat. These symptoms are visible and have a significant social impact.” (ID: 4H) - “They often see issues related to urinary incontinence and urgency as much more critical problems to address, as these create difficulties in social and work contexts.” (ID: 5H) |
| - Feeling Vulnerable (PwMS) | - “A little has changed in terms of how I feel when I am around others. I feel like I’m now the person everyone always asks, ‘How are you?’—which wasn’t the question I used to get. Before, people would comment on my new haircut instead of asking how I was doing first. So yes, that has shifted. But it’s more the acquaintances rather than my close circle that have changed. Maybe I’ve changed too; I don’t want to put myself in situations where I feel tired or force myself to be social when I’m not up for it. So, it has made me feel vulnerable. Yes...” (ID: 1P) - Facilitator: “Has the disease influenced how you feel in various social settings?” PwMS: “Yes, mainly because of the confusion I felt in managing a situation I didn’t fully understand. I would often think, ‘What if in a month I’m stuck in bed and can’t move?’ And there’s also the ongoing issue of pain and suffering, which not everyone understands.” (ID: 11P) |
| - Perceiving Others’ Pity (PwMS) | - "What bothers me more is how others perceive my situation. It’s annoying to think that people might pity me or see me as burdened by my condition—using a wheelchair, crutches, struggling with my legs. I’m less concerned about my own experience and more about the judgment from others. I just accept my reality and do the best I can with what I have." (ID: 14P) |
| Social Participation (PwMS; HP) | - “It’s essential for me to be surrounded by sincere and strong relationships. So, between family and friends, I now have what I call a ‘domestic social life’ because I don’t go out much anymore. But those connections are still very present, and that’s fundamentally important to me right now. My social life may be less intense than it used to be, but the bonds are still very strong.” (ID: 9P) - “In terms of quantity, my social interactions have decreased, but not in quality. Overall, the quality is still quite high.” (ID: 9P) - “The pain has limited all my activities because I couldn’t walk; I couldn’t even work, which hadn’t happened to me even when I was on interferon.” (ID: 12P) - “It may not take away time from family, but if he comes home tired after a stressful day of therapy or physiotherapy, he won’t be as effective with the things he needs to do at home—family, work, and everything else.” (ID: 14H) - "People go through so many different treatments, and it really hits their social life and energy levels. I heard just yesterday from someone who, after a 45-minute water therapy session, was completely wiped out. They were happy it worked, but they needed to collapse on the couch as soon as they got home to recover. This definitely eats into their social life and the time they have for other activities." (ID 16H) - “It has influenced me a lot. Speaking from my own experience, I’m in a wheelchair, and there are many things I simply can’t do… public transportation is out of the question. For instance, when I need to go to the hospital, what weighs heavily on me is having to depend on others. It’s quite overwhelming. Sometimes I think, ‘Why should I go out? What’s the point?’” (ID: 13P) - “If I want to go out, I can’t even consider it if I want to play with Nicolò or if I need to take a shower. These are choices you have to make in the moment, knowing you have to give up something, even if you don’t want to.” (ID: 14P) - “Honestly, when it comes to the impact on life, social activities, and work, fatigue—exhaustion—is the most significant factor.” (ID: 17H) - “In reality, you live in denial until you reach a point where you have to go to the bathroom every half hour or hour. The result is that you either know all the restrooms in the city or you stop going out altogether. As mentioned, you don’t go out anymore because you can’t manage those activities; it becomes a taboo.” (ID: 17H) - “I still have limits like [another participant's name]. I use an external catheter because I can’t manage without it, and it definitely affects me psychologically. It limits you mentally. For a while, I didn’t go out because I couldn’t control the situation, and I felt embarrassed. I was afraid to leave the house; I just wasn’t comfortable until I accepted this solution. Now, I manage it more or less calmly.” (ID: 4P) - “Work is important, but having time for yourself is also crucial. In my view, the main component is still relationships—at least from what I see, having time for loved ones is essential. However, there’s also the need for personal time or engaging in other activities. For example, patients with a high level of functioning often prefer to do other things instead of rehabilitation.” (ID: 14H)"We're talking about those who are newly diagnosed, but this also applies to patients with more advanced stages of the disease. For example, someone in a wheelchair might think, 'I won’t go out because I don’t want to be seen in the wheelchair. I avoid using it because I want to show that I can walk.' Right?" (ID: 1H) |
| - Social Life (PwMS; HP) | - “I would also suggest focusing on the socialization of individuals. For some, being able to share not only difficulties but also enjoyable experiences can be extremely important. It’s essential to highlight in which contexts this sharing takes place. There are fun moments as well as opportunities for connection and sharing. For some, it might mean going out to the movies in the evening, while for others, it could simply be catching up via chat. For some, not having people to share with can be a significant limitation.” (ID: 10H). - “And yes, all personal relationships, outings, the things I no longer do—especially during these two years, 2022-2024. I’ve almost eliminated any interpersonal relationships, except for work-related ones.” (ID: 14P) - “Another aspect I deal with is fatigue, and I believe many people experience this. There are moments when my energy completely drains, like when your phone warns you that the battery is low. I just shut down. No matter where I am, I can’t think or do anything; I feel utterly exhausted. So in my social life, if I do something during the day with my kids or a friend, I know I won’t be going to the movies that evening. If I’ve worked all day and have plans tomorrow night, I’ll be staying home on the couch. At 48, this makes me feel a bit retired from life. But maybe I’ll learn to manage it; perhaps it’s just my fear, and it’s possible to adapt. In social situations, I’m holding back a bit.” (ID: 1P) - “Facilitator: Do you think fatigue has a social impact as well?   PwMS: t absolutely does. I mean, even just going out can feel exhausting. It might seem trivial, but even going to buy something can be tiring. Going to the pharmacy is a struggle. You don’t even feel like saying, ‘I’ll buy something for myself’ or ‘I’ll go out for a drink with a friend.’ It’s exhausting because you have to find the right place at the right time. You have to allocate a mix of energy to do that, and this limits you socially. You realize you have fewer opportunities for face-to-face interactions because everything hinges on the energy you have to spread out over the day. If I think about going out one evening with a friend, I might manage to do it once a week if I’m lucky, because it takes so much effort. So naturally, all relationships become a bit less frequent. True friendships remain, while the others—well, they tend to fade away.” (ID: 11P)   - “Fatigue… Earlier, we talked about invisible symptoms, and this is a symptom that has a significant impact on every level of the condition. If I’m a manager working eight hours, and I’m 30 years old, sometimes even 12 hours, giving it my all, it’s Friday night, and I want to go out for a beer with friends. I might not even make it out for that beer because I’ve exhausted all my energy to perform well at work. I’ve managed to do that, but then I collapse on the couch and by 9:30 PM, I’m asleep, while my colleague, who’s the same age as me, is out at Alcatraz after having a beer. So social life is definitely affected, and sometimes it feels like it’s the area most impacted by these symptoms.” (ID: 2H) - “Facilitator: Has this ever limited you? Have you ever chosen not to go out because you were worried about not finding a bathroom? Does this influence your social decisions?   Yes, it does!” (ID: 8P).  “Doing more therapy helps on one hand, but on the other hand, it takes time away from relationships.” (ID: 14H) |
| - Family Activities (PwMS; HP) | - “There’s a definite limitation. There are certain things you simply can’t do, period. Sometimes you might feel like you can manage, but most of the time, it’s just not possible. You either have no alternative or a mixed option that doesn’t really satisfy you. For example, I recently went to the snow for four days with my wife and son. I wanted to do so many things, but I ended up doing none of them. At least my son had fun; it was his first time in the snow. That’s fine, but I would have loved to take him skiing and try out the adaptive equipment we have for disabled skiers.” (ID: 14P) - “Also, having therapy sessions and appointments for tests can affect how I manage my family life.” (ID: 8H) - “So, while doing more therapy is beneficial in some ways, it also takes time away from relationships.” (ID: 14H) |
| - Personal Time (HP) | - “The impact of therapy, in terms of taking away personal time to 'gift' it to the illness, is sometimes perceived as negative.” (ID: 14H) - “This [the limitations imposed by multiple sclerosis] can obviously affect my activities, such as my interests in sports, travel, or any other activities I want to pursue during my days.” (ID: 8H) |
| - Hobbies and Leisure Time (PwMS; HP) | - “So this summer, my beautiful mountain experience has definitely changed. I’ve been going to the mountains since I was born, and it’s one of the fundamental parts of my life. But I can’t walk like I used to.” (ID: 1P) - “This [the limitations imposed by multiple sclerosis] can obviously impact my activities, including my interests in sports, travel, or any other pursuits I wish to engage in during my days.” (ID: 8H) |
| - - Fear of the Disease Limits Travelling (PwMS) | - “Traveling has always been very important for my quality of life, but now I can no longer do it. I try to compensate for this missing aspect of my life in other ways.” (ID: 9P) - “Also, trying to make plans is challenging. I love to travel; I’ve always traveled. Since May, I haven’t traveled because I decided to go during Easter (since I can’t live without traveling). But every day, I think about it and haven’t even checked in or finished paying for everything yet because I worry, ‘What if I feel unwell the day before?’ A year ago, I wouldn’t have thought about this at all. I could have been sick a year ago, even without the disease, but now it’s a constant concern. I make plans, but if…” (ID: 1P) - “The ability to travel is currently quite limited. It’s not about big trips; it’s about the simple act of moving from one neighborhood to another.” (ID: 11P) |
| - - Limitation in Playing Sports (PwMS) | - “When I stopped physical activity, following the doctors' advice, I was able to recover and continue. This helped me a lot. However, over the years, as my condition worsened and other issues arose, it became limiting.” (ID: 3P) - “Now that I do less sport because of the illness, there’s almost nothing left.” (ID: 3P) - “I used to do a lot of sports, but now I don’t do anything anymore.” (ID: 13P) |
| Work |  |
| - Work Difficulties Due to MS Symptoms (PwMS; HP) |  |
| - - Work Difficulties Due to Cognitive Impairments (PwMS; HP) | - “I’m starting to lose my memory, and this affects my work a lot. So, paradoxically, my biggest issue isn’t so much my difficulty walking, but my inability to remember. It clearly becomes more important based on what the person's priorities are at that moment, right?” (ID: 11H) - “Yes, definitely [MS cognitive symptoms affect QoL], both in my work and personal life. Sometimes I forget things that were said to me, and I struggle to follow conversations until the end. I lose track and realize I’ve missed part of the dialogue. I don’t know, I was a bit absent at times.” (ID: 7P) - “Concentration is something I’ve noticed... I’m lacking it too, and maybe that has affected my work.” (ID: 6P) |
| - - Work Difficulties Due to Visual Impairments (PwMS) | - “I work a lot on the computer, always in front of a screen, and as the day goes on, I find I need to take off and put on my glasses, which don’t really help anymore. My eyes get more tired because I’m straining one eye more than the other.” (ID: 1P) |
| - - Work Difficulties Due to Invisible Symptoms (HP) | - “So, honestly, when we talk about the impact on one’s life, social interactions, and work, fatigue—meaning exhaustion and burnout—becomes much more important to consider.” (ID: 17H) - “However, they see issues like urinary incontinence and urgency as more critical problems that need addressing because they create significant difficulties socially and at work.” (ID: 5H) - “People, even if they have little, may not show visible signs of their struggles. There’s an entire invisible part that is sometimes seen as a lack of motivation, whether at work, in personal beliefs, or even within the family.” (ID: 8H) - “This [the increased time needed to complete tasks] has a huge impact, in my opinion, and often relates to a series of invisible aspects that are the hardest to communicate, whether in work, family, or parenting.” (ID: 8H) |
| - Impact of Changes in Work Activities Due to MS (PwMS; HP) | - “I love my job dearly, and unfortunately, I can’t work in the field anymore. However, thanks to remote work, I’m still able to continue my activities.” (ID: 9P) - “My job is very important to me. I’m a pediatric nurse, and in the early years, when I was thriving, I was able to do the work I loved—work I was probably meant to do. However, when I started interferon therapy about 15 years ago—which was the only option at the time—I had to give up my previous duties. The occupational health service reassigned me to lighter tasks. From a work perspective, I was well-protected, but… I cried for ten years when I talked about this. It wasn’t a choice; it was something I had to endure.” (ID: 12P) |
| - Burden of Medical Leave Requirements (PwMS; HP) | - “Patients often face significant burdens in justifying their medical needs, such as physiotherapy or hospital visits, to their workplace due to insufficient accommodations. This constant need to explain their illness and treatment disrupts their work-life balance and is a major challenge” (ID: 15H). - "I forgo rehabilitation because I have used up all my vacation days and can no longer afford it," and many others face similar daily challenges. Another frequent issue is the need to continually justify one's condition to the workplace. (ID: 15H). - “Facilitator: Do you think rehabilitation has had a negative impact on your illness and quality of life? - PwMS: Yes, a bit, definitely. The need to organize everything is challenging. I’m an executive secretary, so when I have to be absent, it creates chaos. I try to manage everything remotely; for instance, when I’m getting an infusion, I still attend meetings and make calls if my boss contacts me. However, having to be away still causes issues.” (ID: 7P) |
| - Workplace Discrimination and Prejudice (PwMS) | - “Since my illness, I've faced problems at work. They played nasty tricks on me, and after I sent in my certification under Law 104, they reduced my salary right away. They created endless difficulties for me. I also found it really hard during job interviews. I focused on positions that accepted protected categories. I had tried applying to jobs that didn't specifically seek them, but I mentioned my situation because I had to attend treatments and take time off. I couldn't start off with a lie, so when I applied to jobs that didn't require protected status, I never got called back. I only got interview offers for protected category positions, but every time they asked me what illness I had. They shouldn’t ask for privacy reasons, but they did anyway. I went for an interview in Milan; I thought it went really well, and the interviewer seemed enthusiastic. But the last question was, 'Why do you have this disability?' I explained, and after that, I never heard back from them. They didn’t even inform me that I wasn’t moving forward in the process. This happened repeatedly, so after that experience, I stopped disclosing my illness.” (ID: 7P) - “I’ve also experienced mobbing like [name of another participant], and my physical condition worsened during that time in my life.” (ID: 2P) - “In fact, I was extremely worried when it came time to sign my third-year contract before moving to a permanent position.” (ID: 14P) |
| Finances |  |
| - Financial Impact of Disease Management (HP; PwMS) |  |
| - - Formal Caregiver Costs (PwMS) | - “I’d like to address the economic aspects of assistance. While the costs associated with medical tests can be significant, having exemptions alleviates some of that burden. However, for those on minimal pensions who require additional help, the financial strain can be overwhelming. Even with available support, like transportation aid, not everyone can access the necessary care. Individuals without family support may struggle to receive proper assistance and could feel abandoned. This issue is crucial to consider, as economic factors greatly impact access to care.” (ID: 9P) |
| - - Private Health Assistance Costs (PwMS; HP) | - “I believe that those providing your care should handle 90% of the support, rather than relying on associations. Hospitals should take on this responsibility, as it has economic implications; ultimately, people may have to seek help elsewhere.” (ID: 1P) - “Often, to access ongoing rehabilitation, individuals must turn to private services, which can be quite expensive. This financial burden is a major concern.” (ID: 2H) |
| - - Driving License Renewal Costs (PwMS) | - “Renewing my driver's license every two years is also a significant financial burden.” (ID: 7P) |
| - - Formula Milk Costs (HP) | - “It has a significant impact, including an economic one. Although it's now possible to request assistance for formula milk in cases of need, this is still an important issue.” (ID: 5H) |
| - Financial Impact of Work-Related Issues (PwMS; HP) |  |
| - - Financial Risk of Freelancing (PwMS) | - "I am self-employed, so when I take time off, I don't receive payment. This concern has been present since the day I was hospitalized. With two children to support, I inquired about the length of my hospital stay because I knew these days would be counted as sick leave. Whenever I feel unwell, I make every effort to continue working because the financial impact is significant and a constant worry." (ID: 1P) |
| - - Risk of Job Loss or Demotion (HP) | - “From an economic perspective, there is a notable impact, especially considering the type of work a person can do. For example, if someone is a musician, an artisan, or a top manager, the implications vary. There’s uncertainty about whether to communicate the diagnosis in the workplace, as it might lead to demotion, which also brings financial concerns. If I’m a shop assistant in a small store and cannot stand all day, I risk being fired, significantly affecting my financial situation.” (ID: 2H) - “If I can't secure remote working despite the ability to do my job from home, and I’m already exhausted before reaching the office, it becomes more difficult to maintain my job and my previous standard of living.” (ID: 2H) |
