# Supplementary material for: Quality of life domains revised by people with multiple sclerosis and healthcare professionals for adaptive measure development
Source: PLoS One. 2026 Jun 11;21(6):e0349034. doi: 10.1371/journal.pone.0349034 (PMC13257964; doi:10.1371/journal.pone.0349034)
Supplement: Attachment — Submitted filename: download_Reviewed.pdf [file pone.0349034.s007.pdf]

PLOS ONE

Quality of life domains revised by people with multiple sclerosis and healthcare professionals for adaptive measure development

--Manuscript Draft--

**Manuscript Number:**	PONE-D-25-43699
**Article Type:**	Research Article
**Full Title:**	Quality of life domains revised by people with multiple sclerosis and healthcare professionals for adaptive measure development
**Short Title:**	Revising QoL Domains in Multiple Sclerosis: Patient and Clinician Insights for Adaptive Measure Development
**Corresponding Author:**	Andrea Giordano<br>Fondazione IRCCS Istituto Neurologico Carlo Besta<br>Milan, ITALY
**Keywords:**	Health related quality of life; quality of life; qualitative study; multiple sclerosis; outcome measures; computerized adaptive testing.
**Abstract:**	Background. Multiple sclerosis (MS) significantly affects individuals' health-related quality of life (HRQoL). Ideally, HRQoL assessment tools for MS should be patient-centered, ensuring that each item meaningfully contributes to measurement precision. The present study has two main objectives: (1) a review of the literature to identify key HRQoL domains by examining existing instruments; and (2) confirmation and integration of HRQoL domains based on stakeholder input.<br>Methods. Findings from the literature review were discussed in four focus group meetings (FGMs) with key stakeholders, including people with MS (PwMS) and healthcare professionals (HPs). Participants were purposively sampled to ensure diversity in geographic location, gender, education, and (for PwMS) disease severity. FGMs were audio-recorded, transcribed verbatim, and analyzed using qualitative content analysis by two independent researchers.<br>Results. Out of 5190 references, 308 records related to 9 instruments were included in the literature review. The main HRQoL domains were then extracted and discussed with the steering committee, and informed the FGM grids. Qualitative analysis identified two overarching themes: (1) HRQoL Domains; and (2) Factors influencing HRQoL. The results validated the HRQoL domains identified in literature and provided deeper insights into their complexity.<br>Conclusion. This study confirmed and added granularity to the existing HRQoL domains relevant to PwMS, identifying key areas for inclusion in future research. The findings contribute to the development of a more comprehensive, patient-centered HRQoL assessment tool and also highlight important factors influencing HRQoL, which could inform targeted interventions to enhance quality of life in PwMS.
**Order of Authors:**	Ambra Mara Giovannetti
	Andrea Giordano
	Giulia Di Domenico
	Giampaolo Brichetto
	Eleonora Cocco
	Edoardo Donarelli
	Rachele Paolucci
	Alessandra Solari
	Silvia Testa
	Rosalba Rosato
**Additional Information:**	
**Question**	**Response**

Powered by Editorial Manager® and ProduXion Manager® from Aries Systems Corporation

**Financial Disclosure**<br><br>Enter a financial disclosure statement that describes the sources of funding for the work included in this submission. Review the submission guidelines for detailed requirements. View published research articles from *PLOS ONE* for specific examples.<br><br>This statement is required for submission and **will appear in the published article** if the submission is accepted. Please make sure it is accurate.<br><br>**Funded studies**<br>Enter a statement with the following details:<br>• Initials of the authors who received each award<br>• Grant numbers awarded to each author<br>• The full name of each funder<br>• URL of each funder website<br>• Did the sponsors or funders play any role in the study design, data collection and analysis, decision to publish, or preparation of the manuscript?<br><br>Did you receive funding for this work?	Yes
Please add funding details.<br>as follow-up to "**Financial Disclosure**<br><br>Enter a financial disclosure statement that describes the sources of funding for the work included in this submission. Review the submission guidelines for detailed requirements. View published research articles from *PLOS ONE* for specific examples.<br><br>This statement is required for submission and **will appear in the published article** if the submission is accepted. Please make sure it is accurate.	This study is supported by FISM - Fondazione Italiana Sclerosi Multipla – grant no. FISM 2022/PR-Multi/001 to Rosalba Rosato), by the Italian Ministry of University and Research (MUR), PRIN 2022 (Project No. 2022CA2FK8), and partially supported by the Italian Ministry of Health (RRC). The funding sources had no role in study design, data collection, data analysis, data interpretation or report writing.

Powered by Editorial Manager® and ProduXion Manager® from Aries Systems Corporation

**Funded studies**<br>Enter a statement with the following details:<br>• Initials of the authors who received each award<br>• Grant numbers awarded to each author<br>• The full name of each funder<br>• URL of each funder website<br>• Did the sponsors or funders play any role in the study design, data collection and analysis, decision to publish, or preparation of the manuscript?<br><br>Did you receive funding for this work?"	
Please select the country of your main research funder (please select carefully as in some cases this is used in fee calculation).<br>as follow-up to "**Financial Disclosure**<br><br>Enter a financial disclosure statement that describes the sources of funding for the work included in this submission. Review the submission guidelines for detailed requirements. View published research articles from *PLOS ONE* for specific examples.<br><br>This statement is required for submission and **will appear in the published article** if the submission is accepted. Please make sure it is accurate.<br><br>**Funded studies**<br>Enter a statement with the following details:<br>• Initials of the authors who received each award<br>• Grant numbers awarded to each author<br>• The full name of each funder<br>• URL of each funder website<br>• Did the sponsors or funders play any role in the study design, data collection and analysis, decision to publish, or preparation of the manuscript?<br><br>Did you receive funding for this work?"	ITALY - IT
**Competing Interests**	None

Use the instructions below to enter a competing interest statement for this submission. On behalf of all authors, disclose any competing interests that could be perceived to bias this work—acknowledging all financial support and any other relevant financial or non-financial competing interests.<br><br>This statement is required for submission and **will appear in the published article** if the submission is accepted. Please make sure it is accurate and that any funding sources listed in your Funding Information later in the submission form are also declared in your Financial Disclosure statement.<br><br>View published research articles from *PLOS ONE* for specific examples.<br><br>**NO authors have competing interests**<br><br>Enter: *The authors have declared that no competing interests exist.*<br><br>**Authors with competing interests**<br><br>Enter competing interest details beginning with this statement:<br><br>*I have read the journal's policy and the authors of this manuscript have the following competing interests: [insert competing interests here]*<br><br>* typeset	
**Ethics Statement**<br><br>Enter an ethics statement for this submission. This statement is required if the study involved:<br><br>• Human participants<br>• Human specimens or tissue<br>• Vertebrate animals or cephalopods<br>• Vertebrate embryos or tissues	The protocol received ethical clearance from the ethics committees of the coordinating centres and the three enrolling centres: UNITO (21/11/2023, internal ref: 0636827); FINCB (30/10/2023, internal ref: CET 29/23); AISM (19/12/2024, internal ref: 791/2024); UNICA (11/03/2025, internal ref: 19/25). All participants gave written informed consent.

• Field research<br><br>Write "N/A" if the submission does not require an ethics statement.<br><br>General guidance is provided below. Consult the submission guidelines for detailed instructions. **Make sure that all information entered here is included in the Methods section of the manuscript.**	

**Format for specific study types**<br><br>**Human Subject Research (involving human participants and/or tissue)**<br>• Give the name of the institutional review board or ethics committee that approved the study<br>• Include the approval number and/or a statement indicating approval of this research<br>• Indicate the form of consent obtained (written/oral) or the reason that consent was not obtained (e.g. the data were analyzed anonymously)<br><br>**Animal Research (involving vertebrate animals, embryos or tissues)**<br>• Provide the name of the Institutional Animal Care and Use Committee (IACUC) or other relevant ethics board that reviewed the study protocol, and indicate whether they approved this research or granted a formal waiver of ethical approval<br>• Include an approval number if one was obtained<br>• If the study involved *non-human primates*, add *additional details* about animal welfare and steps taken to ameliorate suffering<br>• If anesthesia, euthanasia, or any kind of animal sacrifice is part of the study, include briefly which substances and/or methods were applied<br><br>**Field Research**<br><br>Include the following details if this study involves the collection of plant, animal, or other materials from a natural setting:<br>• Field permit number<br>• Name of the institution or relevant body that granted permission	
**Data Availability**<br><br>Authors are required to make all data underlying the findings described fully available, without restriction, and from the time of publication. PLOS allows rare exceptions to address legal and ethical concerns. See the PLOS Data Policy and FAQ for detailed information.	Yes - all data are fully available without restriction

A Data Availability Statement describing where the data can be found is required at submission. Your answers to this question constitute the Data Availability Statement and **will be published in the article**, if accepted.<br><br>**Important:** Stating 'data available on request from the author' is not sufficient. If your data are only available upon request, select 'No' for the first question and explain your exceptional situation in the text box.<br><br>Do the authors confirm that all data underlying the findings described in their manuscript are fully available without restriction?	
**Describe where the data may be found in full sentences. If you are copying our sample text, replace any instances of XXX with the appropriate details.**<br><br>• If the data are **held or will be held in a public repository**, include URLs, accession numbers or DOIs. If this information will only be available after acceptance, indicate this by ticking the box below. For example: *All XXX files are available from the XXX database (accession number(s) XXX, XXX.)*.<br>• If the data are all contained **within the manuscript and/or Supporting Information files**, enter the following: *All relevant data are within the manuscript and its Supporting Information files.*<br>• If neither of these applies but you are able to provide **details of access elsewhere**, with or without limitations, please do so. For example:<br><br>*Data cannot be shared publicly because of [XXX]. Data are available from the XXX Institutional Data Access / Ethics Committee (contact via XXX) for researchers who meet the criteria for access to confidential data.*<br><br>*The data underlying the results presented in the study are available from (include the name of the third party*	All relevant data are within the manuscript and its Supporting Information files.

and contact information or URL).<br>• This text is appropriate if the data are owned by a third party and authors do not have permission to share the data.<br><br>* typeset	
Additional data availability information:	

Powered by Editorial Manager® and ProduXion Manager® from Aries Systems Corporation

Manuscript Click here to access/download;Manuscript;Main Document.docx

Quality of life domains revised by people with multiple sclerosis and healthcare professionals for adaptive measure development

Ambra Mara Giovannetti[1], Andrea Giordano[2,3*], Giulia Di Domenico[1], Giampaolo Brichetto[4,5], Eleonora Cocco[6,7], Edoardo Donarelli[1], Rachele Paolucci[8], Alessandra Solari[3], Silvia Testa[9], Rosalba Rosato[1], on behalf of the INITIALISE project^

1. Department of Psychology, University of Turin, Turin, Italy. ORCID: https://orcid.org/0000-0002-7496-6727. ORCID: https://orcid.org/0000-0002-9769-4576. ORCID: https://orcid.org/0000-0002-4921-374X
2. Neurology, Public Health and Disability Unit, Fondazione IRCCS Istituto Neurologico Carlo Besta, Milan, Italy. ORCID: https://orcid.org/0000-0003-4466-9788.
3. Unit of Neuroepidemiology, Fondazione IRRCS Istituto Neurologico Carlo Besta, Milan, Italy. ORCID: https://orcid.org/0000-0001-9930-7579.
4. Scientific Research Area, Italian Multiple Sclerosis Foundation (FISM), Genoa, Italy. ORCID: https://orcid.org/0000-0003-2026-3572.
5. AISM Rehabilitation Service, Italian Multiple Sclerosis Society, Genoa, Italy.
6. Department of Medical Science and Public Health, University of Cagliari, Cagliari, Italy. ORCID: https://orcid.org/0000-0002-3878-8820.
7. Multiple Sclerosis Center, ASL Cagliari, Cagliari, Italy.
8. Italian Multiple Sclerosis Society (AISM)
9. Department of Human and Social Sciences, University of Aosta Valley, Aosta, Italy. ORCID: https://orcid.org/0000-0002-8732-4790.

*andrea.giordano@istituto-besta.it

^Membership of the INITIALISE project is provided in the Acknowledgements.

Short title: Revising HRQoL Domains in Multiple Sclerosis: Patient and Clinician Insights for Adaptive Measure Development

Keywords: Health related quality of life; quality of life; qualitative study; multiple sclerosis; outcome measures; computerized adaptive testing.

Abstract

Background. Multiple sclerosis (MS) significantly affects individuals' health-related quality of life (HRQoL). Ideally, HRQoL assessment tools for MS should be patient-centered, ensuring that each item meaningfully contributes to measurement precision. The present study has two main objectives: (1) a review of the literature to identify key HRQoL domains by examining existing instruments; and (2) confirmation and integration of HRQoL domains based on stakeholder input.

Methods. Findings from the literature review were discussed in four focus group meetings (FGMs) with key stakeholders, including people with MS (PwMS) and healthcare professionals (HPs). Participants were purposively sampled to ensure diversity in geographic location, gender, education, and (for PwMS) disease severity. FGMs were audio-recorded, transcribed verbatim, and analyzed using qualitative content analysis by two independent researchers.

Results. Out of 5190 references, 308 records related to 9 instruments were included in the literature review. The main HRQoL domains were then extracted and discussed with the steering committee, and informed the FGM grids. Qualitative analysis identified two overarching themes: (1) HRQoL Domains; and (2) Factors influencing HRQoL. The results validated the HRQoL domains identified in literature and provided deeper insights into their complexity.

Conclusion. This study confirmed and added granularity to the existing HRQoL domains relevant to PwMS, identifying key areas for inclusion in future research. The findings contribute to the development of a more comprehensive, patient-centered HRQoL assessment tool and also highlight important factors influencing HRQoL, which could inform targeted interventions to enhance quality of life in PwMS.

Introduction

Multiple sclerosis (MS) is a chronic demyelinating disease of the central nervous system, the most frequent cause of neurological disability in young adults, characterized by an unpredictable pathological process and progressive degeneration [1-3]. Clinical onset is generally in the most productive age of life, usually between 20 and 40 years [4]. The disease has a pervasive effect on physical, cognitive and psychological functioning and well-being [5-7]. Fatigue, pain, depression, and cognitive dysfunction are some of the multiple symptoms impaired by MS that can affect various areas of life for people with MS (PwMS), such as the ability to work and/or perform activities of daily living, but are difficult for healthcare providers to quantify [8].

In recent years, there has been a growing awareness among clinical authorities and regulators to use outcome measures that are meaningful to patients themselves, potentially expressed through the patient voice, when evaluating new treatments. In light of this medical trend, health-related quality of life (HRQoL) measurement instruments have gradually gained more attention [9]. Leidy et al. defined HRQoL as "the subjective perception of the impact of disease and treatment across the physical, psychological, social, and well-being" [10]. Studying HRQoL means exploring how patients experience and live with the manifestations of their disease in their daily lives, adding a new perspective to the clinician-based evaluation of the presence and severity of symptoms [11,12]. This can be particularly important for people living with chronic diseases for which there is no cure and whose treatment is often lengthy and polytherapeutic [10]. The aim of HRQoL instruments is to provide a more complete and personalized measure of the impact of the disease, improving the detection of aspects that are more likely to go unnoticed. This helps clinicians to recognize patients' priorities and facilitates communication between clinician and patient, thus promoting shared decision-making [9]. However, these tools are rarely used in frontline MS services and integrated into clinical practice [13]. This is due to potential logistical and financial

issues associated with their administration, processing, interpretation, storage, and retrieval [14,15]. Other challenges are the fixed-length, paper-pencil format of most HRQoL instruments, as opposed to the need to collect data in a timely and understandable manner to reduce respondent burden and missing data, increasing measurement accuracy and the opportunity to share findings during the medical encounter.

Computer-Adaptive Tests (CAT) [16], delivered via a digital questionnaire that dynamically selects only the most relevant items based on patient responses, have been applied to HRQoL assessment [17], as their tailored format is thought to reduce the impact of cognitive fatigue while increasing acceptability and relevance for PwMS [18,19]. The growing interest in CAT instruments in HRQoL research has led to the development of a range of CAT instruments by the Patient-Reported Outcomes Measurement Information System (PROMIS) supported by the National Institute of Health [20], which currently offers 3 to 10 items for domains such as fatigue, physical functioning, pain, depression, anxiety, and social functioning (www.nihpromis.org). For the assessment of multidimensional constructs with correlated dimensions, the Multidimensional Computer Adaptive Testing (MCAT) approach is more appropriate, but, as far as we know, there is few evidence of HRQoL instruments using such a methodology to fixed-length HRQOL questionnaires, including the MS domain [21-24]. To address this gap, we took a comprehensive approach based on a literature review and informed by the patient voice to identify relevant HRQoL dimensions that ensure both content validity and clinical utility of the assessment [20,25,26].

A systematic review of the literature provides a useful basis for identifying commonly assessed HRQoL domains in MS. However, the literature alone cannot fully capture the lived experiences and needs of PwMS. Additional conceptual analysis is essential to explore nuanced patient experiences and clarify overlapping constructs, such as fatigue and depression or physical

disability and social isolation. This process supports the development of a coherent and clinically meaningful framework that guides the structure and content of the HRQoL assessment.

There is evidence of the importance of including patients' perspectives and insights in patient-reported outcomes (PROs), as this can help to identify potential issues related to domain coverage and/or inadequate operationalization [27-39]. This patient-centered approach ensures the inclusion of domains that may be underrepresented in existing instruments but are highly relevant to people living with the disease and increases the content validity of the questionnaire [40,41]. However, in the MS context, the perspective of PwMS have often been overlooked in the development of existing PROs. As a result, important information about what truly matters to them is missing [42,43].

A well-defined domain structure of HRQoL in MS may be useful for several future research directions, including the development of psychometrically sound item banks that allow for accurate, efficient, and individualized assessment, e.g., using the MCAT approach [44,45], as well as studies aimed at capturing and ranking the priorities that patients assign to these domains.

The present study has two main objectives: 1) to conduct a systematic literature review to identify the most relevant HRQoL domains in MS; 2) to discuss the identified HRQoL domains with PwMS and eventually supplement them with additional relevant domains.

Materials and Methods

The project is based on the participatory governance model proposed by the Multiact Guidelines which involves the establishment of a working group called the Engagement Coordination Team (ECT) [46]. This is a multidisciplinary team representing key stakeholders for the study (researchers, clinicians, experts in the Multiact model, and importantly, patients – in our case, PwMS) who collaborate in the research to capture the disease experience and translate it into

scientific knowledge, with the aim of improving, recalibrating, and potentially modifying the research project being undertaken.

The project is co-coordinated by the University of Turin (UNITO) and the Fondazione IRCCS Istituto Neurologico Carlo Besta (FINCB), Milan, and run in three Italian MS centres: FINCB; the Italian MS rehabilitation centre (AISM) and the Scientific Research Area (FISM), Genoa; and the ASL Cagliari/University of Cagliari (UNICA), Cagliari. The study was carried out in accordance with the Declaration of Helsinki recommendations. The protocol received ethical clearance from the ethics committees of the coordinating centres and the three enrolling centres: UNITO (21/11/2023, internal ref: 0636827); FINCB (30/10/2023, internal ref: CET 29/23); AISM (19/12/2024, internal ref: 791/2024); UNICA (11/03/2025, internal ref: 19/25). All participants gave written informed consent.

Systematic review

We adhered to the PRISMA guidelines for the systematic review and reporting of outcome measurement instruments [47]. To identify key HRQoL domains, we included studies that utilized a multidimensional, MS-specific HRQoL instrument in adults with MS. Studies that employed the instrument solely as an outcome measure (e.g., clinical trials) or for the validation of other instruments were excluded.

We developed a search strategy for PubMed (inception to September 2, 2023; no language restrictions) and adapted it for EMBASE, PsycINFO, CINAHL, and the online database for existing PROs (www.proqolid.org). Since we aimed to collect all MS-specific instruments that have been used to measure HRQoL in adults with MS, we used the relevant search strategy structure developed by the COSMIN initiative [48]. To identify additional studies, we also reviewed published systematic reviews and the reference lists of the selected articles. Search terms included

‘health-related quality of life’, ‘quality of life’, ‘health profiles’, ‘well-being’, and ‘multiple sclerosis’. The search strategy is detailed in S1 File.

Two reviewers (AG and ED) conducted a pilot test with 10 articles to refine the eligibility criteria. Subsequently, the same reviewers independently screened the titles and abstracts for eligibility. Full-text articles of selected studies were then independently assessed by the reviewers. We included studies that reported primary research on HRQoL in MS, excluding systematic reviews, protocols, clinical guidelines, conference proceedings, and editorials.

Data extraction was performed by two reviewers (AG and ED) and verified by a third reviewer (RR) using a bespoke electronic form.

We extracted key HRQoL domains from the MS-specific HRQoL instruments included in the review and mapped them using the HRQoL domains proposed by Leidy et al. [10]. All (sub)domains included in at least one of the tools cited in the selected literature were considered.

For each (sub)domain, we assessed item-related content to ensure alignment with its conceptual framework and to refine its characterization within the theoretical model. The identified (sub)domains were then illustrated in a figure, with revisions made to names and redundant (sub)domains consolidated.

The provisional HRQoL figure was reviewed during a steering committee (SC) meeting and revised based on feedback from board members. Subsequently, the figure was discussed with focus group facilitators to develop focus group meeting (FGM) guides and assist participants in understanding the structure of the domains.

Qualitative study

The relevance of the key HRQoL domains identified in the literature was assessed, and additional relevant domains were explored through FGMs with PwMS and healthcare

professionals (HPs). Two FGMs were conducted for each group of stakeholders. FGMs were selected because they facilitate the exploration of multiple perspectives simultaneously, while encouraging interaction, brainstorming, and the elaboration of ideas.

AMG and GDD devised the FGM guides (S2 File) with input from the SC.

Meeting with the ECT

Ten days before the meeting, ECT members received the two FGM guides in order to read and comment on them individually. Both consist of five sections which investigated the impact of MS on HRQOL. Field notes were taken and integrated accordingly to the issues identified to modify the guides.

FGM enrolment procedure

Investigators at each participating centre identified potentially eligible PwMS from their database and invited them to participate in the study. Eligible MS HPs were identified by the SC from the participating centres and subsequently invited to join. FGM participants were selected through purposive sampling to ensure a broad range of perspectives and opinions. Participants were drawn from two geographic regions of Italy (North and South) and varied in terms of age, gender, education, and, for PwMS, neurological impairment (based on the Expanded Disability Status Scale, EDSS [49]), and years since MS diagnosis.

PwMS were included if they were at least 18 years of age, fluent in Italian, and provided written informed consent. Exclusion criteria for PwMS included severe cognitive impairment, as assessed by the referring neurologist, and an inability to communicate effectively.

HPs were eligible if they had experience in the care of pwMS (i.e., working in clinical or rehabilitative specialized settings and actively involved in the assistance, care, and support of PwMS), were fluent in Italian, and provided written informed consent.

FGM conduction

Each FGM was designed to include 6 to 10 participants, alongside two facilitators (AMG and GDD). To enhance feasibility and participation, the FGMs were conducted online via Zoom. This approach was intended to address potential mobility issues faced by PwMS, optimize participants' time, and minimize scheduling conflicts, thereby maximizing the likelihood of successful recruitment.

The principal facilitator (AMG) began by explaining the purpose of the meeting and asking participants to introduce themselves. She then introduced each topic sequentially and guided the discussion. After covering all pre-specified topics, the facilitator summarized the key points and invited additional comments. The co-facilitator (GDD) took notes and managed the audio recording. The FGMs were audio-recorded and fully transcribed. Within two weeks of the FGMs, participants received a report summarizing the meeting for review (respondent validation).

Analyses

Content analysis was used to code the FGM transcripts, employing a combination of deductive and inductive approaches. The deductive process, informed by findings from the literature review (existing HRQoL domains), applied category labels consistent with the literature when appropriate. Simultaneously, the inductive approach enabled the identification of novel themes that emerged directly from the data. This dual approach began with a deductive framework to structure the initial analysis while incorporating inductive coding to capture insights not anticipated in the literature, ensuring a more comprehensive and nuanced understanding of the data.

A line-by-line coding method ensured thorough and detailed analysis. A three-step coding scheme was implemented: Two researchers (AMG and GDD) analyzed the transcripts independently during steps 1 and 2, and collaboratively in step 3. In the first step, initial codes were extracted directly from participants' written responses. The second step involved data aggregation, with each

researcher independently generating labels for codes and categories. In the third step, the researchers discussed and refined the independently generated codes and categories [50]. The Consolidated Criteria for Reporting Qualitative Research (COREQ) [51] guided the presentation of findings, leading to the creation of a detailed report. The adherence to the COREQ checklist is documented in the S3 File. The results from this phase were reviewed by the SC and the ECT, which determined the final set of HRQoL domains and updated the HRQoL list accordingly.

Results

Systematic review

We identified 5190 references and, with the initial screening, 4655 records were excluded. Five hundred thirty-five full-text references were assessed for eligibility and, of those, 227 were excluded because only abstract was available (n=149), the study was not related to a multi-dimensional HRQoL instrument (n=42), were reviews (n=27), included wrong populations (n=9). We included 308 references related to 9 instruments: Multiple Sclerosis Quality of Life-54 items (MSQoL-54) [52] (n=134), Multiple Sclerosis Impact Scale (MSIS-29) [53] (n=59), Functional Assessement of Multiple Sclerosis (FAMS) [54] (n=49), Multiple Sclerosis International Quality of Life questionnaire (MusiQoL) [55] (n=45), Hamburg Quality of Life questionnaire in Multiple Sclerosis (HAQUAMS) [56] (n=17), Patient Reported outcome indices for Multiple Sclerosis (PRIMUS) [57] (n=7), Short Quality of Life scale (SQOL) [58] (n=4), RAYS [59] (n=3), Functional Index for Living with Multiple Sclerosis (FILMS) [60] (n=1). The PRISMA flow diagram is depicted in S4 File. The relevant domains were then extracted from these HRQOL instruments.

Conceptual work and HRQoL domains mapping

After the SC meeting, a few symptoms (i.e., sensory impairments, balance and coordination, swallowing and spasticity) were added to the 'physical domain'. The 'psychological domain' was slightly revised by splitting cognition and emotion subdomains, and deleting a few redundant subdomains (i.e., general contentment, mood).

After the meeting with FGM facilitators, subdomains overlapping the 'psychological' and the 'social' domains were slightly revised. Specifically, social well-being was considered redundant and, as such, was included in social support and social relationships, while self-perception in social settings was better specified.

All these revisions were integrated into the domain mapping (S5 File), resulting in the graphical representation shown in Figure 1.

The definitions of the 'physical', 'psychological', and 'social' domains were prepared and included in the FGM guides, each accompanied by a list of three representative items.

[Insert Figure 1 about here]

Qualitative study

Meeting with the ECT for finalizing the FGM guides

On February 2, 2024, a 2-hour online meeting was conducted with six PwMS (median age 35 years [range 31-64], 67% women, 84% single, all with college education and full-time employment, 67% with relapsing-remitting MS, median EDSS 2.0 [range 1.0-6.5], median years since MS diagnosis 11.5 [range 2-19]), and four INITIALISE researchers. The FGM guide was generally considered clear. Key modifications focused on rephrasing questions to better capture the impact of MS on HRQoL, particularly by refining how treatment and daily functioning were framed. Notable changes included adding 'rehabilitation' to the definition of MS treatment and clarifying the distinction between activities of daily living (ADL) and instrumental activities of daily living (IADL). Participants

appreciated the inclusion of questions addressing both negative and positive influences on HRQoL. The HPs guide was adapted from the patient version. The revised guides are detailed in the S2 File.

FGMs participants and setting

Between January 1, 2024 and March 31, 2024 we enrolled PwMS and HPs, with recruitment initiated simultaneously across all study centers. Four online FGMs were conducted in April 2024, two with PwMS and two with HPs. The average duration of the FGMs was 144 minutes (SD 9.9) with PwMS, and 132 minutes (SD 14.1) with HPs. Among those contacted and enrolled, five PwMS did not participate in the FGM: two due to medical reasons and three due to unforeseen work commitments. Additionally, two enrolled HPs were unable to attend, one due to a medical condition and the other due to technical difficulties. The PwMS sample was equally distributed by gender, with a median age of 52.5 years (range 36–64). The median EDSS score was 4.0 (range 1.5–9.0), indicating a broad range of disability levels. Half of the sample had a relapsing-remitting course, the other half had a progressive form, with a median disease duration of 17 years (range 5 months-42 years).

Most HPs were women (83%), with a median age of 50 years (range 30–65). HPs had a mean of 19.6 years of experience in the MS field (range 3–35 years), and followed a median of 100 PwMS in the previous six months (range 12–600). They were employed across various settings, including MS centres, rehabilitation centres, and AISM facilities, in roles such as neurologists, nurses, physiotherapists, psychologists, and social workers.

FGMs results

Qualitative data collected during the four FGMs were analysed and organized into two overarching themes: 1) HRQoL Domains; 2) Factors influencing HRQoL. The HRQoL Domains theme is composed of three subthemes: 1.1 Physical Domain; 1.2 Psychological Domain; 1.3 Social Domain.

The factors influencing HRQoL theme is composed of five subthemes: 2.1 Personal Factors; 2.2 Health-Related Factors; 2.3 Life-Related Factors; 2.4 Relational Factors; 2.5 Contextual Factors. This section presents each theme along with its main categories and illustrative quotes. The quotes include participants' IDs in brackets: (ID: number, 'P' or 'H'), where 'P' represents PwMS and 'H' denotes HPs. A complete list of categories, subcategories, and quotes is available in the audit trail (see S6 File).

1) HRQoL Domains

Table 1 highlights the key determinants of HRQoL across three domains, incorporating perspectives from both PwMS and HPs: Physical, Psychological, and Social. The FGM results confirmed all domains identified in the existing literature, but offered greater detail in terms of categories and subcategories within each domain.

Table 1. List of HRQoL Domains (only main categories).

Physical Domain	Psychological Domain	Social Domain
Symptoms* (PwMS; HP)	Cognitive Function* (PwMS; HP)	Support* (PwMS; HP)
ADL* (PwMS; HP)	Health Perceptions* (PwMS; HP)	Social Connectedness* (PwMS; HP)
IADL* (PwMS; HP)	Emotional Well-Being* (PwMS; HP)	Self-Perception in Social Settings* (PwMS; HP)
Impact of the DMT on the body (HP)	MS is a Stressor* (PwMS; HP)	Social Life (PwMS; HP)
		Work (PwMS; HP)
		Finances (PwMS; HP)

ADL, activities of daily living; DMT, disease-modifying therapy; HP, health professional; HRQoL, health-related quality of life; IADL, instrumental activities of daily living; MS, multiple sclerosis; PwMS, people with multiple sclerosis.
An asterisk (*) next to the category name indicates those reported in both the literature and FGMs.

Physical Domain

The Physical Domain includes symptoms (10 subcategories), ADL/IADL (two subcategories), and the impact of disease-modifying therapies (DMTs). For the full list of categories, subcategories,

and quotes see S6 FIle: tables S3, S3.1 and S3.1.1.

In the Physical Domain, determinants of HRQoL for PwMS reveal significant challenges. Key symptoms include mobility impairments, with many reporting progressive difficulties*: 'The illness makes itself felt, even though from the outside it's not so noticeable'* (ID: 8P). Pain, particularly neuropathic pain, is frequently overlooked but severely impacts daily life: *'Pain is often very disabling for PwMS'* (ID: 11H). Incontinence issues further complicate daily experiences, with one participant stating, *'I stopped going out because I couldn't control the situation'* (ID: 4P). Symptoms often limit ADL and IADL: *'I can only manage to do one thing a day'* (ID: 6P). The impact of DMTs can also influence physical function, with one HP stated, *'The type of drug absolutely changes daily life'* (ID: 14H).

Psychological Domain

Central factors affecting HRQoL in PwMS in the Psychological Domain include cognitive function, health perception (five subcategories), emotional well-being (seven subcategories), and stress related to MS. For the full list of categories, subcategories, and quotes see S6 File: tables S3, S3.2; S3.2.1). Participants reported feeling mentally slower and less sharp. One PwMS expressed this concern: *'Mentally, I feel much slower, even in terms of attention. That, for me, is what the disease has affected the most.'* (ID: 10P). HPs also noted that cognitive impairment can significantly impact a person's life: *'Sometimes, even when physical limitations are relatively minor, the person might struggle with concentration, and that can have a major impact'* (ID: 8H). Another participant reflected on initial misconceptions related to illness representation and its impact on QoL: *'Not knowing much about the disease, I immediately thought of a diagnosis leading to a wheelchair. And that was difficult to shake off as an impact, let's say'* (ID: 4P).

Finally, a recently diagnosed PwMS pointed out how MS can act as stressor: *'Certainly, [MS] is a source of stress because it is unpredictable—you don't know how things will be today or a year*

from now. You can read all you want and hear all sorts of things, but no one can tell you how it will progress.' (ID: 1P). Again, these aspects were supported also by HPs: *'The impact [of the diagnosis] is quite profound, as we know, because MS […] is one of those conditions that elicits fear and is frequently mentioned in news reports, alongside other incurable diseases. […] Thus, it is grouped with other major fears, such as cancer. Consequently, when a diagnosis is made, it signifies that 'from this moment on, my life will be forever different, and I am, in fact, among the most unfortunate people in the world'* (ID: 17H).

Social Domain

The Social Domain includes Support (two subcategories), Social Connectedness (six subcategories), Self-Perception in Social Settings (three subcategories), Social Life (four subcategories), Work (four subcategories) and Finances (two subcategories). For the full list of categories, subcategories, and quotes see S6 File: tables S3, S3.3 and S3.3.1.

Support covers the assistance patients receive and the challenges they face in supporting others, especially in parenting and relationships. The psychological burden from MS can hinder empathy and limit the ability to seek help. One participant emphasized the importance of family, expressing concerns about not being able to engage in activities with their young child as they once could, saying, *'I worry about how much I can physically give him… it's different now'* (ID: 14P).

Social Connectedness reveals the impact of invisible symptoms like fatigue and incontinence on interactions. Medical care demands, fear of disclosure, and sexual dysfunction further isolate PwMS. One participant shared: *'Fatigue limits opportunities for social interactions… I might manage to go out once a week at best'* (ID: 11P).

Self-Perception addresses stigma and feelings of vulnerability. One participant noted: *'What bothers me more is how others perceive my situation… I'm less concerned about my experience than about judgment from others'* (ID: 14P).

Social Participation highlights restrictions in family activities, social life, and leisure. A nurse commented, *'Treatments impact social life and energy levels… after a rehab session, they often need to rest, which cuts into their social time' (ID: 16H).*

Work captures the challenges PwMS face in maintaining employment and navigating professional roles. Key issues include difficulties caused by MS symptoms (e.g., cognitive fatigue), changes in work activities, the burden of medical leave, and discrimination. One participant shared: *"Yes, definitely [MS cognitive symptoms affect QoL], both in my work and personal life. Sometimes I forget things that were said to me, and I struggle to follow conversations until the end. I lose track and realize I've missed part of the dialogue. I don't know, I was a bit absent at times." (ID: 7P).*

A paediatric nurse reflected on the professional consequences of treatment: *"My job is very important to me… When I started interferon therapy about 15 years ago, I had to give up my previous duties for lighter tasks. It wasn't a choice; it was something I had to endure." (ID: 12P).*

An HP added insight into structural challenges in the workplace: *"Patients often struggle to justify their medical needs, like physiotherapy or hospital visits, due to inadequate workplace accommodations. This need to constantly explain their condition disrupts their work-life balance and is a major challenge." (ID: 15H).*

Financial concerns relate to both direct disease-management costs and the economic burden of work limitations. Participants described the financial strain of accessing ongoing rehabilitation, often requiring private services. One HP noted: *"Often, to receive continuous rehabilitation care, one must resort to private services, which can be quite costly. This financial burden is a significant concern." (ID: 2H).* A PwMS reflected on the personal cost of self-employment with MS: *"I am self-employed, so when I take time off, I don't receive payment. This concern has been present since the day I was hospitalized. With two children to support, I inquired about the length of my hospital stay because I knew these days would be counted as sick leave. Whenever I feel unwell, I make every*

effort to continue working because the financial impact is significant and a constant worry." (ID: 1P).

2) Factors influencing HRQOL

Table 2 provides an overview of the main factors influencing HRQoL across five subthemes: 2.1 Personal Factors; 2.2 Health-Related Factors; 2.3 Life-Related Factors; 2.4 Relational Factors; 2.5 Contextual Factors.

Table 2. List of Factors influencing HRQoL.

Personal Factors	Health-Related Factors	Life-Related Factors	Relational Factors	Contextual Factors
Personality (PwMS; HP)	Stages of Disease (HP)	Age-Related Stages (HP)	Personal Relationships (PwMS; HP)	Healthcare Accessibility and Organizational Capacity (HP)
Adjustment to MS/Resilience (PwMS; HP)	Discovery of Body Resources (HP)	Life Development (HP)	Quality of Support Received (PwMS; HP)	Architectural Barriers (PwMS)
Personal Empowerment (HP)	Comorbidities (HP)			Economic Conditions (HP)
Self-Management (PwMS; HP)	Type of Pharmacological Treatment (HP)			Work (PwMS; HP)
Attitudes Towards Medications (HP)				Access to Information (PwMS)

HP, health professional; HRQoL, health-related quality of life; MS, multiple sclerosis; PwMS, people with multiple sclerosis.

Personal Factors

Personal Factors significantly influence the HRQoL of PwMS and can be collected in five categories. Personality, Adjustment to MS/Resilience (three subcategories), Personal Empowerment (two subcategories), Self-Management (four subcategories), and Attitudes Towards Medications. For the full list of categories, subcategories, and quotes see S6 File: tables S4, S4.1 and S4.1.1.

Participants reported that their responses to MS were shaped by their unique personality traits. One participant stated, *"Everyone reacts based on their own character"* (ID: 13P), highlighting the idiosyncratic nature of coping mechanisms.

Adjustment to MS/Resilience was often linked to self-discovery, as participants frequently rediscovered inner strengths: *"They rediscover their resilience—something they never thought they could be"* (ID: 1H). The process of acceptance emerged as crucial for psychological stability, with participants emphasizing the importance of coming to terms with their diagnosis: *"Once I accepted it, I didn't flaunt it, but I did make it known"* (ID: 11P). While denial and avoidance were identified as barriers to adjustment, especially among newly diagnosed individuals: *"Some seem to be in denial [...] Even this avoidance can impact quality of life"* (ID: 14H). Personal values and life goals also influenced symptom perception. Cognitive symptoms, for example, were seen as more distressing than physical limitations by some, depending on their aspirations: *"Some symptoms are more significant for individuals based on their life goals and dreams"* (ID: 10H). Many participants continued to strive for self-expression despite MS: *"Even those who are very ill still have a desire to live, and [...] find ways to express themselves"* (ID: 11H).

Personal Empowerment was highlighted as another important factor influencing HRQoL. A sense of self-efficacy was crucial, as patients who managed aspects of their care or treatment were more likely to experience greater satisfaction and autonomy: *"The more they can regain a sense of self-efficacy [...], the better their quality of life can be"* (ID: 4H).

Knowledge about MS facilitated better self-management, with participants noting that an understanding of their condition helped improve their emotional adaptation: *"When patients become more knowledgeable, they can use that experience positively"* (ID: 15H).

Finally, attitudes toward pharmacological treatment was also significant. While some viewed therapy as a reassuring aspect of their future, others saw it as a constant reminder of their illness:

"Some people are happy to undergo therapy because it becomes a reassuring aspect for the future, while for others, it's [...] a constant reminder that 'I'm sick'" (ID: 11H).

Health-Related Factors

Health-Related Factors, including Stages of Disease, Discovery of Body Resources, Comorbidities, and Type of Pharmacological Treatment significantly influenced HRQoL. For the full list of categories, subcategories, and quotes see S6 File: tables S4, S4.1 and S4.1.2.

The Stage of Disease was particularly impactful, with participants noting that perceptions of the disease changed over time. As one HP explained, *"The way a person perceives their impact on the disease changes over time. As the illness progresses, both the needs and the approaches of individuals evolve"* (ID: 11H). This progression emphasized the need for flexible care strategies.

MS often led patients to rediscover bodily resources, with one participant stating, *"With the illness, people learn to discover their body and actually some new resources. This has helped them a lot"* (ID: 12H). Such discoveries fostered a sense of empowerment, enabling patients to adapt to their changing physical capacities.

Comorbidities, particularly those related to aging, added complexity to disease management. One participant noted, *"At the level of comorbidity management, this introduces a different complexity"* (ID: 8H). These additional health issues often heightened the burden of MS, complicating treatment and altering the patient experience.

Pharmacological Treatments also had a considerable impact on daily life. One participant explained how different types of treatment affected their routines: *"The type of treatment for MS can greatly affect a person's daily life. Different methods of administration—like oral medications or injections—impact daily routines in various ways"* (ID: 18H). The psychological and practical implications of these treatments were as important as their clinical effects.

Life-Related Factors

Life-Related factors, such as age, life stages, and significant events, also played a critical role in shaping the experiences of PwMS. For the full list of categories, subcategories, and quotes see S6 File: tables S4, S4.1 and S4.1.3.

Age at diagnosis was particularly influential. Younger individuals often demonstrated greater adaptability, while older ones faced more difficulty accepting the diagnosis and the resulting limitations: *"For patients diagnosed with MS at 60, 65, or even 70, the disease can be particularly hard to accept"* (ID: 7H). Younger individuals, especially those in the workforce or pursuing personal goals, faced challenges in balancing their aspirations with the limitations imposed by MS. The timing of diagnosis also affected individuals' perceptions of HRQoL. One participant noted that older adults, particularly retirees, often reported a better quality of life despite their health issues: *"Older adults, especially those who are retired and over 60, often report a better quality of life compared to younger individuals"* (ID: 4H). In contrast, younger adults experienced greater emotional strain, especially when MS disrupted their life plans: *"The impact of MS varies by age. Teenagers often experience the disease through their parents' perspective, with the parents handling much of the emotional burden. Young adults, however, may struggle more as the disease disrupts their plans and aspirations for the future"* (ID: 9H).

Life transitions, such as pregnancy, also impacted MS experiences. For some women, pregnancy provided a temporary reprieve from symptoms: *"Pregnancy can actually be a time of well-being for some women with MS"* (ID: 5H).

This phase, marked by a sense of normalcy and symptom relief, helped foster a sense of solidarity among pregnant women with MS.

Relational Factors

Relational Factors, particularly Personal Relationships (three subcategories), and Quality of Support Received (six subcategories), played a crucial role in the HRQoL of individuals with MS. For the full list of categories, subcategories, and quotes see S6 File: tables S4, S4.1 and S4.1.4.

Strong social networks were vital for emotional support and practical assistance, particularly as the disease progressed. One participant stated, *"Having strong social relationships, being able to connect and rely on each other, even if we don't talk for a while, is very important*" (ID: 4P). The quality of these relationships, especially within family, strongly influenced QoL. *"A disabled person in a loving family generally has a high perceived quality of life. They need nothing else"* (ID: 4H). The proximity of family members also impacted participants' ability to navigate MS. For some, the distance from loved ones created additional challenges: *"The separation from my children is a significant disadvantage in dealing with my disability"* (ID: 4P). This underscores the importance of family support, particularly as caregiving demands increased.

Patients also reported transformations in their social circles, with some finding new, meaningful connections while letting go of shallow relationships: *"Understanding who your true friends are is crucial; some unexpected friends emerged during tough times"* (ID: 12P). These transformations often fostered stronger bonds and deeper communication.

Support from healthcare providers and patient associations was also emphasized. One participant reflected on the importance of support from MS centres: *"I have been receiving support from AISM in Genoa since 2016, and I will always be grateful for their help"* (ID: 8P). The relationships formed with fellow patients were equally significant, with participants sharing experiences and providing mutual support: *"The friendships formed among patients are significant. This sharing has often been very positive"* (ID: 6H).

Contextual Factors

Contextual Factors, including Healthcare Accessibility and Organizational Capacity, Architectural Barriers, Economic Conditions, Work (three subcategories), and Access to Information (four subcategories) also played a significant role in shaping HRQoL. For the full list of categories, subcategories, and quotes see S6 File: tables S4, S4.1 and S4.1.5.

Many individuals with MS faced difficulties navigating the healthcare system, with fragmented care leading to unmet needs. One participant noted the challenge of managing day-to-day symptoms independently, stating that *"Basic information, such as the implications of driving with MS or insurance coverage in the event of an accident, is frequently left undisclosed*" (ID: 15H). PwMS often had to manage bureaucratic procedures and access care based on their own initiative, adding to their stress.

Architectural barriers also restricted access to public spaces and private homes, exacerbating feelings of isolation. Economic insecurity further impacted PwMS ability to manage their illness. Financial difficulties made it harder to access necessary treatments and support services, particularly for older adults. One participant highlighted the strain caused by limited resources: *"The need for physiotherapy means justifying why I have to go to the hospital for treatment once a month"* (ID: 15H).

Work-related challenges were also significant, with some individuals benefiting from protected employment opportunities, while others struggled to maintain jobs in physically demanding fields. Flexible work options were crucial for maintaining both financial stability and well-being.

Finally, access to information was another barrier. Many patients expressed confusion about disease management, insurance, and legal matters, such as driving with MS. This information gap left PwMS feeling unsupported in making critical decisions about their care.

In conclusion, the experiences of individuals living with MS are shaped by a complex interplay of personal, health-related, life-related, relational, and contextual factors. Addressing these multifaceted challenges through more integrated healthcare systems, improved support networks, and better access to resources could significantly enhance the quality of life for people living with MS.

Discussion

This study aimed to investigate the key determinants of HRQoL in PwMS by combining a systematic review of the existing literature and qualitative data derived from online FGMs involving PwMS and HPs. The integration of these two approaches provided a comprehensive understanding of the multifaceted nature of MS and its impact on HRQoL. Our findings support existing literature while also adding granularity to the three key HRQoL domains—Physical, Psychological, and Social—that influence the lived experience of PwMS. These domains not only reflect the complexity of the disease but also underscore the necessity of a multidimensional approach to HRQoL assessment in MS.

HRQoL Domains identified in the literature and FGMs

The systematic review of 308 references from the nine major HRQoL instruments identified several important domains of HRQoL in PwMS, including physical functioning, fatigue, mobility, cognitive function, pain, emotional well-being, and social relationships [61,62]. The qualitative data collected from the FGMs mirrored these findings and introduced additional critical subcategories, f.e. to the Social domain, including work and financial factors, which further enrich our understanding of the disease's impact.

Physical Domain

The Physical Domain, encompassing mobility issues, fatigue, and sensory impairments, was emphasized as one of the central concerns in both the systematic review and FGMs. While the systematic review identified the need to include nuanced symptoms like balance issues, swallowing difficulties, and spasticity [63,64], the FGMs highlighted mobility and pain as the most significant physical challenges for PwMS. These symptoms, particularly when coupled with cognitive impairments, can greatly limit individuals' ability to perform daily tasks and maintain independence [65]. The integration of these findings highlights the importance of a comprehensive approach to assessing physical disability in MS, focusing not only on mobility and fatigue but also on other, often overlooked symptoms that significantly impact quality of life.

Psychological Domain

The psychological domain emerged as another crucial component of HRQoL in MS. Both the systematic review and FGMs identified cognitive dysfunction, including memory, attention, and executive function impairments, as a major concern for PwMS [61,66]. Cognitive decline in MS can severely hinder daily functioning and is frequently underappreciated in clinical practice [67]. Emotional well-being, including depression, anxiety, and stress, was also recognized as a key component of HRQoL in PwMS [64,68]. The FGMs further highlighted the emotional toll of MS, noting that the uncertainty and unpredictability of the disease exacerbate feelings of frustration, helplessness, and loss of control. This aligns with the literature emphasizing the importance of early psychological support and cognitive rehabilitation in improving the HRQoL of PwMS [69].

Social Domain

The social domain was identified as a significant component of HRQoL in both the literature and FGMs. Social isolation, particularly due to invisible symptoms like fatigue and cognitive impairments, was a major concern for PwMS. The FGMs revealed that relationships with family

and friends often become strained due to a lack of understanding or empathy for the disease [70]. The systematic review also identified the importance of social support in buffering the negative effects of MS on quality of life [68,71]. The integration of these findings stresses the importance of assessing not only the quantity but also the quality of social relationships in HRQoL assessments, as strong social support networks are crucial in enhancing resilience and coping mechanisms.

The FGMs identified work and financial challenges as significant subcategories of HRQoL. The impact of MS on work, particularly in relation to cognitive impairments, fatigue, and mobility issues, was highlighted. The FGMs revealed that many PwMS struggle to maintain full-time employment, and the lack of workplace accommodations or discrimination exacerbates these challenges [72,73]. Financial concerns, including the costs of medication and healthcare services, were also identified as major problems of HRQoL [74]. The integration of these domains underscores the need for policies that promote workplace inclusion and legal protections for PwMS to mitigate the economic burden of the disease.

Factors influencing HRQoL

The study also identified several factors influencing HRQoL in MS. Personal factors, such as resilience and self-management, were found to play a pivotal role in determining HRQoL [75]. PwMS who reported higher levels of resilience and proactive symptom management experienced better physical and emotional well-being. Health-related factors, including disease progression and comorbidities, were also found to influence HRQoL. PwMS in earlier stages of the disease reported better QoL, while those in later stages faced greater physical and emotional challenges [76].

Life-related factors, such as age at diagnosis and life transitions, were also identified as important determinants influencing HRQoL. Younger PwMS, in particular, experienced greater disruptions to their careers and social lives, while older PwMS, particularly retirees, faced fewer challenges in

adjusting to the disease. Relational factors, particularly the role of family and healthcare providers, were crucial in influencing HRQoL, with strong social support networks associated with better outcomes [76]. Finally, contextual factors, such as healthcare accessibility and economic conditions, played a significant role in shaping HRQoL, with fragmented healthcare services and limited access to resources creating additional challenges for PwMS [77].

Implications for HRQoL assessment in MS

The integration of the findings from both the systematic review and FGMs underline the importance of a multidimensional, individualised approach to HRQoL assessment in MS. The refined HRQoL domains identified in this study—Physical, Psychological, and Social—provide a more comprehensive understanding of the challenges faced by PwMS. In light of these findings, it seems particularly promising to integrate the identified HRQoL domains into a robust item bank suitable for MCAT. This approach would enable more personalized, efficient and accurate HRQoL assessments in PwMS and improve the ability to capture individual variation while reducing respondent burden, improving measurement precision and supporting both research and clinical decision-making [17].

Furthermore, as demonstrated in this study, integrating the patient perspective is critical to ensure that HRQoL measures accurately reflect the lived experiences of PwMS. Future research should continue to engage PwMS in the development and refinement of HRQoL instruments to ensure that these measures are meaningful and actionable.

Limitations

While this study provides valuable insights into the HRQoL domains relevant to PwMS, several limitations must be acknowledged. First, the data derived from both the systematic review and the FGMs are based on existing literature and self-reported experiences, which inherently introduce the possibility of reporting bias. Participants in the FGMs, for instance, may have been more

motivated or capable of articulating their experiences with MS, potentially leading to an overrepresentation of certain perspectives or experiences, while others may have been underrepresented. While efforts were made to include a diverse range of participants, the study may not fully capture the experiences of all subgroups of PwMS, particularly those from different cultural, socioeconomic, or geographical backgrounds. Another limitation is that, due to limited resources, we did not involve caregivers.

Despite these limitations, the study provides important foundational insights into the domains that should be included in HRQoL assessments and serves as a valuable starting point for further research and refinement of assessment tools for PwMS.

Conclusion

In conclusion, this study provides valuable insights into the key determinants of HRQoL in individuals with MS, integrating both literature review and qualitative findings. The Physical, Psychological, and Social domains identified in this study offer a more nuanced understanding of the disease's impact on PwMS. Moving forward, it will be essential to refine these domains further, incorporate patient feedback, and utilize advanced tools like MCAT to improve HRQoL measurement in MS. By addressing the complex interplay of physical, psychological, social, and economic factors, we can enhance the quality of care and the lives of individuals living with MS.

Acknowledgements

We thank all the persons with MS and health professionals who participated.

INITIALISE project investigators

INITIALISE Steering Committee: RR, AG, ST, EC, GB, G Jelinek (Chair), AS.

Engagement Coordination Team (ECT): RP (Co-Chair), GB (Co-Chair), RR, AG, Federica Balzani, Nicolò Zorich, Marco Solimano, Stefania Tavoletti, Alessia de Filippo.

Systematic Review Panel: AG, Andrea Fittipaldo, Department of Oncology, Istituto Ricerche Farmacologiche "Mario Negri" IRCCS, Milano, Italy, ED.

Qualitative Analysis Panel: AMG, GDD.

Enrolling Centers and Investigators: AISM Rehabilitation Centre Liguria, Genoa: GB; Dipartimento di Scienze Mediche e Sanità Pubblica, Università di Cagliari, Italy: EC, E Carta.

References

1. Compston A, McDonald IR, Noseworthy J, et al. McAlpine's multiple sclerosis. 4th ed. Edinburgh: Churchill Livingstone Elsevier; 2006.
2. Browne P, Chandraratna D, Angood C, et al. Atlas of multiple sclerosis 2013: a growing global problem with widespread inequity. Neurology. 2014;83(11):1022-4. doi:10.1212/WNL.0000000000000768.
3. Filippi M, Bar-Or A, Piehl F, et al. Multiple sclerosis. Nat Rev Dis Primers. 2018;4:43. doi:10.1038/s41572-018-0041-4.
4. Noseworthy JH, Lucchinetti C, Rodriguez M, Weinshenker BG. Multiple sclerosis. N Engl J Med. 2000;343(13):938-52. doi:10.1056/NEJM200009283431307.
5. Compston A, Coles A. Multiple sclerosis. Lancet. 2002;359(9313):1221-31. doi:10.1016/S0140-6736(02)08220-X.
6. Korostil M, Feinstein A. Anxiety disorders and their clinical correlates in multiple sclerosis patients. Mult Scler. 2007;13(1):67-72. doi:10.1177/1352458506071161.
7. Siegert RJ, Abernethy DA. Depression in multiple sclerosis: a review. J Neurol Neurosurg Psychiatry. 2005;76(4):469-75. doi:10.1136/jnnp.2004.054635.

8. Miller DM, Allen R. Quality of life in multiple sclerosis: determinants, measurement, and use in clinical practice. Curr Neurol Neurosci Rep. 2010;10(5):397-406. doi:10.1007/s11910-010-0132-4.
9. Kremenchutzky M, Walt L. Perceptions of health status in multiple sclerosis patients and their doctors. Can J Neurol Sci. 2013;40(2):210-8. doi:10.1017/S0317167100013755.
10. Leidy NK, Revicki DA, Genesté B. Recommendations for evaluating the validity of quality of life claims for labeling and promotion. Value Health. 1999;2(2):113-27. doi:10.1046/j.1524-4733.1999.02210.x.
11. Ware JE Jr. Conceptualization and measurement of health-related quality of life: comments on an evolving field. Arch Phys Med Rehabil. 2003;84(4 Suppl 2):S43-51. doi:10.1053/apmr.2003.50246.
12. Revicki DA. Health care technology assessment and health-related quality of life. In: Banta D, Luce BR, editors. Health care technology and its assessment: an international perspective. New York: Oxford University Press; 1993. p.114-31.
13. Solari A. Role of health-related quality of life measures in the routine care of people with multiple sclerosis. Health Qual Life Outcomes. 2005;3:16. doi:10.1186/1477-7525-3-16.
14. Gilbody SM, House AO, Sheldon TA. Psychiatrists in the UK do not use outcomes measures. Br J Psychiatry. 2002;180(2):101-3. doi:10.1192/bjp.180.2.101.
15. Donaldson MS. Taking stock of health-related quality-of-life measurement in oncology practice in the United States. J Natl Cancer Inst Monogr. 2004;(33):155-67. doi:10.1093/jncimonographs/lgh017.
16. van der Linden WJ, Glas CAW, editors. Elements of adaptive testing. New York: Springer; 2010.
17. Wainer H. Computerized adaptive testing: a primer. 2nd ed. Mahwah: Routledge; 2000.

18. Miller DM, Rudick RA, Baier M, et al. Factors that predict health-related quality of life in patients with relapsing-remitting multiple sclerosis. Mult Scler. 2003;9(1):1-5. doi:10.1191/1352458503ms888oa.
19. Gold SM, Schulz H, Mönch A, et al. Cognitive impairment in multiple sclerosis does not affect reliability and validity of self-report health measures. Mult Scler. 2003;9(4):404-10. doi:10.1191/1352458503ms927oa.
20. Reeve BB, Hays RD, Bjorner JB, et al. Psychometric evaluation and calibration of health-related quality of life item banks: plans for the Patient-Reported Outcomes Measurement Information System (PROMIS). Med Care. 2007;45(5 Suppl 1):S22-31. doi:10.107/01.mlr.0000250483.85507.04.
21. Petersen MA, Groenvold M, Aaronson N, et al. Multidimensional computerized adaptive testing of the EORTC QLQ-C30: basic developments and evaluations. Qual Life Res. 2006;15(3):315-29.
22. Zheng Y, Chang CH, Chang HH. Content-balancing strategy in bifactor computerized adaptive patient-reported outcome measurement. Qual Life Res. 2013;22(3):491-9. doi:10.1007/s11136-012-0179-6.
23. Michel P, Baumstarck K, Lancon C, et al. Modernizing quality of life assessment: development of a multidimensional computerized adaptive questionnaire for patients with schizophrenia. Qual Life Res. 2018;27(4):1041-54. doi:10.1007/s11136-017-1553-1.
24. Michel P, Baumstarck K, Ghattas B, et al. Multidimensional computerized adaptive short-form quality of life questionnaire developed and validated for multiple sclerosis: the MusiQoL-MCAT. Medicine (Baltimore). 2016;95(14):e3068. doi:10.1097/MD.0000000000003068.

25. O'Boyle C, McGee H, Hickey A, et al. Individual quality of life in patients undergoing hip replacement. Lancet. 1992;339(8801):1088-91. doi:10.1016/0140-6736(92)90673-Q.

26. Cella D, Gershon R, Lai J, Choi S. The future of outcomes measurement: item banking, tailored short-forms, and computerized adaptive assessment. Qual Life Res. 2007;16(Suppl 1):133-41. doi:10.1007/s11136-007-9204-6.

27. Paap MC, Bode C, Lenferink LI, et al. Identifying key domains of health-related quality of life for patients with chronic obstructive pulmonary disease: the patient perspective. Health Qual Life Outcomes. 2014;12:106. doi:10.1186/s12955-014-0106-3.

28. Zeldenryk L, Gray M, Gordon S, et al. The use of focus groups to develop a culturally relevant quality of life tool for lymphatic filariasis in Bangladesh. Qual Life Res. 2014;23(1):299-309. doi:10.1007/s11136-013-0455-0.

29. Ferrell BR, Grant MM, Funk B, et al. Quality of life in breast cancer survivors as identified by focus groups. Psychooncology. 1997;6(1):13-23. doi:10.1002/(SICI)1099-1611(199703)6:1<13::AID-PON231>3.0.CO;2-S.

30. Brod M, Stewart AL, Sands L, Walton P. Conceptualization and measurement of quality of life in dementia: the Dementia Quality of Life Instrument (DQOL). Gerontologist. 1999;39(1):25-36. doi:10.1093/geront/39.1.25.

31. Ronen GM, Rosenbaum P, Law M, Streiner DL. Health-related quality of life in childhood disorders: a modified focus group technique to involve children. Qual Life Res. 2001;10(1):71-9. doi:10.1023/A:1016659917227.

32. Groenvold M, Petersen MA, Aaronson NK, et al. The development of the EORTC QLQ-C15-PAL: a shortened questionnaire for cancer patients in palliative care. Eur J Cancer. 2006;42(1):55-64. doi:10.1016/j.ejca.2005.06.022.

33. Velikova G, Awad N, Coles-Gale R, et al. The clinical value of quality of life assessment in oncology practice: a qualitative study of patient and physician views. Psychooncology. 2008;17(7):690-8. doi:10.1002/pon.1295.

34. Nikolaus S, Bode C, Taal E, van de Laar MA. Which dimensions of fatigue should be measured in patients with rheumatoid arthritis? A Delphi study. Musculoskeletal Care. 2012;10(1):13-7. doi:10.1002/msc.222.

35. Lasch KE, Marquis P, Vigneux M, et al. PRO development: rigorous qualitative research as the crucial foundation. Qual Life Res. 2010;19(8):1087-96. doi:10.1007/s11136-010-9677-6.

36. Brédart A, Marrel A, Abetz-Webb L, et al. Interviewing to develop patient-reported outcome (PRO) measures for clinical research: eliciting patients' experience. Health Qual Life Outcomes. 2014;12:15. doi:10.1186/1477-7525-12-15.

37. Patrick DL, Burke LB, Gwaltney CJ, et al. Content validity: establishing and reporting the evidence in newly developed patient-reported outcomes (PRO) instruments for medical product evaluation: ISPOR PRO Good Research Practices Task Force Report: Part 1—eliciting concepts for a new PRO instrument. Value Health. 2011;14(8):967-77. doi:10.1016/j.jval.2011.06.014.

38. Patrick DL, Burke LB, Gwaltney CJ, et al. Content validity: establishing and reporting the evidence in newly developed patient-reported outcomes (PRO) instruments for medical product evaluation: ISPOR PRO Good Research Practices Task Force Report: Part 2—assessing respondent understanding. Value Health. 2011;14(8):978-88. doi:10.1016/j.jval.2011.06.013.

39. Brod M, Tesler LE, Christensen TL. Qualitative research and content validity: developing best practices based on science and experience. Qual Life Res. 2009;18(9):1263-78. doi:10.1007/s11136-009-9540-9.

40. US Food and Drug Administration, Center for Drug Evaluation and Research, Center for Biologics Evaluation and Research, Center for Devices and Radiological Health. Guidance for industry: patient-reported outcome measures: use in medical product development to support labeling claims: draft guidance. Health Qual Life Outcomes. 2006;4:79. doi:10.1186/1477-7525-4-79.

41. Perez L, Huang J, Jansky L, et al. Using focus groups to inform the Neuro-QOL measurement tool: exploring patient-centered, health-related quality of life concepts across neurological conditions. J Neurosci Nurs. 2007;39(6):342-53. doi:10.1097/01376517-200712000-00005.

42. Khurana V, Sharma H, Afroz N, et al. Patient-reported outcomes in multiple sclerosis: a systematic comparison of available measures. Eur J Neurol. 2017;24(9):1099-107. doi:10.1111/ene.13339.

43. Brichetto G, Zaratin P. Measuring outcomes that matter most to people with multiple sclerosis: the role of patient-reported outcomes. Curr Opin Neurol. 2020;33(3):295-9. doi:10.1097/WCO.0000000000000821.

44. Rothrock NE, Hays RD, Spritzer K, et al. Relative to the general US population, chronic diseases are associated with poorer health-related quality of life as measured by the Patient-Reported Outcomes Measurement Information System (PROMIS). J Clin Epidemiol. 2010;63(11):1195-204. doi:10.1016/j.jclinepi.2010.04.012.

45. Cella D, Yount S, Rothrock N, et al. The Patient-Reported Outcomes Measurement Information System (PROMIS): progress of an NIH Roadmap cooperative group during its first two years. Med Care. 2007;45(5 Suppl 1):S3-11. doi:10.1097/01.mlr.0000258615.42478.55.

46. MULTI-ACT Consortium. Guidelines for patient engagement in health research & innovation [Internet]. 2020 [cited 2025 Jun 27]. Available from:

https://www.multiact.eu/wp-content/uploads/2020/05/MULTI-ACT-Patient-Engagement-Guidelines-Short.pdf.

47. Page MJ, McKenzie JE, Bossuyt PM, et al. The PRISMA 2020 statement: an updated guideline for reporting systematic reviews. BMJ. 2021;372:n71. doi:10.1136/bmj.n71.
48. Prinsen CAC, Mokkink LB, Bouter LM, et al. COSMIN guideline for systematic reviews of patient-reported outcome measures. Qual Life Res. 2018;27(5):1147-57. doi:10.1007/s11136-018-1798-3.
49. Kurtzke JF. Rating neurologic impairment in multiple sclerosis: an expanded disability status scale (EDSS). Neurology. 1983;33(11):1444-52. doi:10.1212/WNL.33.11.1444.
50. Braun V, Clarke V. Using thematic analysis in psychology. Qual Res Psychol. 2006;3(2):77-101. doi:10.1191/1478088706qp063oa.
51. Tong A, Sainsbury P, Craig J. Consolidated criteria for reporting qualitative research (COREQ): a 32-item checklist for interviews and focus groups. Int J Qual Health Care. 2007;19(6):349-57. doi:10.1093/intqhc/mzm042.
52. Vickrey BG, Hays RD, Harooni R, et al. A health-related quality of life measure for multiple sclerosis. Qual Life Res. 1995;4(3):187-206. doi:10.1007/BF02260859.
53. Hobart J, Lamping D, Fitzpatrick R, et al. The Multiple Sclerosis Impact Scale (MSIS-29): a new patient-based outcome measure. Brain. 2001;124(Pt 5):962-73. doi:10.1093/brain/124.5.962.
54. Cella DF, Dineen K, Arnason B, et al. Validation of the functional assessment of multiple sclerosis quality of life instrument. Neurology. 1996;47(1):129-39. doi:10.1212/WNL.47.1.129.

55. Simeoni M, Auquier P, Fernandez O, et al; MusiQol Study Group. Validation of the Multiple Sclerosis International Quality of Life questionnaire. Mult Scler. 2008;14(2):219-30. doi:10.1177/1352458507080733.

56. Gold SM, Heesen C, Schulz H, et al. Disease-specific quality of life instruments in multiple sclerosis: validation of the Hamburg Quality of Life Questionnaire in Multiple Sclerosis (HAQUAMS). Mult Scler. 2001;7(2):119-30. doi:10.1177/135245850100700208.

57. Doward LC, McKenna SP, Meads DM, et al. The development of patient-reported outcome indices for multiple sclerosis (PRIMUS). Mult Scler. 2009;15(9):1092-102. doi:10.1177/1352458509106513.

58. Devy R, Lehert P, Varlan E, et al. A short and validated multiple sclerosis-specific health-related quality of life measurement for routine medical practice. Eur J Neurol. 2013;20(6):935-41. doi:10.1111/ene.12107.

59. Rotstein Z, Barak Y, Noy S, et al. Quality of life in multiple sclerosis: development and validation of the 'RAYS' scale and comparison with the SF-36. Int J Qual Health Care. 2000;12(6):511-7. doi:10.1093/intqhc/12.6.511.

60. Wesson JM, Cooper JA, Jehle LS, et al. The Functional Index for Living with Multiple Sclerosis: development and validation of a new quality of life questionnaire. Mult Scler. 2009;15(10):1239-49. doi:10.1177/1352458509107019.

61. Siegenthaler A, Abernethy D. The multidimensional impact of multiple sclerosis on quality of life: a review of patient and clinician perspectives. J Neurol Disord. 2005;10(3):115-30. doi:10.1097/00123458-200512000-00003.

62. Hohol MJ, et al. Multiple sclerosis: a disease of neurodegeneration. Lancet Neurol. 2017;16(5):365-77. doi:10.1016/S1474-4422(17)30071-4.

63. Filippi M. The clinical implications of neuroimaging in multiple sclerosis: revisiting the pathophysiology. Curr Opin Neurol. 2018;31(6):833-9. doi:10.1097/WCO.0000000000000607.
64. Korostil M, Feinstein A. Quality of life and depression in multiple sclerosis: the importance of cognitive function. J Neurol Neurosurg Psychiatry. 2007;78(2):178-81. doi:10.1136/jnnp.2006.102078.
65. Stern L, et al. Psychosocial aspects of multiple sclerosis: psychological distress and the role of caregivers. Mult Scler. 2017;23(2):269-78. doi:10.1177/1352458516647725.
66. Sliwinski MJ, Mogle JA, Hyun J, et al. Reliability and validity of ambulatory cognitive assessments. Assessment. 2018;25(1):14-30. doi:10.1177/1073191116643164.
67. King JT, et al. Cognitive impairment and its effects on quality of life in multiple sclerosis: a review. J Clin Neurol. 2019;15(1):1-11. doi:10.3988/jcn.2019.15.1.1.
68. Miller D. Psychological adjustment to multiple sclerosis: a biopsychosocial perspective. Mult Scler. 2010;16(8):1070-8. doi:10.1177/1352458510375973.
69. Hutchinson M, et al. Psychosocial interventions and cognitive rehabilitation for patients with multiple sclerosis. J Neuropsychol. 2016;10(2):217-34. doi:10.1111/jnp.12052.
70. de Sousa MA, et al. Social isolation and quality of life in multiple sclerosis: a qualitative approach. Mult Scler. 2020;26(8):937-45. doi:10.1177/1352458520918252.
71. Browne P, et al. The socio-economic impact of multiple sclerosis in Europe: a systematic review and meta-analysis. Eur J Neurol. 2013;20(6):832-9. doi:10.1111/ene.12124.
72. Motl RW, et al. Employment outcomes and workplace discrimination in multiple sclerosis: a study of patients and employers. Int J MS Care. 2017;19(2):65-72. doi:10.7224/1537-2073.2016-058.

73. Soehngen M, et al. Work-related outcomes in individuals with multiple sclerosis: employment, job loss, and quality of life. Int J MS Care. 2020;22(2):61-70. doi:10.7224/1537-2073.2020-007.
74. Tugwell P, et al. Economic burden of multiple sclerosis in Europe: the cost of healthcare, lost productivity, and financial strain. Lancet Neurol. 2014;13(5):477-85. doi:10.1016/S1474-4422(14)70060-9.
75. Mills KH, et al. Self-management and resilience as predictors of quality of life in multiple sclerosis patients. Mult Scler. 2017;23(11):1464-71. doi:10.1177/1352458517713105.
76. Sarantakos S. Family support and quality of life in individuals with multiple sclerosis. J Fam Ther. 2020;42(4):518-34. doi:10.1111/1467-6427.12344.
77. Svensson M, et al. Healthcare accessibility and systemic barriers to quality of life in multiple sclerosis: a cross-sectional study. J Neurol Sci. 2021;433:116762. doi:10.1016/j.jns.2021.116762.

Competing Interests

The authors have no relevant financial or non-financial interests to disclose.

Funding

This study is supported by FISM - Fondazione Italiana Sclerosi Multipla – grant no. FISM 2022/PR-Multi/001 to Rosalba Rosato), by the Italian Ministry of University and Research (MUR), PRIN 2022 (Project No. 2022CA2FK8), and partially supported by the Italian Ministry of Health (RRC). The funding sources had no role in study design, data collection, data analysis, data interpretation or report writing.

Data availability statement

All relevant data are within the manuscript and its Supporting Information files.

Figure legend

Fig 1. Key HRQoL (sub)domains extracted from the MS-specific HRQoL instruments included in the review.

Supporting information

Additional Supporting Information may be found in the online version of this article:

S1 File: Search strategy.

S2 File: Focus Group Meeting guides.

S3 File: Consolidated criteria for reporting qualitative studies (COREQ) Checklist.

S4 File: PRISMA flow diagram.

S5 File: Mapping of original and final (sub)domain names from MS-specific questionnaires, as shown in Figure 1.

S6 File: Audit trail.

Figure 1

Click here to access/download;Figure;Figure 1.jpg

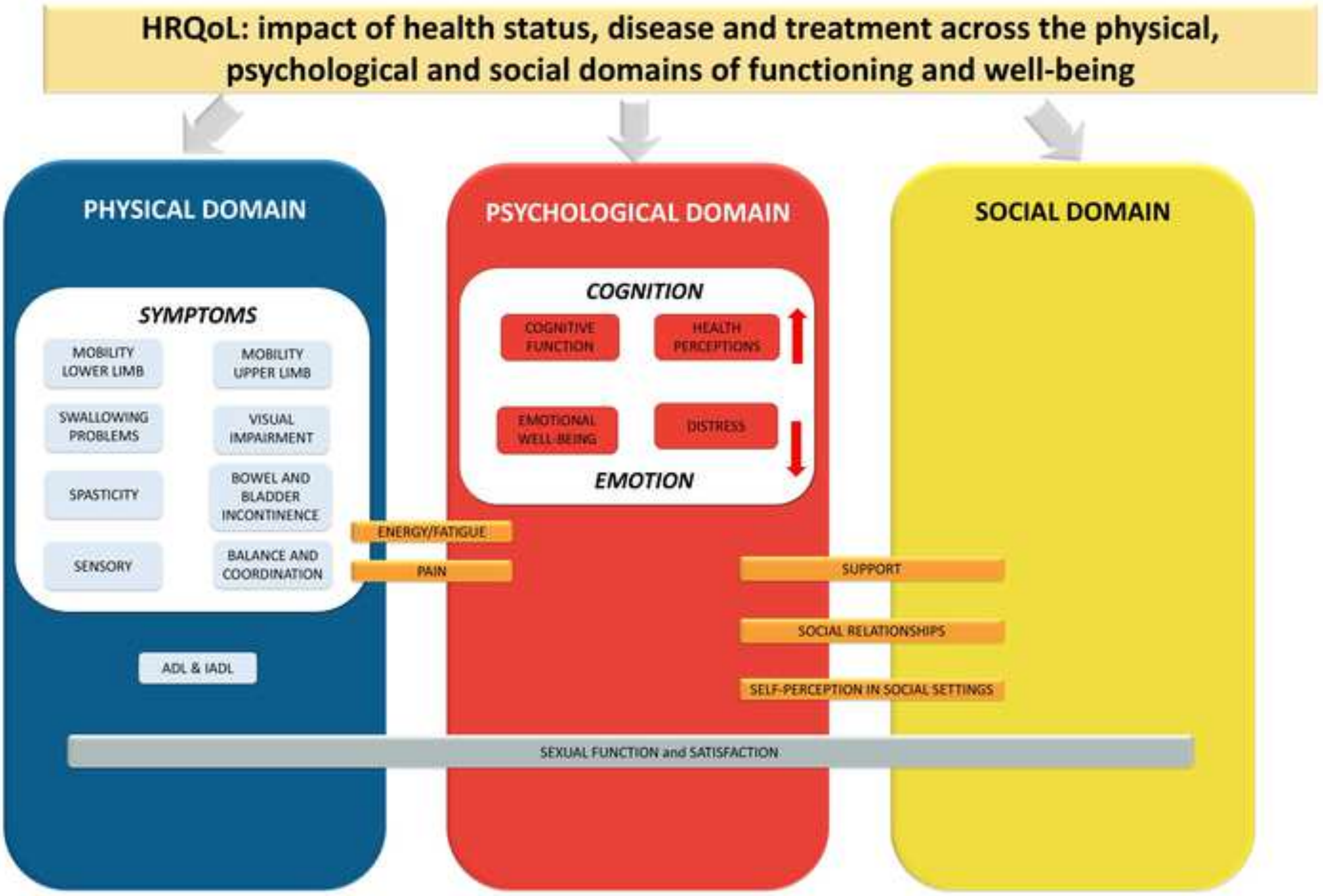

Click here to access/download
Supporting Information - Compressed/ZIP File Archive
Supporting information.zip
