# Supplementary material for: Quality of life domains revised by people with multiple sclerosis and healthcare professionals for adaptive measure development
Source: PLoS One. 2026 Jun 11;21(6):e0349034. doi: 10.1371/journal.pone.0349034 (PMC13257964; doi:10.1371/journal.pone.0349034)
Supplement: Attachment — Submitted filename: Response to Reviewers.docx [file pone.0349034.s008.docx]

**Response to Reviewers**

We sincerely thank the Reviewers for their valuable comments, which have greatly helped us to improve the quality and clarity of the manuscript.

***Reviewer #1***

*Thank you for inviting me to review your manuscript:
Great project, very precise implementation of research plan*
We thank very much this Reviewer for their appreciation of our project.

*I have some questions and comments for your manuscript*

1. *The WHOQol-BREF measures quality of life (QoL) on 4 domains – Physical, psychological, social, environmental QoL. Was this questionnaire never used to measure QoL in MS?*

We thank the reviewer for this comment. Indeed, this questionnaire was used to measure QoL in MS [e.g., Pomeroy et al. (2020). The WHOQOL‑BREF: a modern psychometric evaluation of its internal construct validity in people with multiple sclerosis. Quality of Life Research, 29, 1961–1972. https://doi.org/10.1007/s11136-020-02463-z]. However, as stated in the Methods, our scope was to ‘collect all MS-specific instruments that have been used to measure HRQoL in adults with MS’. So, we did not consider the WHOQOL-BREF as it is a generic HRQOL measure.

2. *In my understanding, you tried to find quantitative instruments used in research that measure QoL in MS.
   I could not find what these instruments did not include and what they should have measured in addition.*

We thank the Reviewer for their comment. We addressed this point by expanding the final paragraph of the Introduction (please, see also our response to point 7 of Reviewer 3), where we now provide a clearer overview of the HRQoL domains already described in MS-specific instruments, as follows: ‘Existing MS-specific HRQoL instruments—such as the MSQoL-54, MSIS-29, MusiQoL, and FAMS—typically assess domains including physical functioning, fatigue, pain, cognition, emotional well-being, and social relationships. However, these instruments differ in scope, structure, and conceptual coverage, and may not fully align with the domains that PwMS describe as most meaningful, such as self-perception, social participation, work-related challenges, and contextual or relational influences on daily life [46]. These underrepresented areas suggest that the current conceptualization of HRQoL in MS may not fully reflect patients’ lived experiences.

By integrating evidence from the literature with qualitative insights from PwMS and health professionals (HPs), the present study aims to refine and expand the conceptual framework of HRQoL in MS, thereby enhancing its content validity and ensuring stronger patient relevance for future adaptive measurement development’.

3. *You state that new instruments should be developed. I wonder if complementing already existing ones would be possible.*
   We thank the Reviewer for this comment. As stated in the Introduction, our aim was to work on the structure of HRQoL by identifying and selecting the most relevant HRQoL domains in MS. Our intention is not to disregard existing instruments, but to build upon them, integrating the domains they already cover with additional areas that emerged as underrepresented in our analyses and stakeholder discussions (e.g., work participation).

When we refer to the need for new instruments, we are not implying that measurement development is part of the present work. Rather, this study provides the conceptual groundwork for future initiatives that will aim to complement and extend existing MS-specific measures. Such future developments will be based on the framework established here, ensuring that subsequent tools are both conceptually comprehensive and aligned with patients’ lived experiences.

4. *Was it possible for all potential participants to meet virtually? Did all have access to the internet? If not, was there a plan how they could have been assisted?
   (e.g. drive home to them and offer proper assistance?)*

I confirm that all participants met virtually and that they all have access to the Internet.

5. *Did participants sign a consent form for being audiotaped?*
   Yes, they did.
6. *Please structure your paper that it is easier to read and to follow. It is a very long manuscript. Please try to make it shorter, at the same time clearer.*

We thank the Reviewer for their helpful suggestion. Following their recommendation, we carefully revised the manuscript to improve its readability, coherence, and overall structure. We streamlined the Introduction, Methods and Results sections by removing redundant or overly detailed descriptions and improved transitions between the systematic review and qualitative phases. In addition, we incorporated a more structured comparison between the domains assessed by existing questionnaires and the categories that emerged from the qualitative analysis, in order to better address the aims of our study and respond to the reviewers’ comments. We also judged the “Factors Influencing HRQoL” subsection to be less relevant to the specific objectives of the manuscript, and therefore removed it, which allowed us to shorten the text (5993 words instead of 6905) and, we hope, improve its clarity. In line with this overall effort, we also substantially revised the Discussion to enhance its focus, coherence, and alignment with the study’s revised structure.

7. *Table 1: I would have expected more domains.*
   Thank you for this observation. Table 1 intentionally reports only the main categories of the HRQoL domains. All categories are then expanded and articulated into detailed subcategories in Tables 2–4, and further illustrated in the audit trail (S6 File). Our aim was to keep Table 1 concise and readable, while providing full domain granularity in the subsequent tables and supplementary materials.
8. *Please add list of abbreviations.*

We have now added the list of abbreviations.

9. *Persons with MS were included and health professionals. Support persons were not included. They can provide great information regarding the QoL of PwMS. They should have been included in the focus groups.*
   We agree with the Reviewer’s comment. As acknowledged in the Limitations section, we did not involve caregivers due to limited resources. We fully recognize, however, that support persons can provide valuable complementary perspectives on the QoL of PwMS, particularly regarding aspects of daily functioning and relational dynamics that may be less visible to patients themselves. We have now further clarified this point in the manuscript and emphasized that future research should incorporate caregivers’ perspectives to strengthen the comprehensiveness and applicability of the conceptual framework, as follows: ‘Moreover, caregivers were not included, limiting insight into indirect or externally observed aspects of HRQoL’.
10. *If you want to submit this as a paper, be aware that it should be much shorter.
    You can put information, which is maybe too detailed, to the Appendix. Not everything has to be on the main paper.*
    Please, see the reply to point 6 above.
11. *You identified physical, psychological and social domains as important.
    We already know this from former qualitative and quantitative research.*
    We thank the Reviewer for this important and thoughtful comment, which prompted us to reflect deeply on the clarity and contribution of our work and ultimately led to substantial improvements throughout the manuscript. We agree that the Physical, Psychological, and Social domains are well-established in the MS HRQoL literature, and we do not present them as novel findings. As clarified in our response to Comment 19, the contribution of our study lies instead in the refinement, specification, and extension of these domains. Through the integration of qualitative data, we identified additional subdomains and nuances—such as work-related challenges, financial concerns, contextual influences, and aspects of self-perception—that are not consistently or comprehensively captured in existing MS-specific instruments.

To better highlight this contribution, we substantially revised the manuscript. As noted in our response to point 6 above, we restructured several sections to improve clarity and readability, streamlined redundant content, and strengthened transitions between the systematic review and qualitative components. Importantly, to more clearly convey what is new in our study and why it matters, we introduced a dedicated methodological section (“Cross-Mapping of HRQoL Instruments and Qualitative Findings”). This section systematically compares qualitative categories and subcategories with the items of MS-specific HRQoL instruments, thereby identifying which aspects of the lived experience of PwMS are sufficiently represented and which remain underrepresented or absent.

We incorporated the outcomes of this cross-mapping into the Results section and revised the Discussion accordingly. These changes more clearly articulate the novelty and relevance of our work: not the identification of new overarching domains, but the addition of conceptual granularity and the systematic identification of measurement gaps that can inform the refinement of existing HRQoL instruments or the development of new patient-centered tools.

We are grateful to the Reviewer for raising this point, which significantly strengthened the focus and contribution of the manuscript.

12. *You state ‘The growing interest in CAT instruments in HRQoL research has led to the development of a range of CAT instruments by the Patient-Reported Outcomes Measurement Information System (PROMIS) supported by the National Institute of Health [20], which currently offers 3 to 10 items for domains such as fatigue, physical functioning, pain, depression, anxiety, and social functioning (www.nihpromis.org). For the assessment of multidimensional constructs with correlated dimensions, the Multidimensional Computer Adaptive Testing (MCAT) approach is more appropriate, but, as far as we know, there is few evidence of HRQoL instruments using such a methodology to fixed-length HRQOL questionnaires, including the MS domain [21-24]. To address this gap, we took a comprehensive approach based on a literature review and informed by the patient voice to identify relevant HRQoL dimensions that ensure both content validity and clinical utility of the assessment [20,25,26].’
    My question: did you use MCAT by facilitating the focus groups online?*

No, we did not use MCAT to facilitate the online focus groups. As already stated in the manuscript, the aim of our work was to define a well-structured domain framework of HRQoL in multiple sclerosis. Such a well-defined domain structure may be useful for several future research directions, including the development of psychometrically sound item banks that would allow for accurate, efficient, and individualized assessment, potentially through CAT or MCAT approaches. Therefore, MCAT was not applied in the present study but is envisioned as a possible methodological development building on our findings.

13. *I don’t understand why identifying quantitative questionnaires used was necessary first before the qualitative focus group discussions.
    Please clarify.*
    We thank the Reviewer for their comment. The systematic identification of MS-specific HRQoL questionnaires was conducted first to provide an evidence-based foundation for the qualitative phase. The purpose was to extract HRQoL domains already represented in the literature and use them to develop the focus group guides.

As described in the Methods, ‘[…] the deductive process, informed by findings from the literature review (existing HRQoL domains), applied category labels consistent with the literature when appropriate.’ This sequential design ensured that focus group discussions with PwMS and clinical professionals were grounded in the existing conceptual framework, while still allowing new or refined domains to emerge inductively from participants’ perspectives.

14. *You state that your goal was:
    ‘ 1. to conduct a systematic literature review to identify the most relevant HRQoL domains in MS; 2) to discuss the identified HRQoL domains with PwMS and eventually supplement them with additional relevant domains.’
    What additional categories would you add to understanding HRQOL?*
    Please, see our replies to point 6 and 11 above, and point 19 of Reviewer 3.
15. *You state ‘To identify additional studies, we also reviewed published systematic reviews and the reference lists of the selected articles’. Good job!*

We thank very much this Reviewer for their appreciation.

16. *You picked PwMS who should be in the FG interviews? Is this correct? Would it have been possible you would have allowed random participants?
    Please specify ‘Investigators at each participating centre identified potentially eligible PwMS from their database and invited them to participate in the study’.*
    We thank the Reviewer for this comment. As stated in the manuscript, FGM participants were selected through purposive sampling, which is an established approach in qualitative research. This method was chosen to ensure a broad range of perspectives and experiences in relation to HRQoL, including variation in age, disease duration, disability level, and psychosocial background. A random selection would not have been appropriate for the qualitative design of this study, whose aim was conceptual exploration and diversity of viewpoints, rather than statistical representativeness.
17. *Regarding Inclusion/Exclusion Criteria:
    Try to say in Methods/Study Design/Study Participants who were included and what the inclusion criteria were.*

The eligibility criteria for PwMS and HPs are reported in the FGM enrolment procedure.

18. *FGM Conduct: that paragraph could be said in 2 sentences. Try to reduce text.*
    Following this Reviewer’s suggestion, we have now shortened the paragraph, as follows: ‘Each focus group meeting (FGM) included 6–10 participants and two facilitators (AMG and GDD) and was conducted online via Zoom to enhance accessibility and feasibility for PwMS. Sessions followed a semi-structured guide covering pre-specified topics, were audio-recorded, fully transcribed, and subsequently reviewed by participants for validation’.
19. *Try to create one chapter called Methods and Study Design and describe all Methods used in this study.
    Describe under Analysis all Methods you used.
    You have Methods for the focus groups and methods for identifying literature on the search engines.*

Following this Reviewer’s suggestion, we have now revised the structure of the Methods section to present all methodological components under a single heading entitled “Methods and Study Design.”

20. *Under results you already describe what you identified with your methods used. That’s good.*

We thank very much the Reviewer for their appreciation.

*For the qualitative study don’t mix participants and setting with the results. Under results present all results, under Methods present all Methods used (thus for your focus groups as well).*
We thank the Reviewer for this important comment regarding the separation of Methods and Results. We fully agree that the procedural aspects of a qualitative study—such as recruitment strategy, inclusion/exclusion criteria, setting, and data collection methods—belong to the Methods section.

However, we would like to clarify that the outcome of participant recruitment—namely the number of participants actually enrolled, characteristics of the final sample, and any refusals or drop-outs—represents a result of the recruitment process, not a methodological detail known a priori. Reporting these outcomes in the Results section is consistent with standard guidance for qualitative research reporting. For example, O’Brien et al. (2014), in the widely cited Standards for Reporting Qualitative Research (SRQR), state: “In the Results section, describe the participants of the study. […] Where the Sampling Strategy section in the Methods section described the aims and strategies, this section describes the results of those efforts.” (O’Brien BC, Harris IB, Beckman TJ, Reed DA, Cook DA. Standards for Reporting Qualitative Research: A Synthesis of Recommendations. Acad Med. 2014;89(9):1245‑1251. doi:10.1097/ACM.0000000000000388).

Similarly, reporting the actual composition of the sample is emphasized by the COREQ framework: “The Results section should contain a description of the sociodemographic and other relevant characteristics of any human subjects in the sample.”
(Tong A, Sainsbury P, Craig J. Consolidated criteria for reporting qualitative research (COREQ): a 32-item checklist for interviews and focus groups. Int J Qual Health Care. 2007;19(6):349‑357. doi:10.1093/intqhc/mzm042)

Finally, Dossett et al. (2021) note that transparent reporting of who ultimately participated enhances rigor and reproducibility: “Participant characteristics and numbers should be reported as results of the recruitment and sampling methods, not as a priori methodological information.” (Dossett LA, Kaji AH, Cochran A. Reporting Guidelines for Qualitative Studies in Surgery. JAMA Surg. 2021;156(7):654‑660. doi:10.1001/jamasurg.2021.1271).

Based on these principles, we have maintained all procedural aspects (how we recruited participants, inclusion criteria, setting, data collection) in the Methods section, while reporting the outcome of recruitment (number enrolled, refusals/drop-outs, final sample characteristics) in a dedicated subsection at the start of the Results. This organization ensures both methodological clarity and transparency, in line with widely recognized standards for qualitative research.

We hope these clarifications address the Reviewer’s concern while adhering to established qualitative research reporting guidelines.

21. *Why is cognitive function under psychological and not under physical?
    Is this because it was discussed in the focus group interviews? Please specify.*
    We thank the Reviewer for their comment. The decision to classify cognitive function under the Psychological rather than the Physical domain was both conceptually and empirically grounded. As shown in the mapping of MS-specific HRQoL instruments (Supplementary File 5), cognitive function is an original domain in the MSQoL-54 and FILMS and is conceptually integrated within the mental/psychological structure in these instruments. Similarly, in the MusiQoL, psychological well-being encompasses cognitive function together with emotional well-being and health perceptions. This categorization is consistent with the established HRQoL frameworks, where cognitive processes, such as attention, memory, and executive function, are considered aspects of psychological rather than physical functioning. Focus group participants also described cognitive difficulties mainly in connection with emotional balance, self-perception, and coping, reinforcing this conceptual placement.
22. *Why was it necessary to identify all different quantitative questionnaires first before you started the focus group discussions?
    The qualitative results are totally independent.
    Is this correct? Would it have made more sense to conduct the focus group first and do the questionnaires then? Please specify.*
    Please, see point 13 above.
23. *Your focus group interviews are great. No novelty was identified. If so, please specify and explain the advantages of mixing PwMS and Clinical Professionals in the focus group interviews.*

We thank the Reviewer for this comment. While no entirely novel HRQoL domains emerged, the key advantage of mixing people with MS and health professionals within the focus groups lies in the integration of complementary perspectives. PwMS contributed first-hand, lived experience of HRQoL, whereas professionals brought an external, longitudinal, and comparative viewpoint, derived from caring for many individuals with MS across different disease stages and clinical profiles. This combination allowed us to contrast, validate, and contextualize patient-reported experiences within a broader clinical framework, thereby strengthening the interpretability and relevance of the identified domains.

We also acknowledge that caregivers were not included, which may limit insight into indirectly observed or externally mediated aspects of HRQoL. This represents a limitation of the present study and an important direction for future research.

24. *No changes for questionnaires were discussed based on the results of your focus groups.
    You say ‘the necessity of a multidimensional approach to HRQoL assessment in MS.’ But you don’t make suggestions how this should look like. You say ‘it seems particularly promising to integrate the identified HRQoL domains into a robust item bank suitable for MCAT. This approach would enable more personalized, efficient and accurate HRQoL assessments in PwMS and improve the ability to capture individual variation while reducing respondent burden, improving measurement precision and supporting both research and clinical decision-making’.
    But how can that be done in daily clinical settings? Who would cover the costs? The extra time needed? Please try to discuss in the Discussion chapter.*

We thank the Reviewer for this thoughtful comment. We agree that clarifying how a multidimensional HRQoL framework—and, in the future, an adaptive item-bank–based system—could be implemented in routine clinical practice is essential. Our study did not aim to modify existing questionnaires or to introduce a new instrument, but rather to provide the conceptual foundation needed for subsequent measurement development. However, we acknowledge the importance of discussing feasibility aspects, and we have now expanded the Discussion accordingly, as follows: ‘A practical route for implementing this framework in clinical settings involves digital, modular ePRO (electronic patient-reported outcome) systems. These platforms can deliver brief, tailored assessments—potentially informed by MCAT principles—either remotely before appointments or during clinic visits. By selecting only the most informative items per domain, ePRO systems reduce patient burden while providing clinicians with automated scoring and concise, interpretable summaries [74, 75]. Although developing and calibrating an item bank requires an initial investment, such costs can be integrated within broader digital-health initiatives and are likely to be offset over time by gains in efficiency, reduced administration workload, fewer missing data, and streamlined documentation [76-78]’.

25. *You say ‘Future research should continue to engage PwMS in the development and refinement of HRQoL instruments to ensure that these measures are meaningful and actionable.’ How about remodeling already existing questionnaires? Please discuss.*

We thank the Reviewer for this thoughtful suggestion. We agree that revising and updating existing MS-specific HRQoL questionnaires represents a valuable and feasible strategy to enhance their conceptual clarity and patient relevance. In response to this comment, we have expanded the Discussion to explicitly address this point, as follows: ‘In addition to supporting future adaptive or item-bank–based developments, the conceptual framework generated here can also guide the systematic revision of existing MS-specific HRQoL questionnaires. Many instruments differ in domain structure, conceptual granularity, and coverage of patient-prioritized areas, leading to inconsistencies in measurement. Using the refined domains and subdomains identified in this study as a reference, existing instruments could be remodeled to enhance conceptual alignment, reduce redundancy, and incorporate underrepresented yet highly relevant aspects such as work participation, financial concerns, self-perception, and contextual influences. This represents a complementary and immediately feasible pathway for improving HRQoL measurement, particularly in settings where digital adaptive technologies are not yet available’.

***Reviewer #2: Main concerns***

1. *You state FGMs were conducted in April 2024 across three enrolling centers (FINCB, AISM/FISM Genoa, UNICA Cagliari), but list approvals as:
   UNITO: 21/11/2023
   FINCB: 30/10/2023
   AISM: 19/12/2024 (after April 2024)
   UNICA: 11/03/2025 (long after April 2024)
   If any participants were recruited/consented at AISM or UNICA before those centers had approvals, this is noncompliant with PLOS ethical standards.
   Please provide a table clarifying, per site, (a) approval date, (b) site start date of any recruitment or FGM participation, (c) N recruited per site, and (d) confirmation that no human subjects activities occurred before site-specific approval. If the listed dates are typographical, correct them and supply documentation. If activities occurred pre-approval at any site, the manuscript should be withdrawn or rejected on ethical grounds.*
   We sincerely thank the Reviewer for this careful observation and apologize for the typographical error in the originally submitted version. The correct timeline is that participant recruitment occurred between January and March 2025, and the focus group meetings (FGMs) were conducted in April 2025.

Each participating center began recruitment only after obtaining its local ethical approval. The latest approval (UNICA, 11 March 2025) preceded the start of participant recruitment at that site. No participant-related activity took place before site-specific approval at any center.

We have corrected the dates in the Methods section accordingly and confirm that the study fully complies with PLOS ethical standards and local regulatory requirements.

As requested, we provide below a summary table detailing the ethical approval and recruitment timeline for each center.

| **Center** | **Ethics approval date** | **Recruitment / FGM start date** | **N recruited** | **Compliance confirmation** |
| --- | --- | --- | --- | --- |
| University of Turin (UNITO) | 21 Nov 2023 | Not recruiting center | Not recruiting center | Not recruiting center |
| Fondazione IRCCS Istituto Neurologico Carlo Besta (FINCB) | 30 Oct 2023 | Jan 2025 | 2 PwMS / 12 HPs | No human subjects activities occurred before approval |
| AISM/FISM Genoa | 19 Dec 2024 | Feb 2025 | 6 PwMS / 4 HPs | No human subjects activities occurred before approval |
| University of Cagliari (UNICA) | 11 Mar 2025 | Mar 2025 | 6 PwMS / 2 HPs | No human subjects activities occurred before approval |

FGM, focus group meeting; PwMS, people with multiple sclerosis; HPs, healthcare professionals.

*Systematic Review*

2. *Methods transparency
   Inclusion/exclusion logic is unclear and may be internally inconsistent: you state studies using an instrument “solely as an outcome measure” were excluded, yet you later include hundreds of “references related to” nine instruments.*

We thank very much this Reviewer for their comment. Please see the reply to point 3 below.

3. *Clarify exactly which study types were included and why.*
   We thank the Reviewer for their comment. We have now clarified in the Methods the study types included in our systematic review, as follows: ‘We considered all studies that provided information relevant to the conceptualization and use of HRQoL instruments in PwMS. Specifically, we included: observational and interventional studies employing HRQoL instruments as primary or secondary outcomes; and development and methodological papers describing instrument structure or domain composition’.
4. *No quality appraisal of measurement instruments is reported (e.g., COSMIN standards for content validity, structural validity, reliability).
   Mapping domains without evaluating instrument quality risks biasing the domain framework toward historically popular but potentially weaker tools.*
   We thank the Reviewer for their valuable comment. We agree that quality appraisal is essential when assessing measurement properties. However, the purpose of this phase of the INITIALISE project was not to evaluate the psychometric quality of instruments, but to map all HRQoL domains represented across the literature.

We did, however, refer to the COSMIN framework when developing our search strategy, as it provided a structured approach to identifying studies describing the use of HRQoL questionnaires in people with MS. Our focus was therefore on how instruments were conceptually applied and which domains they covered, rather than on their measurement properties. This approach was deliberately broad to ensure that both well-established and less frequently used instruments contributed to the mapping, allowing us to capture the most comprehensive HRQoL conceptual structure possible. A formal COSMIN-based evaluation of measurement properties is being conducted in a parallel phase of the INITIALISE project.

5. *The mapping relies heavily on Leidy et al. (an older framework) and subjective consolidation. Consider triangulating with Wilson & Cleary, WHO ICF, or updated HRQoL frameworks to enhance conceptual robustness.*
   We thank the reviewer for their comment. We acknowledge that the framework proposed by Leidy et al. (1999) is an earlier model; however, our intention was not to pursue additional conceptual triangulation or theoretical robustness, but to ensure inclusion of all domains relevant to people with MS and to structure them in a way that is operationally useful for measurement development.

Given that one of the overarching aims of the INITIALISE project is the creation of an item bank for multidimensional computer adaptive testing (MCAT), as well as studies designed to capture and rank the priorities that patients assign to these domains, we selected a model that is both explanatory and efficient for item generation and calibration. The Leidy framework provides a clear link between symptoms, functioning, and HRQoL–features that make it particularly suitable for guiding future adaptive measurement tools.

6. *Participant counts are unclear. Text mentions median ages/EDSS but not exact Ns; IDs in quotes range up to “18H” yet you describe 2 FGMs with HPs only. Provide exact numbers.*

We thank the Reviewer for this comment. We have now improved the “FGMs participants and setting” section as follows: ‘Between January 1, 2025 and March 31, 2025 we enrolled 18 PwMS and 20 HPs, across all study centers. Four online focus group meetings (FGMs) were conducted in April 2025, two with PwMS and two with HPs. The average duration of the FGMs was 144 minutes (SD 9.9) with PwMS, and 132 minutes (SD 14.1) with HPs. Among those enrolled, five PwMS and two HPs were unable to participate due to medical or logistical reasons.

PwMS (7 women, 7 men) had a median age of 52.5 years (range 36–64). EDSS scores ranged from 1.5 to 9.0 (median 4.0), and disease duration ranged from 5 months to 42 years (median 17). Half had a relapsing-remitting course and half a progressive form. Participants were recruited from Milano, Cagliari, and Genova, attending FGM 2 (n = 9) or FGM 3 (n = 5).

HPs (15 women, 3 men) had a median age of 50 years (range 30–65) and 3–35 years of experience in MS care (mean 19.6). They had followed a median of 100 PwMS in the past six months (range 12–600) and were employed across MS centres, rehabilitation centres, and AISM facilities, representing roles such as neurologists, nurses, physiotherapists, psychologists, and social workers. HPs participated in FGM 1 (n = 10) and FGM 4 (n = 8)’.

***Reviewer #3***

*Dear Authors thank you for your paper.
I have enjoyed reading it and recommend reviewing some of the comments I have made in the attached document.
Overall, the paper offers a reasonable perspective on expanding the HRQoL domains, however, stronger justification for certain aspects of the paper is required eg: what is the current state of the Outcome measure landscape in MS etc.*

*Introduction*

1. *[the most frequent cause of neurological disability in] young adults – Age range for young adults?*

The age range for young adults is between 20 and 40 years, as stated in the Introduction.

2. *[Fatigue, pain, depression, and cognitive dysfunction are some of the multiple symptoms impaired by MS that can affect various areas of life for people with MS (PwMS), such as the ability to work and/or perform activities of daily living] but are difficult for healthcare providers to quantify – In what way is it difficult?*

We thank this Reviewer for their comment. The reason is because these symptoms are subjective, fluctuating, and lack objective clinical markers, making them hard to measure consistently or compare across patients. We have slightly revised the text, as follows: ‘Fatigue, pain, depression, and cognitive dysfunction are some of the multiple symptoms impaired by MS that can affect various areas of life for people with MS (PwMS), such as the ability to work and/or perform activities of daily living, but are difficult for healthcare providers to quantify because these symptoms are subjective, fluctuating, and lack objective clinical markers, making them hard to measure consistently or compare across patients [8]’.

3. *[The aim of HRQoL instruments is to provide] a more complete […] – In what way?*

We thank this Reviewer for their comment. We meant that the aim of HRQoL instruments is to provide a more comprehensive and personalized way to measure the impact of the disease […]. To improve clarity, we have now slightly revised the text, accordingly.

4. *[This is due] to potential logistical and financial issues […] - It is more than the logistical and financial issues- what about the hierarchy that still exists in medicine; patient-dr interaction, etc.*

We thank this Reviewer for their comment. We agree that, beyond logistical and financial barriers, cultural and relational factors, such as hierarchical structures in medicine and asymmetrical patient–clinician interactions may also limit the integration of HRQoL instruments into clinical practice. We have now slightly revised the text and added a supporting reference, as follows: ‘Moreover, cultural and relational factors, such as hierarchical structures in medicine and asymmetrical patient–clinician relationships, may also hinder the routine use of HRQoL tools [16].’

5. *[…] informed by the patient voice - How did the patient voice inform your research?*

The patient voice informed our research in two ways. First, the patients were included in the Engagement Coordination Team, a multidisciplinary team representing key stakeholders for the study (researchers, clinicians, experts in the Multiact model, and importantly, patients – in our case, PwMS) who collaborated in the research to capture the disease experience and translate it into scientific knowledge, with the aim of improving, recalibrating, and potentially modifying the research project being undertaken [MULTI-ACT Consortium. Guidelines for patient engagement in health research & innovation. 2020 (https://www.multiact.eu/wp-content/uploads/2020/05/MULTI-ACT-Patient-Engagement-Guidelines-Short.pdf)]. Second, the patients were involved in the focus group meetings.

6. *[However, in the MS context, the perspective of PwMS has often been overlooked in the] development of existing PROs. Can you give examples?*

Following this Reviewer’s comment, we have now included a few examples, as follows: ‘However, in the MS context, the perspective of PwMS has often been overlooked in the development of existing PROs, such as for example the Multiple Sclerosis Quality of Life-54 items (MSQoL-54) [43], as well as the Functional Assessment of Multiple Sclerosis (FILMS) [44], and the RAYS [45] instruments.

7. *[(2) to discuss the identified HRQoL domains with PwMS and eventually supplement them with] additional relevant domains. - more around what exists in MS at the moment would have been good to see here.*

Following the Reviewer’s suggestion, we have now provided a clearer overview of the HRQoL domains already described in MS-specific instruments strengthening the rationale for the second study objective. Accordingly, we slightly revised the end of the Introduction, as follows: ‘Existing MS-specific HRQoL instruments—such as the MSQOL-54, MSIS-29, MusiQoL, and FAMS—typically assess domains including physical functioning, fatigue, pain, cognition, emotional well-being, and social relationships. However, these instruments differ in scope, structure, and conceptual coverage, and may not fully align with the domains that PwMS describe as most meaningful, such as self-perception, social participation, work-related challenges, and contextual or relational influences on daily life [46]. These underrepresented areas suggest that the current conceptualization of HRQoL in MS may not fully reflect patients’ lived experiences.

By integrating evidence from the literature with qualitative insights from PwMS and health professionals (HPs), the present study aims to refine and expand the conceptual framework of HRQoL in MS, thereby enhancing its content validity and ensuring stronger patient relevance for future adaptive measurement development’.

*Methods / Systematic review*

8. *[…] instruments solely as an outcome measure - not clear on the reasoning for this*

Please, see point 2 of Reviewer 2 above.

9. *FGM conduction – conduct*

Thanks, we have now corrected the typo.

10. *Results / Systematic review - PRISMA flowchart would be good to be included here*

The PRISMA flowchart has been already included as S4 File in the original submission.

11. *HRQOL domain mapping - which framework did you map the domains onto- I may have missed it, but it is not clear which one you used*

We have used the framework by Leidy et al., (1999) who defined HRQoL as "the subjective perception of the impact of disease and treatment across the physical, psychological, social, and well-being” [Leidy NK, Revicki DA, Genesté B. Recommendations for evaluating the validity of quality of life claims for labeling and promotion. Value Health. 1999;2(2):113-27. doi:10.1046/j.1524-4733.1999.02210.x.]. Please, see also point 5 of Reviewer 2.

12. *INITIALISE – Missing info*

INITIALISE is the name of our project. We have now included it in the list of abbreviations.

13. *A diagram showing the HRQoL via SR and FGM would make the information more visible*

We thank the Reviewer for this helpful suggestion. We agree that a visual summary can enhance readability and facilitate understanding of the findings.

We have revised the manuscript by adding a detailed analysis reported in Tables 2- 4 comparing the domains assessed by existing MS-specific HRQoL questionnaires with the qualitative findings. This comparison is presented comprehensively in Tables 2–4. As a result of this additional analysis, we have updated the Methods, Results, and Discussion sections to reflect these refinements and provide a clearer, more nuanced interpretation of the data.

We believe that this approach, with Table 1 summarizing the overlap and Tables 2–4 detailing the questionnaire–qualitative comparison, provides the most complete and informative representation of our findings.

14. *Discussion - Elements of the discussion are a repetition of the results- can you find a way to minimise having the results in this section?*

We thank the Reviewer for this valuable suggestion. Following their recommendation, we have completely revised the Discussion with the aim of making it more concise, focused, and impactful. In the revised version, we minimized repetition of the Results section and emphasized interpretation, synthesis, and implications of the findings.

Importantly, we also incorporated a detailed, direct comparison between the findings from the systematic review of HRQoL questionnaires and the qualitative insights from the focus group meetings. This addition strengthens the discussion by highlighting gaps in current instruments and by showing how our study refines and extends the understanding of HRQoL domains relevant to PwMS.

We believe that these revisions have substantially improved the clarity, focus, and interpretive value of the Discussion section.

15. *f.e. - not sure what this abbreviation is*

We have now made explicit that abbreviation, which stands for ‘for example’.

16. *The SR HRQoL instruments would have had some aspect of pain in them- it would be useful to identify this*

We thank the Reviewer for this comment. We agree that pain represents an important aspect of HRQoL in MS and was consistently included among the physical domains across the HRQoL instruments identified in the systematic review.

The newly conducted detailed comparison between qualitative findings and existing HRQoL questionnaires allowed us to highlight this aspect more clearly. In the Results section, we now specify that: ‘However, fine-grained distinctions emerging from the qualitative analysis—such as the separation between acute and chronic pain, or between lower- vs. upper-limb mobility impairments—were often not reflected in instrument items, which were frequently non-specific or mapped simultaneously to multiple subcategories.’

This level of detail is also presented in Table 2, which illustrates how specific subdomains identified in the focus group meetings relate (or fail to relate) to items in existing HRQoL instruments. These revisions emphasize gaps in current measurement tools and the added value of incorporating patient-reported qualitative insights.

17. *[Cognitive decline in MS can severely hinder daily functioning and is frequently] underappreciated in clinical practice - In what way - it is one of the areas that is normally routinely assessed*

We thank the Reviewer for their comment. We agree that cognitive impairment is increasingly recognized and routinely assessed in MS clinical practice, particularly in MS centers. Our statement was intended to highlight that, despite its assessment, the functional and psychosocial consequences of cognitive decline—such as its impact on work ability, autonomy, and social participation—are often underappreciated or under addressed in standard care and HRQoL evaluations. We have now slightly revised the sentence, as follows: ‘Cognitive decline in MS can severely hinder daily functioning. Although it is increasingly recognized and routinely assessed in clinical practice, its broader consequences on work ability, autonomy, and social participation are often under addressed [67].’

18. *[…] cultural, socioeconomic, or geographical backgrounds - What did the researchers do to minimise this exclusion?*

We purposively sampled the participants to ensure diversity in geographic location, gender, education, and (for PwMS) disease severity.

19. *Physical, Psychological and Social domains identified in […] - is it the additions to these core areas rather than the domains themselves?*

We thank the Reviewer for this important comment. We agree that the Physical, Psychological, and Social domains are well-established in MS HRQoL literature, and our study does not claim them as novel. As noted in our response to Comment 11 (Reviewer 1), the contribution of our work lies in refining and extending these domains through qualitative insights, identifying additional subdomains such as work-related challenges, financial concerns, contextual influences, and aspects of self-perception.

To clarify this, we revised the manuscript (see also points 6 and 23 to Reviewer 1), restructuring sections for clarity, streamlining content, and introducing a dedicated methodological section, “Cross-Mapping of HRQoL Instruments and Qualitative Findings”, which compares qualitative findings with existing instruments. Results of this comparison are presented in the Results section and informed revisions to the Discussion.

Thus, the novelty lies in the added conceptual granularity and identification of measurement gaps, rather than the identification of new overarching domains.
