# Supplementary material for: Quality of life domains revised by people with multiple sclerosis and healthcare professionals for adaptive measure development
Source: PLoS One. 2026 Jun 11;21(6):e0349034. doi: 10.1371/journal.pone.0349034 (PMC13257964; doi:10.1371/journal.pone.0349034)
Supplement: Attachment — Submitted filename: Response to reviewers_9-3-26.docx [file pone.0349034.s009.docx]

**Reviewer #4:**

*This is a well-executed and carefully revised manuscript. The authors have addressed the comments from previous reviewers in a thoughtful and transparent manner, and the methodological approach is appropriate and rigorous for the stated aims. The sequential integration of a systematic review, qualitative focus groups, and cross-mapping of domains provides a solid foundation for refining HRQoL constructs in multiple sclerosis. Overall, the study is methodologically sound and close to being suitable for publication.*
Reply: We thank very much the Reviewer for their appreciation.

*I have a few minor comments that are intended to further strengthen the conceptual clarity and methodological transparency of an otherwise strong manuscript.
1) Treatment Burden concept: While treatment-related aspects are included within the physical domain (e.g., impact of DMTs on the body), the broader concept of treatment burden could be more explicitly acknowledged. In chronic diseases such as multiple sclerosis, treatment burden extends beyond biological effects and often encompasses cognitive, emotional, organizational, financial, and social demands, which may influence HRQoL across physical, psychological, and social domains simultaneously. I suggest briefly acknowledging treatment burden in the Discussion as a cross-cutting factor that modifies HRQoL across domains, even if it is not proposed as a separate domain. This would further strengthen the patient-centered perspective of the framework and align well with the manuscript’s emphasis on adaptive and personalized HRQoL assessment.*
Reply: We thank the Reviewer for this insightful comment. Following the Reviewer’s suggestion, we have now more explicitly acknowledged the broader concept of treatment burden in the manuscript. First, we strengthened this aspect in the Results section (Psychological Domain), where it was previously less explicitly represented. In particular, we added the following text to highlight the psychological impact associated with the ongoing management of the disease:
“Participants also highlighted that the ongoing management of the disease–including therapies, diagnostic tests, and regular medical appointments–can itself generate psychological strain: ‘One aspect that hasn't been mentioned is the impact–not just physical, but also the effects of therapies and the planning of the entire diagnostic process on daily life… individuals suddenly feel they are in need of support from others and require assistance from healthcare professionals.’ (ID: 6H).”

Second, we revised the Discussion to explicitly acknowledge treatment burden as a cross-cutting factor influencing HRQoL across domains. In the section *Integrating Implications Across Domains*, we added the following statement: “In addition, our findings suggest that treatment burden—including the cognitive, emotional, and organizational demands associated with therapies, medical appointments, and disease monitoring—may act as a cross-cutting factor influencing HRQoL simultaneously across physical, psychological, and social domains.”

These additions aim to better reflect the multidimensional nature of treatment burden and its relevance for patient-centered and adaptive HRQoL assessment in PwMS.

*2) Role of social support: Although social support is appropriately included within the social domain, its buffering role in mitigating treatment burden, disability, and psychosocial stress could be more explicitly highlighted. This may be particularly relevant when discussing future adaptive or digital HRQoL tools, where social resources can substantially shape patients’ capacity to cope with disease and treatment demands.*
Reply: We thank the Reviewer for their comment. According to Leidy’s definition of HRQOL as the “subjective perception of the impact of disease and treatment across the physical, psychological, social functioning and well-being” [ref #10], our objective was to explore how MS affects each of these core HRQOL domains. Within this conceptual framework, social support represents a key component of the Social Domain (Table 4). In the previous version of the manuscript, we had also included a separate theme, called ‘Factors influencing HRQOL’, including the following sub-themes: Personal Factors; Health-Related Factors; Life-Related Factors; Relational Factors; and Contextual Factors.

Following the Reviewers’ suggestions, we reconsidered the alignment of this section with the primary aim of the study. As our objective was specifically focused on describing the impact of multiple sclerosis on HRQoL domains, we agreed that this additional thematic area extended beyond the core scope of the manuscript. For this reason, we removed the “Factors Influencing HRQoL” subsection, which also contributed to improving the clarity and conciseness of the text.

*3) Qualitative rigor and saturation: The qualitative methodology is clearly described and robust. For completeness, the authors may consider explicitly stating whether thematic saturation was considered and achieved during the focus group process, as this is commonly expected in qualitative research reporting.*
Reply: We thank the Reviewer for this comment. Data saturation was discussed and results were reported in the Consolidated criteria for reporting qualitative studies (COREQ) checklist (S3 File), as follows: “Yes. Data saturation was discussed during the analysis process. After conducting four focus group meetings, researchers observed recurring themes and minimal emergence of new concepts, suggesting that thematic saturation had been reached. The decision to stop data collection was based on the redundancy of the information and the consistency of themes across groups.”

*Given that the focus group guides were informed by domains identified in existing HRQoL instruments, it may be helpful to briefly acknowledge the potential for a framing effect, even though this was mitigated by the combined deductive–inductive coding approach.*

Reply: We thank the Reviewer for this thoughtful comment. The focus group guides were intentionally informed by domains identified in existing HRQoL instruments, as the aim of the study was to build on and situate our findings within the established literature. This deductive grounding was considered important to ensure conceptual alignment with prior work and to facilitate comparability across studies. At the same time, we acknowledge the potential for a framing effect inherent in this approach. To mitigate this risk, we adopted a combined deductive–inductive coding strategy. While initial codes were informed by existing frameworks, the analysis remained open to the identification of themes and subthemes emerging directly from the data. In our view, this inductive component ensured that novel, unanticipated aspects of participants’ experiences could be recognized and incorporated into the findings, even when not captured by existing HRQoL instruments.

*Additionally, a short comment on the transferability of findings beyond the Italian healthcare context would further enhance methodological transparency.*
Reply: Following the Reviewer’s suggestion, we have now slightly revised the Limitations, as follows: ‘Transferability of our findings beyond the Italian healthcare system requires further investigation’.

*4) Caregiver perspectives - The absence of caregivers or informal support persons is appropriately mentioned as a limitation. Given the relevance of social support and treatment burden, the authors may wish to emphasize that caregiver perspectives represent an important methodological and conceptual direction for future research.*
Reply: We agree with this Reviewer. Following their suggestion, we have slightly revised the Limitations, as follows: ‘Including caregivers in future research would allow for a more comprehensive and multidimensional understanding of these aspects, enriching the interpretation of HRQoL through complementary perspectives and strengthening the overall validity of the findings’.
