# Supplementary material for: Quality of life domains revised by people with multiple sclerosis and healthcare professionals for adaptive measure development
Source: PLoS One. 2026 Jun 11;21(6):e0349034. doi: 10.1371/journal.pone.0349034 (PMC13257964; doi:10.1371/journal.pone.0349034)
Supplement: Peer review history

# Peer review: Decision Letter 0

**DOI:** 10.1371/journal.pone.0349034.r001

**Authors:** Homa Seyedmirzaei

23 Sep 2025

PONE-D-25-43699

Quality of life domains revised by people with multiple sclerosis and healthcare professionals for adaptive measure development

PLOS ONE

Dear Dr. Giordano,

Thank you for submitting your manuscript to PLOS ONE. After careful consideration, we feel that it has merit but does not fully meet PLOS ONE’s publication criteria as it currently stands. Therefore, we invite you to submit a revised version of the manuscript that addresses the points raised during the review process.

Please submit your revised manuscript by Nov 07 2025 11:59PM. If you will need more time than this to complete your revisions, please reply to this message or contact the journal office at plosone@plos.org. When you're ready to submit your revision, log on to https://www.editorialmanager.com/pone/ and select the 'Submissions Needing Revision' folder to locate your manuscript file.

Please include the following items when submitting your revised manuscript:

- A rebuttal letter that responds to each point raised by the academic editor and reviewer(s). You should upload this letter as a separate file labeled 'Response to Reviewers'.
- A marked-up copy of your manuscript that highlights changes made to the original version. You should upload this as a separate file labeled 'Revised Manuscript with Track Changes'.
- An unmarked version of your revised paper without tracked changes. You should upload this as a separate file labeled 'Manuscript'.

If you would like to make changes to your financial disclosure, please include your updated statement in your cover letter. Guidelines for resubmitting your figure files are available below the reviewer comments at the end of this letter.

If applicable, we recommend that you deposit your laboratory protocols in protocols.io to enhance the reproducibility of your results. Protocols.io assigns your protocol its own identifier (DOI) so that it can be cited independently in the future. For instructions see: https://journals.plos.org/plosone/s/submission-guidelines#loc-laboratory-protocols. Additionally, PLOS ONE offers an option for publishing peer-reviewed Lab Protocol articles, which describe protocols hosted on protocols.io. Read more information on sharing protocols at https://plos.org/protocols?utm_medium=editorial-email&utm_source=authorletters&utm_campaign=protocols.

We look forward to receiving your revised manuscript.

Kind regards,

Homa Seyedmirzaei, M.D

Academic Editor

PLOS ONE

Journal Requirements:

When submitting your revision, we need you to address these additional requirements.

1. Please ensure that your manuscript meets PLOS ONE's style requirements, including those for file naming. The PLOS ONE style templates can be found at

https://journals.plos.org/plosone/s/file?id=wjVg/PLOSOne_formatting_sample_main_body.pdf and

https://journals.plos.org/plosone/s/file?id=ba62/PLOSOne_formatting_sample_title_authors_affiliations.pdf

2. Thank you for stating the following in your Competing Interests section: “None”

Please complete your Competing Interests on the online submission form to state any Competing Interests. If you have no competing interests, please state "The authors have declared that no competing interests exist.", as detailed online in our guide for authors at http://journals.plos.org/plosone/s/submit-now

This information should be included in your cover letter; we will change the online submission form on your behalf.

3. One of the noted authors is a group or consortium INITIALISE project. In addition to naming the author group, please list the individual authors and affiliations within this group in the acknowledgments section of your manuscript. Please also indicate clearly a lead author for this group along with a contact email address.

4. If the reviewer comments include a recommendation to cite specific previously published works, please review and evaluate these publications to determine whether they are relevant and should be cited. There is no requirement to cite these works unless the editor has indicated otherwise.

[Note: HTML markup is below. Please do not edit.]

Reviewers' comments:

Reviewer's Responses to Questions

**Comments to the Author**

1. Is the manuscript technically sound, and do the data support the conclusions?

The manuscript must describe a technically sound piece of scientific research with data that supports the conclusions. Experiments must have been conducted rigorously, with appropriate controls, replication, and sample sizes. The conclusions must be drawn appropriately based on the data presented.

Reviewer #1: Partly

Reviewer #2: Yes

Reviewer #3: Partly

**********

2. Has the statistical analysis been performed appropriately and rigorously?

Reviewer #1: N/A

Reviewer #2: N/A

Reviewer #3: N/A

**********

3. Have the authors made all data underlying the findings in their manuscript fully available?

The PLOS Data policy requires authors to make all data underlying the findings described in their manuscript fully available without restriction, with rare exception (please refer to the Data Availability Statement in the manuscript PDF file). The data should be provided as part of the manuscript or its supporting information, or deposited to a public repository. For example, in addition to summary statistics, the data points behind means, medians and variance measures should be available. If there are restrictions on publicly sharing data—e.g. participant privacy or use of data from a third party—those must be specified.

Reviewer #1: No

Reviewer #2: No

Reviewer #3: Yes

**********

4. Is the manuscript presented in an intelligible fashion and written in standard English?

PLOS ONE does not copyedit accepted manuscripts, so the language in submitted articles must be clear, correct, and unambiguous. Any typographical or grammatical errors should be corrected at revision, so please note any specific errors here.

Reviewer #1: Yes

Reviewer #2: Yes

Reviewer #3: Yes

**********

5. Review Comments to the Author

Please use the space provided to explain your answers to the questions above. You may also include additional comments for the author, including concerns about dual publication, research ethics, or publication ethics. (Please upload your review as an attachment if it exceeds 20,000 characters)

Reviewer #1: Thank you for inviting me to review your manuscript:

Great project, very precise implementation of research plan

I have some questions and comments for your manuscript:

The WHOQolbref measures quality of life (QoL) on 4 domains – Physical, psychological, social, environmental QoL. Was this questionnaire never used to measure QoL in MS?

In my understanding, you tried to find quantitative instruments used in research that measure QoL in MS.

I could not find what these instruments did not include and what they should have measured in addition.

You state that new instruments should be developed. I wonder if complementing already existing ones would be possible.

Was it possible for all potential participants to meet virtually? Did all have access to the internet? If not, was there a plan how they could have been assisted?

(e.g. drive home to them and offer proper assistance?)

Did participants sign a consent form for being audiotaped?

Please structure your paper that it is easier to read and to follow.

It is a very long manuscript.

Please try to make it shorter, at the same time clearer.

Table 1: I would have expected more domains.

Please add list of abbreviations

Persons with MS were included and health professionals. Support persons were not included. They can provide great information regarding the QoL of PwMS. They should have been included in the focus groups.

If you want to submit this as a paper, be aware that it should be much shorter.

You can put information, which is maybe to detailed, to the Appendix. Not everything has to be in the main paper.

You identified physical, psychological and social domains as important.

We already know this from former qualitative and quantitative research.

Please describe your additional findings more focused and why your study was so important and a novelty.

You state ‘The growing interest in CAT instruments in HRQoL research has led to the development of a range of CAT instruments by the Patient-Reported Outcomes Measurement Information System (PROMIS) supported by the National Institute of Health [20], which currently offers 3 to 10 items for domains such as fatigue, physical functioning, pain, depression, anxiety, and social functioning (www.nihpromis.org). For the assessment of multidimensional constructs with correlated dimensions, the Multidimensional Computer Adaptive Testing (MCAT) approach is more appropriate, but, as far as we know, there is few evidence of HRQoL instruments using such a methodology to fixed-length HRQOL questionnaires, including the MS domain [21-24]. To address this gap, we took a comprehensive approach based on a literature review and informed by the patient voice to identify relevant HRQoL dimensions that ensure both content validity and clinical utility of the assessment [20,25,26].’

My question: did you use MCAT by facilitating the focus groups online?

I don’t understand why identifying quantative questionnaires used was necessary first before the qualitative focus group discussions.

Please clarify.

You state that your goal was

‘ 1. to conduct a systematic literature review to identify the most relevant HRQoL domains in MS; 2) to discuss the identified HRQoL domains with PwMS and eventually supplement them with additional relevant domains.’

What additional categories would you add to understanding HRQOL.

You state ‘To identify additional studies, we also reviewed published systematic reviews and the reference lists of the selected articles’. Good job!

You picked PwMS who should be in the FG interviews? Is this correct? Would it have been possible you would have allowed random participants?

Please specify ‘Investigators at each participating centre identified potentially eligible PwMS from their database and invited them to participate in the study’.

Regarding Inclusion/Exclusion Criteria:

Try to say in Methods/Study Design/Study Participants who was included and what the inclusion criteria were.

FGM Conduction:

that paragraph could be said in 2 sentences.

Try to reduce text.

Try to create one chapter called Methods and Study Design and describe all Methods used in this study.

Describe under Analysis all Methods you used.

You have Methods for the focus groups and methods for identifying literature on the search engines.

Under results you already describe what you identified with your methods used. That’s good.

For the qualitative study don’t mix participants and setting with the results.

Under results present all results, under Methods present all Methods used (thus for your focus groups as well)

Why is cognitive function under psychological and not under physical?

Is this because it was discussed in the focus group interviews? Please specify.

Why was it necessary to identify all different quantitative questionnaires first before you used started the focus group discussions?

The qualitative results are totally independent.

Is this correct? Would it have made more sense to conduct the focus group first and do the questionnaires then? Please specify.

Your focus group interviews are great. No novelty was identified. If so, please specify and explain the advantages of mixing PwMS and Clinical Professionals in the focus group interviews.

No changes for questionnaires were discussed based on the results of your focus groups.

You say ‘the necessity of a multidimensional approach to HRQoL assessment in MS.’ But you don’t make suggestions how this should look like. You say ‘it seems particularly promising to integrate the identified HRQoL domains into a robust item bank suitable for MCAT. This approach would enable more personalized, efficient and accurate HRQoL assessments in PwMS and improve the ability to capture individual variation while reducing respondent burden, improving measurement precision and supporting both research and clinical decision-making’.

But how can that be done in daily clinical settings? Who would cover the costs? The extra time needed? Please try to discuss in the Discussion chapter.

You say ‘Future research should continue to engage PwMS in the development and refinement of HRQoL instruments to ensure that these measures are meaningful and actionable.’ How about remodeling already existing questionnaires? Please discuss.

Reviewer #2: Main concerns

1 You state FGMs were conducted April 2024 across three enrolling centers (FINCB, AISM/FISM Genoa, UNICA Cagliari), but list approvals as:

UNITO: 21/11/2023

FINCB: 30/10/2023

AISM: 19/12/2024 (after April 2024)

UNICA: 11/03/2025 (long after April 2024)

If any participants were recruited/consented at AISM or UNICA before those centers had approvals, this is noncompliant with PLOS ethical standards.

Please provide a table clarifying, per site, (a) approval date, (b) site start date of any recruitment or FGM participation, (c) N recruited per site, and (d) confirmation that no human subjects activities occurred before site-specific approval. If the listed dates are typographical, correct them and supply documentation. If activities occurred pre-approval at any site, the manuscript should be withdrawn or rejected on ethical grounds.

2 Systematic Review—methods transparency

Inclusion/exclusion logic is unclear and may be internally inconsistent: you state studies using an instrument “solely as an outcome measure” were excluded, yet you later include hundreds of “references related to” nine instruments. Clarify exactly which study types were included and why.

No quality appraisal of measurement instruments is reported (e.g., COSMIN standards for content validity, structural validity, reliability).

Mapping domains without evaluating instrument quality risks biasing the domain framework toward historically popular but potentially weaker tools.

3 The mapping relies heavily on Leidy et al. (an older framework) and subjective consolidation. Consider triangulating with Wilson & Cleary, WHO ICF, or updated HRQoL frameworks to enhance conceptual robustness.

4 Participant counts are unclear. Text mentions median ages/EDSS but not exact Ns; IDs in quotes range up to “18H” yet you describe 2 FGMs with HPs only. Provide exact numbers

Reviewer #3: Dear Authors

Thank you for your paper.

I have enjoyed reading it and recommend reviewing some of the comments I have made in the attached document.

Overall, the paper offers a reasonable perspective on expanding the HRQoL domains, however, stronger justification for certain aspects of the paper is required eg: what is the current state of the Outcome measure landscape in MS etc

**********

6. PLOS authors have the option to publish the peer review history of their article (what does this mean?). If published, this will include your full peer review and any attached files.

If you choose “no”, your identity will remain anonymous but your review may still be made public.

**Do you want your identity to be public for this peer review?** For information about this choice, including consent withdrawal, please see our Privacy Policy.

Reviewer #1: No

Reviewer #2: No

Reviewer #3: **Yes:** Adine Adonis

**********

[NOTE: If reviewer comments were submitted as an attachment file, they will be attached to this email and accessible via the submission site. Please log into your account, locate the manuscript record, and check for the action link "View Attachments". If this link does not appear, there are no attachment files.]

While revising your submission, please upload your figure files to the Preflight Analysis and Conversion Engine (PACE) digital diagnostic tool, https://pacev2.apexcovantage.com/. PACE helps ensure that figures meet PLOS requirements. To use PACE, you must first register as a user. Registration is free. Then, login and navigate to the UPLOAD tab, where you will find detailed instructions on how to use the tool. If you encounter any issues or have any questions when using PACE, please email PLOS at figures@plos.org. Please note that Supporting Information files do not need this step.

Attachment Submitted filename: download_Reviewed.pdf

# Author comment: Author response to Decision Letter 1

**DOI:** 10.1371/journal.pone.0349034.r002

6 Dec 2025

Response to Reviewers

We sincerely thank the Reviewers for their valuable comments, which have greatly helped us to improve the quality and clarity of the manuscript.

Reviewer #1

Thank you for inviting me to review your manuscript:

Great project, very precise implementation of research plan

We thank very much this Reviewer for their appreciation of our project.

I have some questions and comments for your manuscript

1. The WHOQol-BREF measures quality of life (QoL) on 4 domains – Physical, psychological, social, environmental QoL. Was this questionnaire never used to measure QoL in MS?

We thank the reviewer for this comment. Indeed, this questionnaire was used to measure QoL in MS [e.g., Pomeroy et al. (2020). The WHOQOL‑BREF: a modern psychometric evaluation of its internal construct validity in people with multiple sclerosis. Quality of Life Research, 29, 1961–1972. https://doi.org/10.1007/s11136-020-02463-z]. However, as stated in the Methods, our scope was to ‘collect all MS-specific instruments that have been used to measure HRQoL in adults with MS’. So, we did not consider the WHOQOL-BREF as it is a generic HRQOL measure.

2. In my understanding, you tried to find quantitative instruments used in research that measure QoL in MS.

I could not find what these instruments did not include and what they should have measured in addition.

We thank the Reviewer for their comment. We addressed this point by expanding the final paragraph of the Introduction (please, see also our response to point 7 of Reviewer 3), where we now provide a clearer overview of the HRQoL domains already described in MS-specific instruments, as follows: ‘Existing MS-specific HRQoL instruments—such as the MSQoL-54, MSIS-29, MusiQoL, and FAMS—typically assess domains including physical functioning, fatigue, pain, cognition, emotional well-being, and social relationships. However, these instruments differ in scope, structure, and conceptual coverage, and may not fully align with the domains that PwMS describe as most meaningful, such as self-perception, social participation, work-related challenges, and contextual or relational influences on daily life [46]. These underrepresented areas suggest that the current conceptualization of HRQoL in MS may not fully reflect patients’ lived experiences.

By integrating evidence from the literature with qualitative insights from PwMS and health professionals (HPs), the present study aims to refine and expand the conceptual framework of HRQoL in MS, thereby enhancing its content validity and ensuring stronger patient relevance for future adaptive measurement development’.

3. You state that new instruments should be developed. I wonder if complementing already existing ones would be possible.

We thank the Reviewer for this comment. As stated in the Introduction, our aim was to work on the structure of HRQoL by identifying and selecting the most relevant HRQoL domains in MS. Our intention is not to disregard existing instruments, but to build upon them, integrating the domains they already cover with additional areas that emerged as underrepresented in our analyses and stakeholder discussions (e.g., work participation).

When we refer to the need for new instruments, we are not implying that measurement development is part of the present work. Rather, this study provides the conceptual groundwork for future initiatives that will aim to complement and extend existing MS-specific measures. Such future developments will be based on the framework established here, ensuring that subsequent tools are both conceptually comprehensive and aligned with patients’ lived experiences.

4. Was it possible for all potential participants to meet virtually? Did all have access to the internet? If not, was there a plan how they could have been assisted?

(e.g. drive home to them and offer proper assistance?)

I confirm that all participants met virtually and that they all have access to the Internet.

5. Did participants sign a consent form for being audiotaped?

Yes, they did.

6. Please structure your paper that it is easier to read and to follow. It is a very long manuscript. Please try to make it shorter, at the same time clearer.

We thank the Reviewer for their helpful suggestion. Following their recommendation, we carefully revised the manuscript to improve its readability, coherence, and overall structure. We streamlined the Introduction, Methods and Results sections by removing redundant or overly detailed descriptions and improved transitions between the systematic review and qualitative phases. In addition, we incorporated a more structured comparison between the domains assessed by existing questionnaires and the categories that emerged from the qualitative analysis, in order to better address the aims of our study and respond to the reviewers’ comments. We also judged the “Factors Influencing HRQoL” subsection to be less relevant to the specific objectives of the manuscript, and therefore removed it, which allowed us to shorten the text (5993 words instead of 6905) and, we hope, improve its clarity. In line with this overall effort, we also substantially revised the Discussion to enhance its focus, coherence, and alignment with the study’s revised structure.

7. Table 1: I would have expected more domains.

Thank you for this observation. Table 1 intentionally reports only the main categories of the HRQoL domains. All categories are then expanded and articulated into detailed subcategories in Tables 2–4, and further illustrated in the audit trail (S6 File). Our aim was to keep Table 1 concise and readable, while providing full domain granularity in the subsequent tables and supplementary materials.

8. Please add list of abbreviations.

We have now added the list of abbreviations.

9. Persons with MS were included and health professionals. Support persons were not included. They can provide great information regarding the QoL of PwMS. They should have been included in the focus groups.

We agree with the Reviewer’s comment. As acknowledged in the Limitations section, we did not involve caregivers due to limited resources. We fully recognize, however, that support persons can provide valuable complementary perspectives on the QoL of PwMS, particularly regarding aspects of daily functioning and relational dynamics that may be less visible to patients themselves. We have now further clarified this point in the manuscript and emphasized that future research should incorporate caregivers’ perspectives to strengthen the comprehensiveness and applicability of the conceptual framework, as follows: ‘Moreover, caregivers were not included, limiting insight into indirect or externally observed aspects of HRQoL’.

10. If you want to submit this as a paper, be aware that it should be much shorter.

You can put information, which is maybe too detailed, to the Appendix. Not everything has to be on the main paper.

Please, see the reply to point 6 above.

11. You identified physical, psychological and social domains as important.

We already know this from former qualitative and quantitative research.

We thank the Reviewer for this important and thoughtful comment, which prompted us to reflect deeply on the clarity and contribution of our work and ultimately led to substantial improvements throughout the manuscript. We agree that the Physical, Psychological, and Social domains are well-established in the MS HRQoL literature, and we do not present them as novel findings. As clarified in our response to Comment 19, the contribution of our study lies instead in the refinement, specification, and extension of these domains. Through the integration of qualitative data, we identified additional subdomains and nuances—such as work-related challenges, financial concerns, contextual influences, and aspects of self-perception—that are not consistently or comprehensively captured in existing MS-specific instruments.

To better highlight this contribution, we substantially revised the manuscript. As noted in our response to point 6 above, we restructured several sections to improve clarity and readability, streamlined redundant content, and strengthened transitions between the systematic review and qualitative components. Importantly, to more clearly convey what is new in our study and why it matters, we introduced a dedicated methodological section (“Cross-Mapping of HRQoL Instruments and Qualitative Findings”). This section systematically compares qualitative categories and subcategories with the items of MS-specific HRQoL instruments, thereby identifying which aspects of the lived experience of PwMS are sufficiently represented and which remain underrepresented or absent.

We incorporated the outcomes of this cross-mapping into the Results section and revised the Discussion accordingly. These changes more clearly articulate the novelty and relevance of our work: not the identification of new overarching domains, but the addition of conceptual granularity and the systematic identification of measurement gaps that can inform the refinement of existing HRQoL instruments or the development of new patient-centered tools.

We are grateful to the Reviewer for raising this point, which significantly strengthened the focus and contribution of the manuscript.

12. You state ‘The growing interest in CAT instruments in HRQoL research has led to the development of a range of CAT instruments by the Patient-Reported Outcomes Measurement Information System (PROMIS) supported by the National Institute of Health [20], which currently offers 3 to 10 items for domains such as fatigue, physical functioning, pain, depression, anxiety, and social functioning (www.nihpromis.org). For the assessment of multidimensional constructs with correlated dimensions, the Multidimensional Computer Adaptive Testing (MCAT) approach is more appropriate, but, as far as we know, there is few evidence of HRQoL instruments using such a methodology to fixed-length HRQOL questionnaires, including the MS domain [21-24]. To address this gap, we took a comprehensive approach based on a literature review and informed by the patient voice to identify relevant HRQoL dimensions that ensure both content validity and clinical utility of the assessment [20,25,26].’

My question: did you use MCAT by facilitating the focus groups online?

No, we did not use MCAT to facilitate the online focus groups. As already stated in the manuscript, the aim of our work was to define a well-structured domain framework of HRQoL in multiple sclerosis. Such a well-defined domain structure may be useful for several future research directions, including the development of psychometrically sound item banks that would allow for accurate, efficient, and individualized assessment, potentially through CAT or MCAT approaches. Therefore, MCAT was not applied in the present study but is envisioned as a possible methodological development building on our findings.

13. I don’t understand why identifying quantitative questionnaires used was necessary first before the qualitative focus group discussions.

Please clarify.

We thank the Reviewer for their comment. The systematic identification of MS-specific HRQoL questionnaires was conducted first to provide an evidence-based foundation for the qualitative phase. The purpose was to extract HRQoL domains already represented in the literature and use them to develop the focus group guides.

As described in the Methods, ‘[…] the deductive process, informed by findings from the literature review (existing HRQoL domains), applied category labels consistent with the literature when appropriate.’ This sequential design ensured that focus group discussions with PwMS and clinical professionals were grounded in the existing conceptual framework, while still allowing new or refined domains to emerge inductively from participants’ perspectives.

14. You state that your goal was:

‘ 1. to conduct a systematic literature review to identify the most relevant HRQoL domains in MS; 2) to discuss the identified HRQoL domains with PwMS and eventually supplement them with additional relevant domains.’

What additional categories would you add to understanding HRQOL?

Please, see our replies to point 6 and 11 above, and point 19 of Reviewer 3.

15. You state ‘To identify additional studies, we also reviewed published systematic reviews and the reference lists of the selected articles’. Good job!

We thank very much this Reviewer for their appreciation.

16. You picked PwMS who should be in the FG interviews? Is this correct? Would it have been possible you would have allowed random participants?

Please specify ‘Investigators at each participating centre identified potentially eligible PwMS from their database and invited them to participate in the study’.

We thank the Reviewer for this comment. As stated in the manuscript, FGM participants were selected through purposive sampling, which is an established approach in qualitative research. This method was chosen to ensure a broad range of perspectives and experiences in relation to HRQoL, including variation in age, disease duration, disability level, and psychosocial background. A random selection would not have been appropriate for the qualitative design of this study, whose aim was conceptual exploration and diversity of viewpoints, rather than statistical representativeness.

17. Regarding Inclusion/Exclusion Criteria:

Try to say in Methods/Study Design/Study Participants who were included and what the inclusion criteria were.

The eligibility criteria for PwMS and HPs are reported in the FGM enrolment procedure.

18. FGM Conduct: that paragraph could be said in 2 sentences. Try to reduce text.

Following this Reviewer’s suggestion, we have now shortened the paragraph, as follows: ‘Each focus group meeting (FGM) included 6–10 participants and two facilitators (AMG and GDD) and was conducted online via Zoom to enhance accessibility and feasibility for PwMS. Sessions followed a semi-structured guide covering pre-specified topics, were audio-recorded, fully transcribed, and subsequently reviewed by participants for validation’.

19. Try to create one chapter called Methods and Study Design and describe all Methods used in this study.

Describe under Analysis all Methods you used.

You have Methods for the focus groups and methods for identifying literature on the search engines.

Following this Reviewer’s suggestion, we have now revised the structure of the Methods section to present all methodological components under a single heading entitled “Methods and Study Design.”

20. Under results you already describe what you identified with your methods used. That’s good.

We thank very much the Reviewer for their appreciation.

For the qualitative study don’t mix participants and setting with the results. Under results present all results, under Methods present all Methods used (thus for your focus groups as well).

We thank the Reviewer for this important comment regarding the separation of Methods and Results. We fully agree that the procedural aspects of a qualitative study—such as recruitment strategy, inclusion/exclusion criteria, setting, and data collection methods—belong to the Methods section.

However, we would like to clarify that the outcome of participant recruitment—namely the number of participants actually enrolled, characteristics of the final sample, and any refusals or drop-outs—represents a result of the recruitment process, not a methodological detail known a priori. Reporting these outcomes in the Results section is consistent with standard guidance for qualitative research reporting. For example, O’Brien et al. (2014), in the widely cited Standards for Reporting Qualitative Research (SRQR), state: “In the Results section, describe the participants of the study. […] Where the Sampling Strategy section in the Methods section described the aims and strategies, this section describes the results of those efforts.” (O’Brien BC, Harris IB, Beckman TJ, Reed DA, Cook DA. Standards for Reporting Qualitative Research: A Synthesis of Recommendations. Acad Med. 2014;89(9):1245 1251. doi:10.1097/ACM.0000000000000388).

Similarly, reporting the actual composition of the sample is emphasized by the COREQ framework: “The Results sectio

Attachment Submitted filename: Response to Reviewers.docx

# Peer review: Decision Letter 1

**DOI:** 10.1371/journal.pone.0349034.r003

**Authors:** Homa Seyedmirzaei

9 Feb 2026

PONE-D-25-43699R1

Quality of life domains revised by people with multiple sclerosis and healthcare professionals for adaptive measure development

PLOS One

Dear Dr. Giordano,

Thank you for submitting your manuscript to PLOS ONE. After careful consideration, we feel that it has merit but does not fully meet PLOS ONE’s publication criteria as it currently stands. Therefore, we invite you to submit a revised version of the manuscript that addresses the points raised during the review process.

Please submit your revised manuscript by Mar 26 2026 11:59PM. If you will need more time than this to complete your revisions, please reply to this message or contact the journal office at plosone@plos.org. When you're ready to submit your revision, log on to https://www.editorialmanager.com/pone/ and select the 'Submissions Needing Revision' folder to locate your manuscript file.

Please include the following items when submitting your revised manuscript:

- A letter that responds to each point raised by the academic editor and reviewer(s). You should upload this letter as a separate file labeled 'Response to Reviewers'.
- A marked-up copy of your manuscript that highlights changes made to the original version. You should upload this as a separate file labeled 'Revised Manuscript with Track Changes'.
- An unmarked version of your revised paper without tracked changes. You should upload this as a separate file labeled 'Manuscript'.

If you would like to make changes to your financial disclosure, please include your updated statement in your cover letter. Guidelines for resubmitting your figure files are available below the reviewer comments at the end of this letter.

If applicable, we recommend that you deposit your laboratory protocols in protocols.io to enhance the reproducibility of your results. Protocols.io assigns your protocol its own identifier (DOI) so that it can be cited independently in the future. For instructions see: https://journals.plos.org/plosone/s/submission-guidelines#loc-laboratory-protocols. Additionally, PLOS ONE offers an option for publishing peer-reviewed Lab Protocol articles, which describe protocols hosted on protocols.io. Read more information on sharing protocols at https://plos.org/protocols?utm_medium=editorial-email&utm_source=authorletters&utm_campaign=protocols.

We look forward to receiving your revised manuscript.

Kind regards,

Homa Seyedmirzaei, M.D

Academic Editor

PLOS One

Journal Requirements:

If the reviewer comments include a recommendation to cite specific previously published works, please review and evaluate these publications to determine whether they are relevant and should be cited. There is no requirement to cite these works unless the editor has indicated otherwise.

Please review your reference list to ensure that it is complete and correct. If you have cited papers that have been retracted, please include the rationale for doing so in the manuscript text, or remove these references and replace them with relevant current references. Any changes to the reference list should be mentioned in the rebuttal letter that accompanies your revised manuscript. If you need to cite a retracted article, indicate the article’s retracted status in the References list and also include a citation and full reference for the retraction notice.

[Note: HTML markup is below. Please do not edit.]

Reviewers' comments:

Reviewer's Responses to Questions

**Comments to the Author**

1. If the authors have adequately addressed your comments raised in a previous round of review and you feel that this manuscript is now acceptable for publication, you may indicate that here to bypass the “Comments to the Author” section, enter your conflict of interest statement in the “Confidential to Editor” section, and submit your "Accept" recommendation.

Reviewer #2: All comments have been addressed

Reviewer #4: (No Response)

**********

2. Is the manuscript technically sound, and do the data support the conclusions?

The manuscript must describe a technically sound piece of scientific research with data that supports the conclusions. Experiments must have been conducted rigorously, with appropriate controls, replication, and sample sizes. The conclusions must be drawn appropriately based on the data presented.

Reviewer #2: Yes

Reviewer #4: (No Response)

**********

3. Has the statistical analysis been performed appropriately and rigorously?

Reviewer #2: Yes

Reviewer #4: (No Response)

**********

4. Have the authors made all data underlying the findings in their manuscript fully available?

The PLOS Data policy requires authors to make all data underlying the findings described in their manuscript fully available without restriction, with rare exception (please refer to the Data Availability Statement in the manuscript PDF file). The data should be provided as part of the manuscript or its supporting information, or deposited to a public repository. For example, in addition to summary statistics, the data points behind means, medians and variance measures should be available. If there are restrictions on publicly sharing data—e.g. participant privacy or use of data from a third party—those must be specified.

Reviewer #2: Yes

Reviewer #4: (No Response)

**********

5. Is the manuscript presented in an intelligible fashion and written in standard English?

PLOS ONE does not copyedit accepted manuscripts, so the language in submitted articles must be clear, correct, and unambiguous. Any typographical or grammatical errors should be corrected at revision, so please note any specific errors here.

Reviewer #2: Yes

Reviewer #4: (No Response)

**********

6. Review Comments to the Author

Reviewer #2: (No Response)

Reviewer #4: This is a well-executed and carefully revised manuscript. The authors have addressed the comments from previous reviewers in a thoughtful and transparent manner, and the methodological approach is appropriate and rigorous for the stated aims. The sequential integration of a systematic review, qualitative focus groups, and cross-mapping of domains provides a solid foundation for refining HRQoL constructs in multiple sclerosis. Overall, the study is methodologically sound and close to being suitable for publication.

I have a few minor comments that are intended to further strengthen the conceptual clarity and methodological transparency of an otherwise strong manuscript.

1) Treatment Burden concept: While treatment-related aspects are included within the physical domain (e.g., impact of DMTs on the body), the broader concept of treatment burden could be more explicitly acknowledged. In chronic diseases such as multiple sclerosis, treatment burden extends beyond biological effects and often encompasses cognitive, emotional, organizational, financial, and social demands, which may influence HRQoL across physical, psychological, and social domains simultaneously. I suggest briefly acknowledging treatment burden in the Discussion as a cross-cutting factor that modifies HRQoL across domains, even if it is not proposed as a separate domain. This would further strengthen the patient-centered perspective of the framework and align well with the manuscript’s emphasis on adaptive and personalized HRQoL assessment.

2) Role of social support: Although social support is appropriately included within the social domain, its buffering role in mitigating treatment burden, disability, and psychosocial stress could be more explicitly highlighted. This may be particularly relevant when discussing future adaptive or digital HRQoL tools, where social resources can substantially shape patients’ capacity to cope with disease and treatment demands.

3) Qualitative rigor and saturation: The qualitative methodology is clearly described and robust. For completeness, the authors may consider explicitly stating whether thematic saturation was considered and achieved during the focus group process, as this is commonly expected in qualitative research reporting.

Given that the focus group guides were informed by domains identified in existing HRQoL instruments, it may be helpful to briefly acknowledge the potential for a framing effect, even though this was mitigated by the combined deductive–inductive coding approach. Additionally, a short comment on the transferability of findings beyond the Italian healthcare context would further enhance methodological transparency.

4) Caregiver perspectives The absence of caregivers or informal support persons is appropriately mentioned as a limitation. Given the relevance of social support and treatment burden, the authors may wish to emphasize that caregiver perspectives represent an important methodological and conceptual direction for future research.

The suggested points are primarily conceptual and clarificatory in nature and do not require additional analyses. Addressing them would further enhance the clarity, completeness, and real-world relevance of the proposed HRQoL framework.

**********

7. PLOS authors have the option to publish the peer review history of their article (what does this mean?). If published, this will include your full peer review and any attached files.

If you choose “no”, your identity will remain anonymous but your review may still be made public.

**Do you want your identity to be public for this peer review?** For information about this choice, including consent withdrawal, please see our Privacy Policy.

Reviewer #2: **Yes:** Gianmarco Abbadessa

Reviewer #4: No

**********

[NOTE: If reviewer comments were submitted as an attachment file, they will be attached to this email and accessible via the submission site. Please log into your account, locate the manuscript record, and check for the action link "View Attachments". If this link does not appear, there are no attachment files.]

To ensure your figures meet our technical requirements, please review our figure guidelines: https://journals.plos.org/plosone/s/figures

You may also use PLOS’s free figure tool, NAAS, to help you prepare publication quality figures: https://journals.plos.org/plosone/s/figures#loc-tools-for-figure-preparation.

NAAS will assess whether your figures meet our technical requirements by comparing each figure against our figure specifications.

# Author comment: Author response to Decision Letter 2

**DOI:** 10.1371/journal.pone.0349034.r004

12 Mar 2026

Reviewer #4:

This is a well-executed and carefully revised manuscript. The authors have addressed the comments from previous reviewers in a thoughtful and transparent manner, and the methodological approach is appropriate and rigorous for the stated aims. The sequential integration of a systematic review, qualitative focus groups, and cross-mapping of domains provides a solid foundation for refining HRQoL constructs in multiple sclerosis. Overall, the study is methodologically sound and close to being suitable for publication.

Reply: We thank very much the Reviewer for their appreciation.

I have a few minor comments that are intended to further strengthen the conceptual clarity and methodological transparency of an otherwise strong manuscript.

1) Treatment Burden concept: While treatment-related aspects are included within the physical domain (e.g., impact of DMTs on the body), the broader concept of treatment burden could be more explicitly acknowledged. In chronic diseases such as multiple sclerosis, treatment burden extends beyond biological effects and often encompasses cognitive, emotional, organizational, financial, and social demands, which may influence HRQoL across physical, psychological, and social domains simultaneously. I suggest briefly acknowledging treatment burden in the Discussion as a cross-cutting factor that modifies HRQoL across domains, even if it is not proposed as a separate domain. This would further strengthen the patient-centered perspective of the framework and align well with the manuscript’s emphasis on adaptive and personalized HRQoL assessment.

Reply: We thank the Reviewer for this insightful comment. Following the Reviewer’s suggestion, we have now more explicitly acknowledged the broader concept of treatment burden in the manuscript. First, we strengthened this aspect in the Results section (Psychological Domain), where it was previously less explicitly represented. In particular, we added the following text to highlight the psychological impact associated with the ongoing management of the disease:

“Participants also highlighted that the ongoing management of the disease–including therapies, diagnostic tests, and regular medical appointments–can itself generate psychological strain: ‘One aspect that hasn't been mentioned is the impact–not just physical, but also the effects of therapies and the planning of the entire diagnostic process on daily life… individuals suddenly feel they are in need of support from others and require assistance from healthcare professionals.’ (ID: 6H).”

Second, we revised the Discussion to explicitly acknowledge treatment burden as a cross-cutting factor influencing HRQoL across domains. In the section Integrating Implications Across Domains, we added the following statement: “In addition, our findings suggest that treatment burden—including the cognitive, emotional, and organizational demands associated with therapies, medical appointments, and disease monitoring—may act as a cross-cutting factor influencing HRQoL simultaneously across physical, psychological, and social domains.”

These additions aim to better reflect the multidimensional nature of treatment burden and its relevance for patient-centered and adaptive HRQoL assessment in PwMS.

2) Role of social support: Although social support is appropriately included within the social domain, its buffering role in mitigating treatment burden, disability, and psychosocial stress could be more explicitly highlighted. This may be particularly relevant when discussing future adaptive or digital HRQoL tools, where social resources can substantially shape patients’ capacity to cope with disease and treatment demands.

Reply: We thank the Reviewer for their comment. According to Leidy’s definition of HRQOL as the “subjective perception of the impact of disease and treatment across the physical, psychological, social functioning and well-being” [ref #10], our objective was to explore how MS affects each of these core HRQOL domains. Within this conceptual framework, social support represents a key component of the Social Domain (Table 4). In the previous version of the manuscript, we had also included a separate theme, called ‘Factors influencing HRQOL’, including the following sub-themes: Personal Factors; Health-Related Factors; Life-Related Factors; Relational Factors; and Contextual Factors.

Following the Reviewers’ suggestions, we reconsidered the alignment of this section with the primary aim of the study. As our objective was specifically focused on describing the impact of multiple sclerosis on HRQoL domains, we agreed that this additional thematic area extended beyond the core scope of the manuscript. For this reason, we removed the “Factors Influencing HRQoL” subsection, which also contributed to improving the clarity and conciseness of the text.

3) Qualitative rigor and saturation: The qualitative methodology is clearly described and robust. For completeness, the authors may consider explicitly stating whether thematic saturation was considered and achieved during the focus group process, as this is commonly expected in qualitative research reporting.

Reply: We thank the Reviewer for this comment. Data saturation was discussed and results were reported in the Consolidated criteria for reporting qualitative studies (COREQ) checklist (S3 File), as follows: “Yes. Data saturation was discussed during the analysis process. After conducting four focus group meetings, researchers observed recurring themes and minimal emergence of new concepts, suggesting that thematic saturation had been reached. The decision to stop data collection was based on the redundancy of the information and the consistency of themes across groups.”

Given that the focus group guides were informed by domains identified in existing HRQoL instruments, it may be helpful to briefly acknowledge the potential for a framing effect, even though this was mitigated by the combined deductive–inductive coding approach.

Reply: We thank the Reviewer for this thoughtful comment. The focus group guides were intentionally informed by domains identified in existing HRQoL instruments, as the aim of the study was to build on and situate our findings within the established literature. This deductive grounding was considered important to ensure conceptual alignment with prior work and to facilitate comparability across studies. At the same time, we acknowledge the potential for a framing effect inherent in this approach. To mitigate this risk, we adopted a combined deductive–inductive coding strategy. While initial codes were informed by existing frameworks, the analysis remained open to the identification of themes and subthemes emerging directly from the data. In our view, this inductive component ensured that novel, unanticipated aspects of participants’ experiences could be recognized and incorporated into the findings, even when not captured by existing HRQoL instruments.

Additionally, a short comment on the transferability of findings beyond the Italian healthcare context would further enhance methodological transparency.

Reply: Following the Reviewer’s suggestion, we have now slightly revised the Limitations, as follows: ‘Transferability of our findings beyond the Italian healthcare system requires further investigation’.

4) Caregiver perspectives - The absence of caregivers or informal support persons is appropriately mentioned as a limitation. Given the relevance of social support and treatment burden, the authors may wish to emphasize that caregiver perspectives represent an important methodological and conceptual direction for future research.

Reply: We agree with this Reviewer. Following their suggestion, we have slightly revised the Limitations, as follows: ‘Including caregivers in future research would allow for a more comprehensive and multidimensional understanding of these aspects, enriching the interpretation of HRQoL through complementary perspectives and strengthening the overall validity of the findings’.

Attachment Submitted filename: Response to reviewers_9-3-26.docx

# Peer review: Decision Letter 2

**DOI:** 10.1371/journal.pone.0349034.r005

**Authors:** Amit Kumar

23 Apr 2026

Quality of life domains revised by people with multiple sclerosis and healthcare professionals for adaptive measure development

PONE-D-25-43699R2

Dear Dr. Giordano,

We’re pleased to inform you that your manuscript has been judged scientifically suitable for publication and will be formally accepted for publication once it meets all outstanding technical requirements.

Within one week, you’ll receive an e-mail detailing the required amendments. When these have been addressed, you’ll receive a formal acceptance letter and your manuscript will be scheduled for publication.

An invoice will be generated when your article is formally accepted. Please note, if your institution has a publishing partnership with PLOS and your article meets the relevant criteria, all or part of your publication costs will be covered. Please make sure your user information is up-to-date by logging into Editorial Manager at Editorial Manager® and clicking the ‘Update My Information' link at the top of the page. For questions related to billing, please contact billing support.

If your institution or institutions have a press office, please notify them about your upcoming paper to help maximize its impact. If they’ll be preparing press materials, please inform our press team as soon as possible -- no later than 48 hours after receiving the formal acceptance. Your manuscript will remain under strict press embargo until 2 pm Eastern Time on the date of publication. For more information, please contact onepress@plos.org.

Kind regards,

Amit Kumar

Academic Editor

PLOS One

Additional Editor Comments (optional):

The paper can be accepted.

Reviewers' comments:

Reviewer's Responses to Questions

**Comments to the Author**

1. If the authors have adequately addressed your comments raised in a previous round of review and you feel that this manuscript is now acceptable for publication, you may indicate that here to bypass the “Comments to the Author” section, enter your conflict of interest statement in the “Confidential to Editor” section, and submit your "Accept" recommendation.

Reviewer #4: All comments have been addressed

**********

2. Is the manuscript technically sound, and do the data support the conclusions?

The manuscript must describe a technically sound piece of scientific research with data that supports the conclusions. Experiments must have been conducted rigorously, with appropriate controls, replication, and sample sizes. The conclusions must be drawn appropriately based on the data presented.

Reviewer #4: Yes

**********

3. Has the statistical analysis been performed appropriately and rigorously?

Reviewer #4: Yes

**********

4. Have the authors made all data underlying the findings in their manuscript fully available?

The PLOS Data policy requires authors to make all data underlying the findings described in their manuscript fully available without restriction, with rare exception (please refer to the Data Availability Statement in the manuscript PDF file). The data should be provided as part of the manuscript or its supporting information, or deposited to a public repository. For example, in addition to summary statistics, the data points behind means, medians and variance measures should be available. If there are restrictions on publicly sharing data—e.g. participant privacy or use of data from a third party—those must be specified.

Reviewer #4: Yes

**********

5. Is the manuscript presented in an intelligible fashion and written in standard English?

PLOS ONE does not copyedit accepted manuscripts, so the language in submitted articles must be clear, correct, and unambiguous. Any typographical or grammatical errors should be corrected at revision, so please note any specific errors here.

Reviewer #4: Yes

**********

6. Review Comments to the Author

Please use the space provided to explain your answers to the questions above. You may also include additional comments for the author, including concerns about dual publication, research ethics, or publication ethics. (Please upload your review as an attachment if it exceeds 20,000 characters)

Reviewer #4: All of my comments have been adequately addressed, and I believe the manuscript is acceptable for publication without further changes

**********

7. PLOS authors have the option to publish the peer review history of their article (what does this mean?). If published, this will include your full peer review and any attached files.

If you choose “no”, your identity will remain anonymous but your review may still be made public.

**Do you want your identity to be public for this peer review?** For information about this choice, including consent withdrawal, please see our Privacy Policy.

Reviewer #4: No

**********

# Editor report: Acceptance letter

**DOI:** 10.1371/journal.pone.0349034.r006

**Authors:** Amit Kumar

PONE-D-25-43699R2

PLOS One

Dear Dr. Giordano,

I'm pleased to inform you that your manuscript has been deemed suitable for publication in PLOS One. Congratulations! Your manuscript is now being handed over to our production team.

At this stage, our production department will prepare your paper for publication. This includes ensuring the following:

* All references, tables, and figures are properly cited

* All relevant supporting information is included in the manuscript submission,

* There are no issues that prevent the paper from being properly typeset

You will receive further instructions from the production team, including instructions on how to review your proof when it is ready. Please keep in mind that we are working through a large volume of accepted articles, so please give us a few days to review your paper and let you know the next and final steps.

Lastly, if your institution or institutions have a press office, please let them know about your upcoming paper now to help maximize its impact. If they'll be preparing press materials, please inform our press team within the next 48 hours. Your manuscript will remain under strict press embargo until 2 pm Eastern Time on the date of publication. For more information, please contact onepress@plos.org.

You will receive an invoice from PLOS for your publication fee after your manuscript has reached the completed accept phase. If you receive an email requesting payment before acceptance or for any other service, this may be a phishing scheme. Learn how to identify phishing emails and protect your accounts at https://explore.plos.org/phishing.

If we can help with anything else, please email us at customercare@plos.org.

Thank you for submitting your work to PLOS ONE and supporting open access.

Kind regards,

PLOS ONE Editorial Office Staff

on behalf of

Dr. Amit Kumar

Academic Editor

PLOS One
